# Global, regional, and national burden of myocarditis and its attributable risk factors in 204 countries and territories from 1990 to 2021: updated systematic analysis

**DOI:** 10.3389/fpubh.2025.1542921

**Published:** 2025-04-28

**Authors:** Jiajia Ren, Wanyuan Liu, Xuting Jin, Chuchu Zhang, Xi Xu, Guorong Deng, Xiaoming Gao, Jiamei Li, Ruohan Li, Xiaoling Zhang, Yanli Hou, Gang Wang

**Affiliations:** ^1^Department of Critical Care Medicine, The Second Affiliated Hospital of Xi’an Jiaotong University, Xi’an, China; ^2^Key Laboratory of Surgical Critical Care and Life Support, Xi'an Jiaotong University, Ministry of Education, Xi'an, China

**Keywords:** myocarditis, Global Burden of Disease, incidence, prevalence, death, disability-adjusted life years

## Abstract

**Background:**

Comprehending the current epidemiological trends and risk factors of myocarditis is crucial for guiding future targeted prevention and treatment strategies.

**Methods:**

Utilizing data from the Global Burden of Diseases, Injuries, and Risk Factors Study 2021, we conducted a secondary analysis of the incidence, prevalence, death, and disability-adjusted life years (DALYs) of myocarditis by sex, age group and socio-demographic index (SDI) across 204 countries and territories from 1990 to 2021. And non-optimal temperatures, defined as same-day exposure to ambient temperatures deviating from the minimum death risk threshold, were identified as risk-factors for myocarditis-related death and DALYs.

**Results:**

From 1990 to 2021, the global prevalence of myocarditis increased from 320,623 (95% uncertainty interval: 268,557 to 371,912) to 505,030 (432,295 to 587,819). Concurrently, the age-standardized prevalence rate (ASPR) per 100,000 people also saw a slight increase (no statistical significance) from 6.35 (5.37 to 7.36) to 6.41 (5.48 to 7.44). However, the age-standardized incidence rate (ASIR), age-standardized death rate (ASDR) and age-standardized DALY rate (ASYR) exhibited declines, with estimated annual percentage changes of −0.20 (−0.23 to −0.17), −1.37 (−1.81 to −0.92) and −1.71 (−1.95 to −1.46), respectively. SDI quintile analysis showed that the high SDI quintile had the highest ASIR and ASPR, while the middle and high-middle SDI quintiles exhibited the highest ASDR and ASYR. Furthermore, the burden of myocarditis was notably high among males and older adult populations. Non-optimal temperature, particularly low temperature, emerged as a key risk factor for myocarditis-related ASDR and ASYR.

**Conclusion:**

Although the ASIR, ASDR and ASYR for myocarditis exhibited decreasing trends from 1990 to 2019, further efforts are needed to develop targeted public health strategies, especially for low SDI regions, males, and older adult populations.

## Introduction

Myocarditis, characterized by inflammation of the myocardium, presents a diverse array of symptoms including fever, fatigue, decreased exercise tolerance, palpitations, chest pain, syncope, dyspnea, and other nonspecific manifestations (1, 2). If not promptly treated, or in severe cases, myocarditis can progress to heart failure, ventricular arrhythmias, and even death (3–5). Pathological investigations reveal that myocarditis accounts for approximately 8.6 to 12% of sudden cardiac deaths in young individuals with structurally normal hearts (6, 7). The clinical repercussions of myocarditis extend beyond immediate morbidity and mortality, encompassing long-term sequelae such as dilated cardiomyopathy. Up to 30% of biopsy-confirmed myocarditis cases can evolve into dilated cardiomyopathy, which is currently the leading cause of heart transplantation (8, 9). The diverse clinical presentations and potential complications associated with myocarditis pose substantial challenges to healthcare systems worldwide.

Myocarditis can be caused by various infectious agents, including bacteria, viruses, fungi, and protozoa. Among viral infections, coxsackievirus, adenovirus, and enterovirus are the primary culprits (3, 10, 11). Additionally, parvovirus B19, human herpesvirus-6, hepatitis C virus, Epstein–Barr virus, and cytomegalovirus have been implicated in myocarditis cases (9, 12–15). Recent outbreaks, such as SARS-CoV-2, have underscored a notable cause of myocarditis, with studies indicating a fifteen-fold increase in myocarditis risk following infection (16, 17). Beyond infectious triggers, myocarditis can also result from drug reactions, toxin exposure, hypersensitivity responses, and autoimmune disorders (1, 8). The interplay of infectious and non-infectious factors contributes to the intricate epidemiology of myocarditis.

The Global Burden of Diseases, Injuries, and Risk Factors Study (GBD) 2021 provides a comprehensive assessment of 371 diseases and injuries across 204 countries and territories from 1990 to 2021 (18). Similar GBD-based analyses have been conducted for other critical conditions, including chronic obstructive pulmonary disease and esophageal cancer, revealing region-specific burden patterns (19–21). These studies highlight the utility of GBD data in informing public health priorities. According to the GBD 2019 analysis, there were 1,265,800 incident cases, 32,450 deaths, and 12.81 disability-adjusted life years (DALYs) attributed to myocarditis worldwide in 2019. Over the period from 1990 to 2019, the estimated annual percentage change (EAPC) for age-standardized incidence, death and DALYs rates (ASIR, ASDR, ASYR) decreased by 0.23, 0.09 and 1.19%, respectively (22). However, this analysis did not explore specific risk factors for myocarditis. Another study examining the burden of cardiomyopathy and myocarditis among individuals aged 60–89 years found that high systolic blood pressure and alcohol consumption were the primary risk factors contributing to death associated with these conditions (23). Yet, risk factor analyses specific to myocarditis remain underexplored. Leveraging the continuously updated GBD dataset, we aim to address this gap. Using the latest GBD 2021 findings, our study provides comprehensive estimates of myocarditis burden, including incidence, prevalence, deaths, and DALYs, along with its risk factors. These estimates are stratified by year, location, sex, age, and Socio-Demographic Index (SDI) levels. This study aims to provide evidence-based insights to inform public health policies, resource allocation, and the development of preventive strategies to mitigate the impact of myocarditis.

## Methods

### Data sources

The GBD 2021 is a comprehensive study that provides up-to-date information on the distribution and burden of 371 diseases and injuries, 288 causes of death and 88 risk factors at the global level, across 7 super-regions, 21 regions, 204 countries and territories, and 811 subnational locations from 1990 to 2021 (18, 24, 25). The study design and methods employed in GBD 2021 have been thoroughly detailed in previous publications (18, 24, 25).

To evaluate the non-fatal burden of myocarditis in GBD 2021, incidence and prevalence were estimated using inpatient hospital data stratified by sex, location, year, and age. These estimations were performed using the DisMod-MR Bayesian meta-regression model (18). Disability weights, ranging from 0 (perfect health) to 1 (equivalent to death), were used to quantify the disease’s impact on health. The prevalence rate for each sequela was multiplied by the corresponding disability weight to calculate the years lived with disability (18).

Estimates of deaths attributed to myocarditis in GBD 2021 were derived from vital registration data. A standard model, known as the Cause of Death Ensemble model, was utilized to calculate the deaths across different demographics including age, sex, location, and year (25). Years of life lost were computed by multiplying the number of deaths by the standard life expectancy. Additionally, DALYs, which quantify the total years lost due to illness, disability or premature death, were calculated as the sum of years lived with disability and years of life lost (25).

To compute deaths and DALYs attributed to specific risk factors, GBD 2021 implemented a comparative risk assessment framework (24). Within GBD 2021, the risk factors contributing to deaths and DALYs associated with myocarditis included high temperature and low temperature. The non-optimal temperature risk factor is defined as same-day exposure to ambient temperature that deviate from the temperature with the minimum death risk. Specifically, the theoretical minimum risk exposure level (TMREL) for non-optimal temperature was determined as the temperature associated with the lowest overall death attributed to this risk in a specific location and year. Due to variations in exposure–response curves across different mean annual temperature zones, as well as spatiotemporal differences in cause of death, TMRELs was assessed by year and location rather than a globally consistent TMREL. High temperature exposure is defined as exposure to temperatures hotter than the TMREL, and low temperature is defined as temperatures colder than the TMREL (24).

Data regarding non-optimal temperature were mainly derived from the ERA5 reanalysis data, developed by the European Medium-Range Weather Forecasting Center. The meta-regression framework, executed via the Bayesian, regularised, trimmed tool, was employed to incorporate myocarditis death and temperature exposure data, establish relative risk-response relationship curves for temperature exposure, and compute population-attributable fractions (PAFs), which indicated the relative contribution of non-optimal temperature to the myocarditis burden (24). To assess the disease burden associated with non-optimal temperature, the corresponding PAF estimates were multiplied by the overall myocarditis burden (24). Uncertainty intervals (UIs) were determined using the 2.5th and 97.5th percentiles from a 1,000-draw distribution for each metric.

### Case definition

Myocarditis, characterized by inflammation of the myocardium, can be caused by viral infections, autoimmune conditions, and various non-ischemic factors. Cases of myocarditis are identified through the International Classification of Diseases (ICD) codes, which include 074.2, 074.23, 422–422.99, 429.0–429.1 for ICD-9, as well as B33.2-B33.20, B33.22-B33.24, I40-I41.8, I51.4-I51.6 for ICD-10 (18).

### Socio-demographic index

The SDI serves as a composite indicator reflecting the social and economic conditions influencing health outcomes within a given region. It is calculated from three key components: the total fertility rate among women under 25, the average years of schooling for individuals aged 15 and above, and the lag-distributed income per capita. The SDI ranges from 0 to 100, with higher values indicating more favorable socioeconomic conditions. Based on quintiles of SDI in 2021, countries and regions were categorized into five groups: low SDI, low-middle SDI, middle SDI, high-middle SDI and high SDI (18). Additionally, the 204 countries and territories were classified into 21 distinct GBD geographic regions. For a detailed breakdown of countries and territories within each GBD region, please refer to Supplementary Table S1.

### Statistical analyses

Using the Global Health Data Exchange platform, we conducted a secondary analysis of GBD 2021 data to estimate myocarditis burden metrics globally, across 21 regions, and 204 countries and territories from 1990 to 2021. The extracted outcomes included incidence, prevalence, death, and DALYs, reported as absolute numbers and age standardized rates (ASRs), as well as ASRs of death and DALYs attributed to risk factors, stratified by sex, age group, year, and SDI quintiles.

To quantify trends in myocarditis burden from 1990 to 2021, we calculated the EAPC using the following formula: Y = *α* + *β*X + *ε*. Here, Y indicates ln(ASR), X represents the calendar year, α refers to the intercept, *β* stands for the annual variation in ln(ASR), and ε denotes the error term. Additionally, EAPC was computed as EAPC = 100 × (e^β − 1), with 95% confidence intervals (CI) obtained from linear regression models (18, 26). The positive EAPC signified an escalating trend, while the negative figure denoted a declining one. Spearman’s rank correlation tests were employed to examine associations between SDI and ASRs in 2021, as well as EAPC in ASRs from 1990 to 2021. These relationships were visualized using Locally Weighted Scatterplot Smoothing curves.

Our analysis covered the number and ASRs of incidence, prevalence, death, and DALYs of myocarditis in both 1990 and 2021, as well as EAPC of these metrics over the period from 1990 to 2021 at global and regional scales. And ASRs were utilized to enable cross-group comparisons of incidence, prevalence, death, and DALYs, taking into account differences in sex, age, year, SDI, and location. Then, we compared the impact of non-optimal temperature on ASDR and ASYR due to myocarditis, once again considering the dimensions of sex, year, age, and SDI. The results were presented using epidemiological maps and trend plots for clear and vivid visualization. Statistical analyses were performed using the R program (version 4.0.3), with significance set at *p* < 0.05.

## Results

### Incidence burden of myocarditis

**Figure 1:**
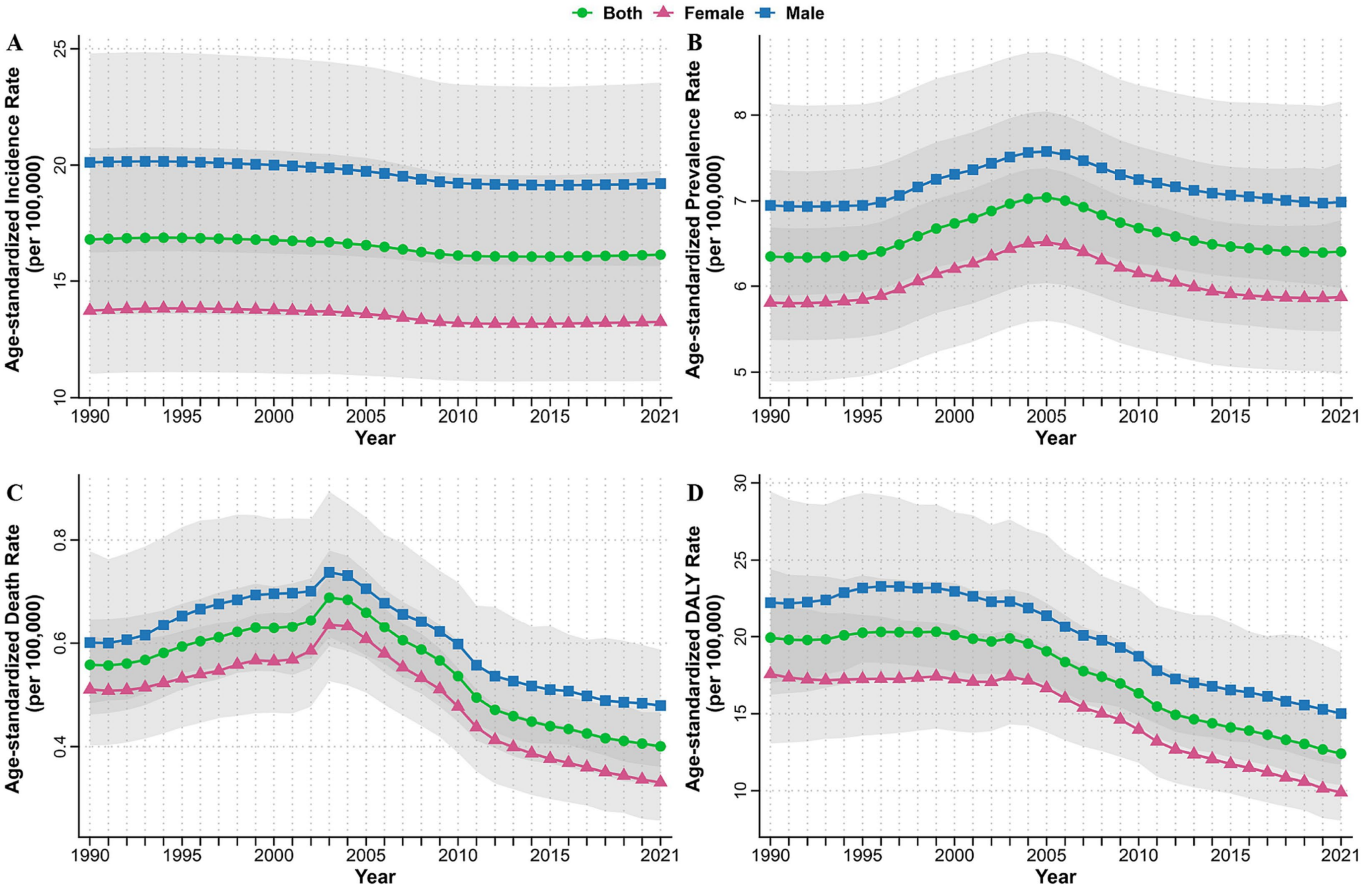
Age-standardized incidence **(A)**, prevalence **(B)**, death **(C)**, and DALYs **(D)** rates of myocarditis from 1990 to 2021 by sex at the global level. DALYs, disability-adjusted life years.

**Figure 2:**
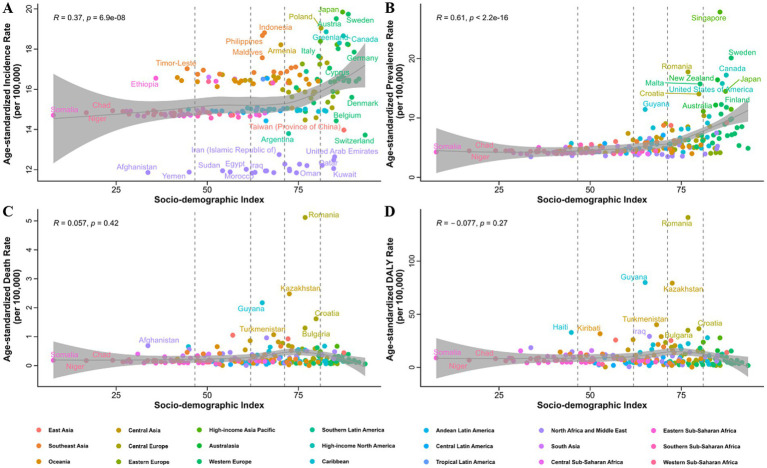
Correlation of the age-standardized incidence **(A)**, prevalence **(B)**, death **(C)**, and DALYs **(D)** rates of myocarditis in 2021 with different SDI in 204 countries and territories. DALYs, disability-adjusted life years; SDI, socio-demographic index.

Globally, the incidence of myocarditis cases increased from 790,794 (95% UI: 638,741 to 976,816) in 1990 to 1,319,704 (1,072,475 to 1,618,748) in 2021. However, the ASIR per 100,000 people was 16.82 (13.53 to 20.72) in 1990 and 16.16 (13.11 to 19.76) in 2021 (Table 1), with a slight decline in EAPC of −0.20 (−0.23 to −0.17) (Table 1). Overall, the ASIR of myocarditis at the global level declined initially from 1990 to 2016, followed by a period of slight upward trend from 2017 to 2021 (Figure 1A). In 2021, among the 21 regions, High-income Asia Pacific [19.36 (15.94 to 23.71)] and High-income North America [18.36 (15.69 to 21.93)] had the highest ASIR of myocarditis per 100,000 people. In contrast, the lowest ASIR was reported in North Africa and Middle East [12.15 (9.71 to 15.06)], followed by the Andean Latin America [14.74 (11.87 to 18.05)] (Table 1). Notably, from 1990 to 2021, Central Asia [0.05 (0.04 to 0.05)] presented the highest increase in EAPC of ASIR [0.05 (0.04 to 0.05)], while the largest decrease occurred in High-income North America [−0.68 (−0.86 to −0.50)] (Table 1). Among the 204 countries and territories, the ASIR of myocarditis ranged from 11.83 to 19.84 per 100,000 population. Japan [19.84 (16.38 to 24.21)] and Sweden [19.74 (16.15 to 24.26)] had the highest rates, while the Syrian Arab Republic [11.83 (9.43 to 14.65)] reported the lowest (Figure 2A; Supplementary Table S4). Between 1990 and 2021, the EAPC in ASIR attributed to myocarditis declined significantly in the United States of America [−0.76 (−0.96 to −0.56)] and Italy [−0.70 (−0.82 to −0.58)] and the largest increase was found in the United Arab Emirates [0.09 (0.06 to 0.12)] (Supplementary Table S2; Supplementary Figure S1A).

**Table 1:** The incidence cases and age-standardized incidence rate of myocarditis in 1990 and 2021, and EAPC in age-standardized incidence rate from 1990 to 2021.

Characteristics	Incidence cases (95% UI)		ASIR (95% UI)		EAPC in ASIR (95% UI)
	1990	2021	1990	2021	1990–2021
Global	790,794 (638,741 to 976,816)	1,319,704 (1,072,475 to 1,618,748)	16.82 (13.53 to 20.72)	16.16 (13.11 to 19.76)	−0.20 (−0.23 to −0.17)
Gender					
Male	461,971 (375,593 to 570,565)	758,457 (616,294 to 927,839)	20.12 (16.25 to 24.80)	19.21 (15.64 to 23.54)	−0.22 (−0.24 to −0.19)
Female	328,823 (262,874 to 405,701)	561,247 (454,060 to 689,151)	13.74 (11.03 to 16.88)	13.25 (10.72 to 16.18)	−0.20 (−0.23 to −0.17)
SDI					
High-middle SDI	170,132 (136,769 to 209,911)	247,020 (199,951 to 303,461)	16.76 (13.53 to 20.64)	15.85 (12.90 to 19.37)	−0.25 (−0.31 to −0.20)
High SDI	178,162 (142,892 to 220,499)	262,048 (214,105 to 320,130)	18.27 (14.80 to 22.51)	17.60 (14.60 to 21.33)	−0.33 (−0.40 to −0.25)
Low-middle SDI	144,112 (115,723 to 179,716)	269,694 (217,931 to 329,995)	15.97 (12.86 to 19.60)	15.84 (12.77 to 19.45)	−0.03 (−0.03 to −0.03)
Low SDI	57,103 (45,317 to 71,346)	129,597 (102,930 to 161,472)	15.52 (12.52 to 18.99)	15.45 (12.47 to 18.93)	−0.01 (−0.02 to −0.01)
Middle SDI	240,529 (193,130 to 298,121)	410,262 (330,745 to 506,024)	16.81 (13.48 to 20.79)	16.33 (13.16 to 20.01)	−0.12 (−0.13 to −0.11)
Region					
East Asia	185,823 (147,455 to 231,736)	277,632 (225,473 to 340,899)	17.32 (13.85 to 21.53)	16.25 (13.24 to 19.93)	−0.28 (−0.32 to −0.23)
Southeast Asia	67,838 (55,006 to 84,385)	120,105 (97,194 to 149,134)	17.83 (14.44 to 21.86)	17.87 (14.46 to 21.90)	0.00 (0.00 to 0.00)
Oceania	841 (671 to 1,043)	1865 (1,508 to 2,308)	16.51 (13.42 to 20.26)	16.52 (13.43 to 20.27)	0.00 (0.00 to 0.01)
Central Asia	9,843 (7,897 to 12,121)	14,800 (11,942 to 18,519)	16.30 (13.09 to 20.11)	16.51 (13.27 to 20.43)	0.05 (0.04 to 0.05)
Central Europe	21,766 (17,492 to 26,781)	25,606 (20,458 to 31,846)	16.58 (13.46 to 20.45)	16.85 (13.68 to 20.72)	−0.02 (−0.05 to 0.02)
Eastern Europe	41,104 (33,071 to 50,545)	43,806 (35,370 to 54,243)	17.01 (13.77 to 20.79)	17.10 (13.83 to 20.88)	0.01 (0.01 to 0.02)
High-income Asia Pacific	36,169 (28,863 to 44,527)	53,322 (42,180 to 64,880)	19.96 (16.24 to 24.48)	19.36 (15.94 to 23.71)	−0.19 (−0.24 to −0.13)
Australasia	3,473 (2,770 to 4,321)	6,434 (5,204 to 7,922)	15.98 (12.81 to 19.76)	15.98 (12.91 to 19.66)	0.01 (−0.02 to 0.03)
Western Europe	79,344 (63,466 to 98,204)	106,835 (85,765 to 129,934)	17.29 (14.15 to 21.18)	16.94 (14.06 to 20.68)	−0.11 (−0.14 to −0.09)
Southern Latin America	6,924 (5,598 to 8,536)	11,436 (9,400 to 13,954)	14.56 (11.74 to 18.03)	14.75 (12.17 to 18.00)	0.04 (0.02 to 0.06)
High-income North America	61,341 (48,647 to 76,701)	89,201 (75,600 to 106,857)	19.33 (15.41 to 24.09)	18.36 (15.69 to 21.93)	−0.68 (−0.86 to −0.50)
Caribbean	4,623 (3,698 to 5,715)	7,598 (6,065 to 9,341)	15.01 (11.97 to 18.42)	14.99 (11.96 to 18.39)	0.00 (0.00 to 0.00)
Andean Latin America	4,319 (3,505 to 5,310)	9,274 (7,502 to 11,307)	14.81 (11.95 to 18.14)	14.74 (11.87 to 18.05)	−0.01 (−0.01 to 0.00)
Central Latin America	19,828 (15,785 to 24,703)	39,713 (31,709 to 48,690)	15.82 (12.68 to 19.38)	15.75 (12.62 to 19.29)	−0.01 (−0.02 to −0.01)
Tropical Latin America	20,344 (16,316 to 25,285)	39,725 (31,951 to 49,552)	16.50 (13.23 to 20.32)	16.46 (13.19 to 20.27)	−0.01 (−0.01 to −0.01)
North Africa and Middle East	31,832 (25,155 to 40,088)	67,470 (53,715 to 84,599)	12.15 (9.72 to 15.05)	12.15 (9.71 to 15.06)	0.00 (0.00 to 0.01)
South Asia	139,706 (112,182 to 174,821)	275,335 (222,416 to 335,892)	16.44 (13.25 to 20.09)	16.34 (13.17 to 19.98)	−0.02 (−0.02 to −0.02)
Central Sub-Saharan Africa	5,810 (4,631 to 7,250)	14,704 (11,582 to 18,361)	14.81 (12.00 to 18.11)	14.75 (11.93 to 18.06)	−0.01 (−0.02 to −0.01)
Eastern Sub-Saharan Africa	20,957 (16,555 to 26,278)	48,058 (37,819 to 60,235)	15.46 (12.50 to 18.89)	15.44 (12.46 to 18.88)	−0.01 (−0.01 to −0.01)
Southern Sub-Saharan Africa	6,517 (5,254 to 8,127)	11,209 (9,074 to 13,692)	15.90 (12.82 to 19.46)	15.90 (12.83 to 19.45)	0.01 (0.00 to 0.01)
Western Sub-Saharan Africa	22,394 (17,829 to 27,917)	55,576 (43,840 to 69,549)	15.69 (12.66 to 19.19)	15.56 (12.55 to 19.03)	−0.03 (−0.03 to −0.02)

EAPC, estimated annual percentage changes; UI, uncertainty intervals; ASIR, age-standardized incidence rate.

### Prevalence burden of myocarditis

At the global level, the number of prevalent myocarditis cases witnessed an increase, rising from 320,623 (268,557 to 371,912) in 1990 to 505,030 (432,295 to 587,819) in 2021. The ASPR per 100,000 people also exhibited a minor uptick (no statistical significance), climbing from 6.35 (5.37 to 7.36) in 1990 to 6.41 (5.48 to 7.44) in 2021 (Supplementary Table S3). Specifically, there was an upward trajectory from 1990 to 2005, followed by a decline from 2006 to 2021 in global ASPR (Figure 1B). At the regional level in 2021, the ASPR of myocarditis per 100,000 people was highest in High-income North America [15.94 (13.05 to 19.33)] and High-income Asia Pacific [14.13 (11.61 to 17.00)], while it was the lowest in Central Sub-Saharan Africa [4.02 (3.32 to 4.88)] and Andean Latin America [4.10 (3.42 to 4.89)] (Supplementary Table S3). Concurrently, High-income Asia Pacific [1.28 (0.97 to 1.59)] registered the most substantial increment, whereas Eastern Sub-Saharan Africa [−0.77 (−0.82 to −0.71)] endured the most significant reduction in the EAPC of ASPR from 1990 to 2021 (Supplementary Table S3). Among the 204 country levels, Singapore [27.84 (22.49 to 34.72)] boasted the highest ASPR per 100,000 for myocarditis, with Sweden followed closely [20.11 (16.76 to 23.71)]. The lowest ASPR was identified in Afghanistan [3.46 (2.88 to 4.11)] and Türkiye [3.48 (3.48 to 4.11)] (Figure 2B; Supplementary Table S4). Regarding the EAPC in ASPR from 1990 to 2021, Ireland [3.37 (2.30 to 4.46)] and Malta [3.16 (1.89 to 4.45)] showed the rapid growth, while Italy [−2.08 (−3.10 to −1.04)] showed the largest decline (Supplementary Table S4; Supplementary Figure S1B).

### Death burden of myocarditis

In 2021, myocarditis was accountable for 31,765 (25,490 to 37,053) global deaths, representing a slight escalation from 21,765 (17,945 to 25,869) deaths recorded in 1990. The global ASDR for myocarditis per 100,000 people stood at 0.56 (0.46 to 0.65) in 1990. It manifested a gradual ascent from 1990 to 2003, followed by a sustained descent from 2003 to 2021, reaching 0.40 (0.32 to 0.47) in 2021 (Figure 1C; Supplementary Table S5). Among the 21 GBD regions, Central Europe [1.24 (0.99 to 1.52)] and East Asia [0.91 (0.63 to 1.18)] disclosed the highest ASDR of myocarditis per 100,000 population. By contrast, Southern Latin America [0.10 (0.09 to 0.12)] and Andean Latin America [0.06 (0.04 to 0.08)] presented the lowest ASDR in 2021 (Supplementary Table S5). Additionally, Central Latin America [2.23 (1.94 to 2.51)] and Central Asia [2.86 (2.10 to 3.64)] experienced the most pronounced increments in ASDR from 1990 to 2021. Conversely, the most substantial reduction in ASDR was noted in Andean Latin America [−4.74 (−5.02 to −4.46)] and Western Europe [−4.30 (−5.63 to −2.95)] (Supplementary Table S5). Among the 204 countries and territories, the ASDR for myocarditis ranged from 0.00 to 5.12 per 100,000 people in 2021. The highest ASDR was observed in Romania [5.12 (3.90 to 6.59)], Kazakhstan [2.48 (1.83 to 3.31)] and Guyana [2.17 (1.63 to 2.74)] (Figure 2C; Supplementary Table S6). Kazakhstan [13.64 (11.37 to 15.95)] also displayed the greatest increase in ASDR from 1990 to 2021, followed by Mexico [5.34 (4.75 to 5.94)] and Ireland [5.20 (3.12 to 7.33)]. The most significant decrease in ASDR was detected in Italy [−8.36 (−10.10 to −6.59)], Sri Lanka [−6.73 (−7.63 to −5.82)], Peru [−5.54 (−5.76 to −5.33)] and Cyprus [−5.24 (−5.74 to −4.73)] (Supplementary Table S6; Supplementary Figure S1C).

### The DALYs burden of myocarditis

Globally, the estimated DALYs attributed to myocarditis slightly declined from approximately 1,032,803 (829,855 to 1,303,917) in 1990 to 963,139 (795,811 to 1,148,366) in 2021. Concurrently, the global ASYR per 100,000 people showed a generally steady decrease from 19.93 (16.23 to 24.41) in 1990 to 12.41 (10.37 to 14.76) in 2021 (Figure 1D; Supplementary Table S7). In 2021, Central Europe [34.11 (26.56 to 42.43)] reported the highest ASYR per 100,000 people for myocarditis, followed by Central Asia [27.41 (21.93 to 34.56)] and East Asia [25.02 (17.86 to 30.91)]. Conversely, the lowest ASYR were observed in Andean Latin America [2.12 (1.56 to 2.71)] and Southern Latin America [3.67 (3.32 to 4.07)] (Supplementary Table S7). Notably, Andean Latin America [−4.45 (−4.73 to −4.17)] and Southern Latin America [−3.60 (−4.03 to −3.18)] were also the regions with the largest declines in global ASYR, whereas Central Asia [2.61 (1.78 to 3.44)] showed the most significant increase from 1990 to 2021(Supplementary Table S7). The national ASYR for myocarditis in 2021 varied widely, ranging from 0.31 to 141.03 per 100,000 people. The countries and territories with the highest ASYR in 2021, as well as those showing the greatest increase or decrease in EAPC of ASYR from 1990 to 2021, can be detected in Figure 2D, Supplementary Table S8, and Supplementary Figure S1D.

### Age- and sex-related burden of myocarditis

**Figure 3:**
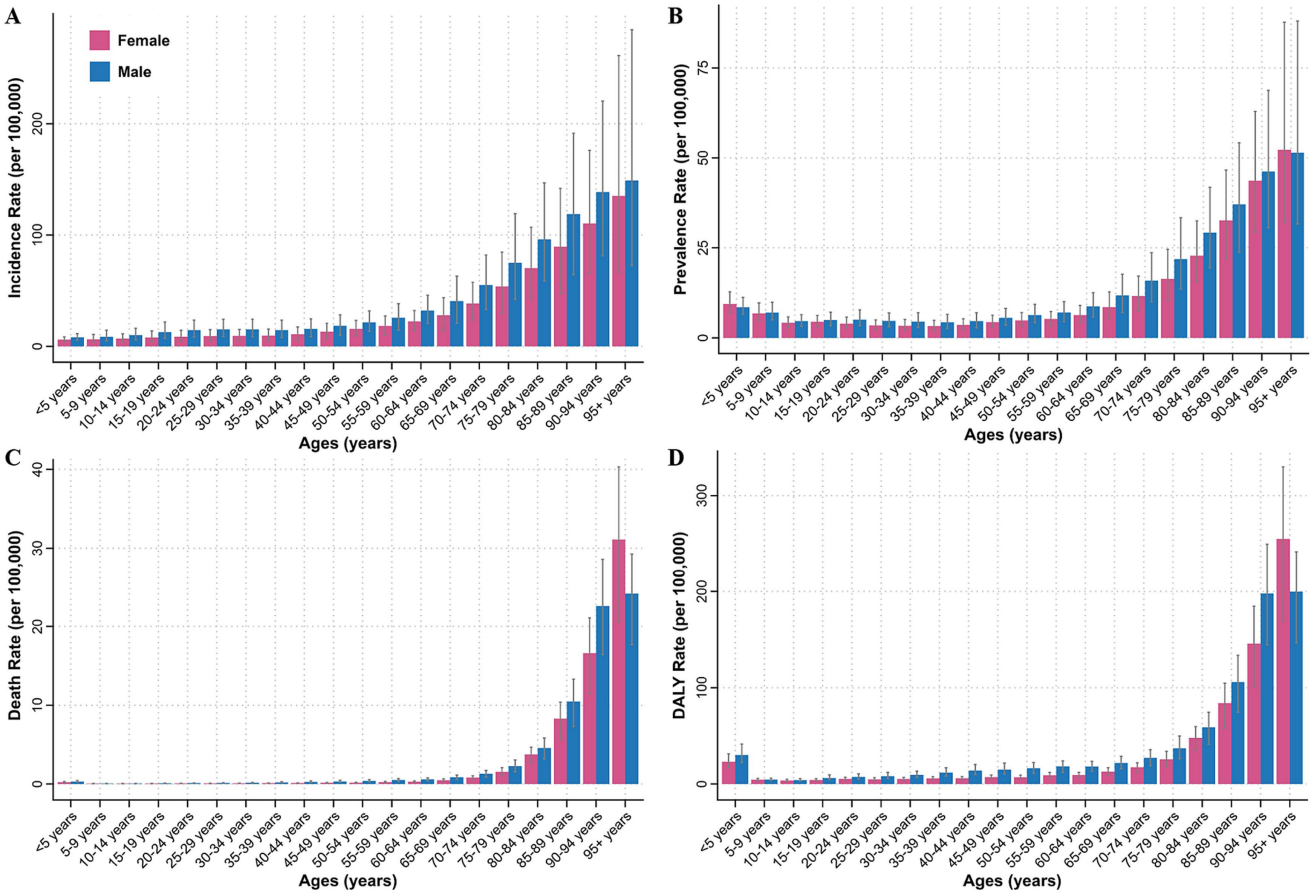
Incidence **(A)**, prevalence **(B)**, death **(C)**, and DALYs **(D)** rates of myocarditis by age and sex in 2021 at the global level. DALYs, disability-adjusted life years.

From 1990 to 2021, the global trends in ASIR, ASPR, ASDR and ASYR per 100,000 people for myocarditis exhibited consistent patterns by sex, with males carrying a higher burden than females (Figures 1A–D). Subsequently, we carried out a detailed analysis of the age and sex disparities in incidence, prevalence, death, and DALY rates per 100,000 people in 2021. The results showed that the incidence rate gradually increased with advancing age, reaching a peak at 95 years or above. Consistently, the incidence rate was higher in men than in female (Figure 3A). For the prevalence rate, it remained relatively stable for both males and females between the ages of 10 to 60 years, However, it was higher at ages younger than 10 years and increased gradually after 60 years. Notably, the prevalence rate was higher in females than in males at ages younger than 5 years and older than 95 years in 2021, while males demonstrated a higher prevalence rate at other ages (Figure 3B). With the exception of children under 5 years of age, both the death and DALYs rates increased with age. Surprisingly, at ages exceeding 95 years, the rates were higher in females than in males (Figures 3C,D). To delve into the impact of age and sex on the burden imposed by myocarditis over time, we classified individuals into two groups: those younger than 70 years and those older than 70 years. Our analysis unveiled that the burden in terms of incidence, prevalence, death, and DALYs rate consistently remained higher in males compared to that in females from 1990 to 2021. Concurrently, individuals aged over 70 years exhibited higher incidence, prevalence, death, and DALY rates than those younger than 70 years throughout the period from 1990 to 2021 (Supplementary Figure S2).

### The burden of myocarditis by SDI

**Figure 4:**
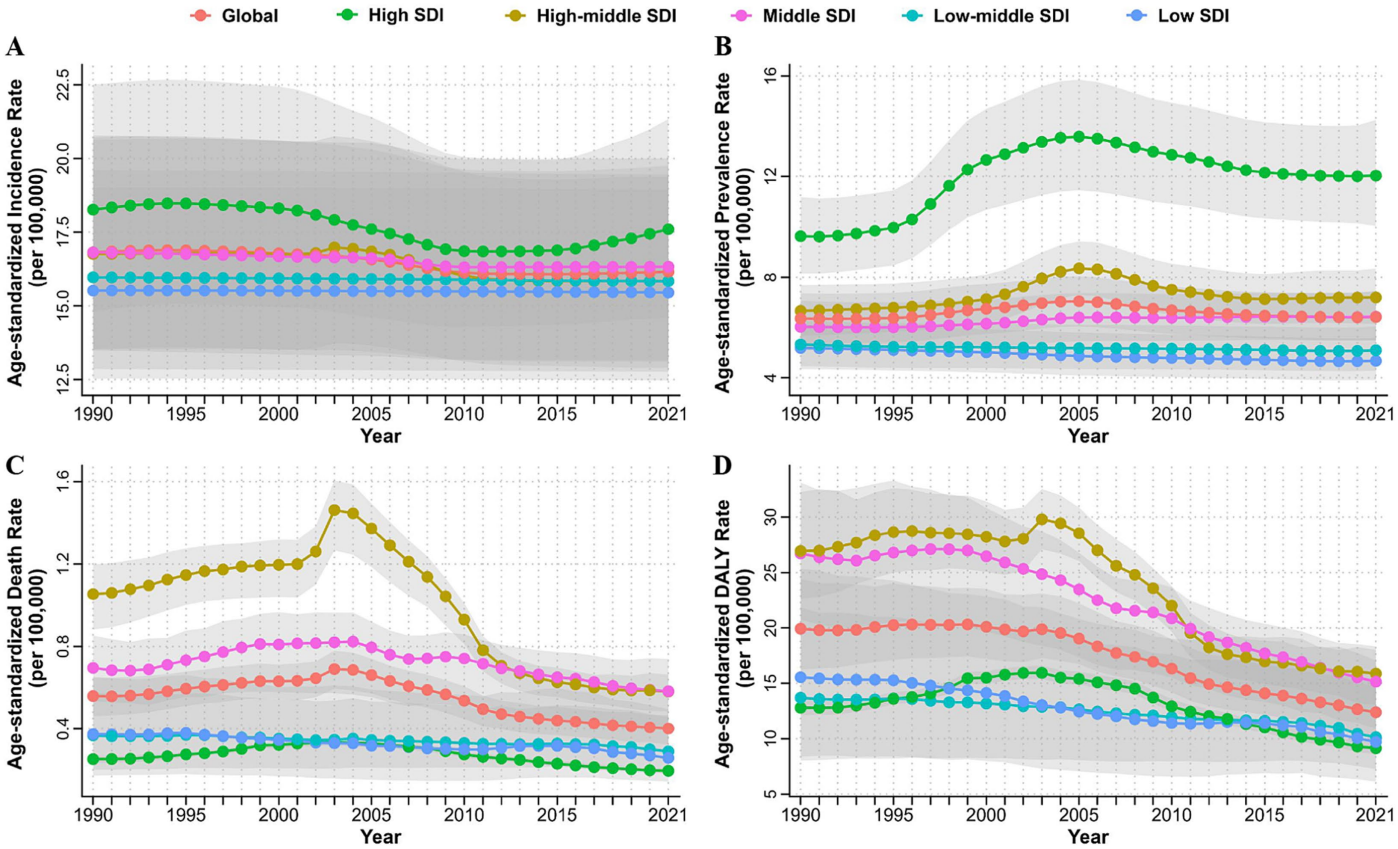
Age-standardized incidence **(A)**, prevalence **(B)**, death **(C)** and DALYs **(D)** rates of myocarditis from 1990 to 2021 by SDI quintile. DALYs, disability-adjusted life years; SDI, socio-demographic index.

The burden of myocarditis varied significantly across different SDI quintiles. From 1990 to 2021, the high SDI quintile observed the largest decrease in EAPC for ASIR [−0.33 (−0.40 to −0.25)] and largest increase in EAPC for ASPR [0.73 (0.36 to 1.10)] (Figure 4A; Table 1; Supplementary Table S3). At the same time, it consistently displayed the highest ASIR and ASPR, while the low SDI quintile exhibited the lowest rates during the period from 1990 to 2021 (Figures 4A,B; Table 1; Supplementary Table S3). The high-middle SDI quintile experienced the most significant decreases in EAPC for ASDR [−2.59 (−3.36 to −1.81)] and ASYR [−2.22 (−2.66 to −1.79)] from 1990 to 2021 (Figures 4C,D; Supplementary Tables S5, S7). The highest ASDR and ASYR were recorded in the high-middle and middle SDI quintiles (Figures 4C,D). In addition, a positive correlation was found between SDI and ASIR (R = 0.37, *p* < 0.05) and ASPR (R = 0.61, *p* < 0.05) of myocarditis in 2021 (Figures 2A,B). However, there was no significant correlation between SDI and ASDR (R = 0.057, *p* = 0.42) and ASYR (R = -0.077, *p* = 0.27) (Figures 2C,D). Further analysis of changes from 1990 to 2021 revealed a positive association between SDI and EAPC in ASIR (R = 0.19, *p* < 0.05), ASPR (R = 0.49, *p* < 0.05), ASDR (R = 0.31, *p* < 0.05), and ASYR (R = 0.28, *p* < 0.05) of myocarditis (Supplementary Figures S1A–D).

### Risk factors of myocarditis

**Figure 5:**
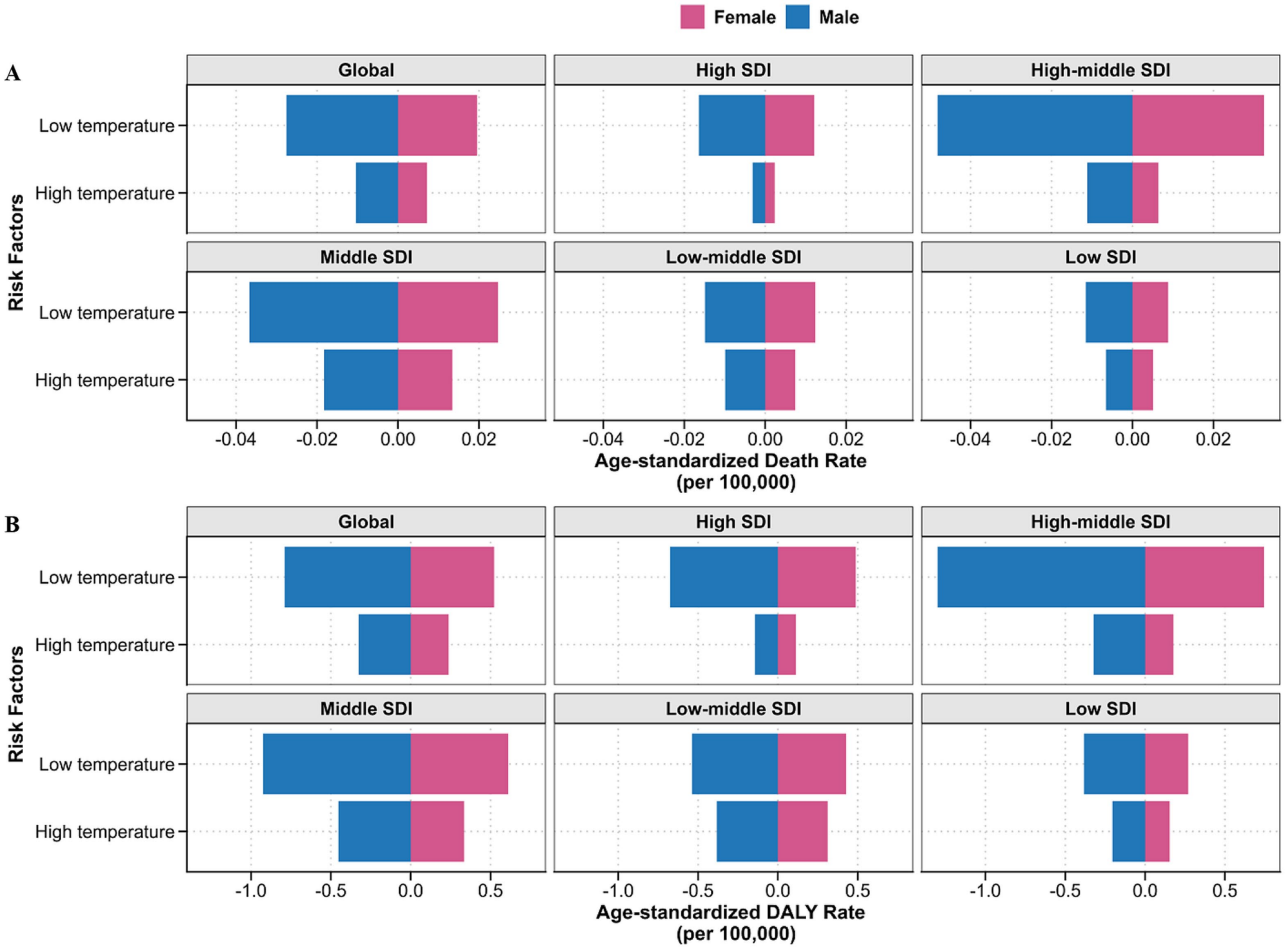
Age-standardized death **(A)** and DALYs **(B)** rates of myocarditis attributed to non-optimal temperature by SDI quintile and sex in 2021. Horizontal bars represent sex-stratified burden estimates, with male displayed to the left of the zero axis and female to the right. The absolute values along the X-axis represent the extent of myocarditis burden attributed to non-optimal temperatures for each sex, without indicating the direction of risk. DALYs, disability-adjusted life years; SDI, socio-demographic index.

GBD 2021 evaluated the impact of non-optimal temperature as a risk factor for myocarditis-related ASDR and ASYR. From 1990 to 2021, although there was a decreasing trend in myocarditis-related ASDR and ASYR attributed to low temperature, low temperature had a more significant effect on myocarditis-related burden compared to high temperature (Supplementary Figure S3). Furthermore, non-optimal temperature exhibited age- and sex-specific effects, with males and the older adult experiencing higher myocarditis-related ASDR and ASYR due to non-optimal temperature (Figure 5; Supplementary Figures S3, S4).

The influence of non-optimal temperature on myocarditis-related ASDR and ASYR varied across SDI quintiles. Despite a noteworthy decrease in ASDR and ASYR due to low temperature in the high-middle quintile, the high-middle quintile still had the most significant myocarditis-related burden related to low temperature for myocarditis. No significant trend could be observed in the impact of high temperature on ASDR and ASYR over the years within each SDI quintile. However, it exerted the greatest impact in the middle SDI quintile (Figure 5; Supplementary Figure S5). Subsequently, we investigated the impact of non-optimal temperature in 21 GBD regions. The results indicated that high temperature notably affected myocarditis-related ASDR and ASYR in East Asia, North Africa and the Middle East, Central Asia, and South Asia. In contrast, low temperature mainly influenced in Central Europe, Central Asia and East Asia (Supplementary Figure S6).

## Discussion

Our study provides a comprehensive and systematic assessment of the global, regional, and national burden of myocarditis from 1990 to 2021. During this period, the global incidence of myocarditis increased from 790,794 cases in 1990 to 1,319,704 cases in 2021, However, the ASIR per 100,000 people slightly decreased from 16.82 in 1990 to 16.16 in 2021. This trend reflects significant population growth, advancements in sensitive screening tools, and progress in preventive measures (27). Additionally, the widespread implementation of sensitive screening tools for early-stage detection, improvements in treatment leading to enhanced patient survival, population aging increasing the proportion of the older adult susceptible to myocarditis, and changes in the natural course of myocarditis causing the accumulation of patients (28) might explain the slight increase in the ASPR of myocarditis from 6.35 per 100,000 people in 1990 to 6.41 in 2021 per 100,000 people. Since 2019, the rapid global spread of SARS-CoV-2 and its resultant COVID-19 has been noteworthy (29). Among hospitalized patients with COVID-19, the occurrence of acute myocarditis was reported between 2.4 and 4.1 cases per 1,000 patients (30). Our study has, for the first time, filled in the gap regarding the disease burden of myocarditis following the outbreak of COVID-19, indicating an increased ASIR from 2019 to 2021. Overall, the burden of myocarditis remains high globally. Therefore, enhancing the establishment of a global myocarditis surveillance system and promoting research on prevention and treatment are critical priorities. Concurrently, formulating universal diagnosis and treatment guidelines, disseminating them globally, and implementing health education campaigns to improve public awareness will be essential for future disease management.

The clinical presentation of myocarditis can serve as a predictor of patient outcomes. Specifically, the presence of reduced left ventricular ejection fraction, worsening heart failure, sustained ventricular arrhythmias, and atrioventricular block has been associated with an increased risk of mortality in patients with myocarditis (31). Previous epidemiological investigations estimating the global burden of myocarditis indicated that the ASDR showed decreasing trends from 1990 to 2019 (22). Our research, using the updated GBD 2021 database, observed consistent results extending from 1990 to 2021. This decline might be attributed to enhanced screening sensitivity and clinical management of myocarditis. On one hand, the advent of sensitive screening tools provided non-invasive tissue characterization of the myocardium, facilitating the early identification of suspected cases (32, 33). On the other hand, the adoption of ventricular assist devices or extracorporeal membrane oxygenation in patients with hemodynamically unstable heart failure (34, 35), and the growing application of immunomodulatory therapies might contribute to the gradual reduction in mortality burden from myocarditis (3, 36).

DALYs encompass both years of life lost and years lived with disability, serving as a composite measure to quantify both fatal and non-fatal health losses, thus reflecting the overall impact of diseases on health (18). About 50% of acute myocarditis cases resolved rapidly within the initial 24–48 h; however, approximately 25% of cases developed persistent cardiac dysfunction. Additionally, 12–25% of cases acutely deteriorated, leading to death or progression to end-stage dilated cardiomyopathy, necessitating recurrent hospitalizations and heart transplantation (37–39). In our study, we observed 963,139 DALYs in 2021, despite a notable decline in the ASYR from 19.93 in 1990 to 12.41 in 2021 [EAPC in ASYR: −1.71(−1.95 to −1.46)]. This decline in ASYR is more pronounced than the reductions in the ASIR and ASDR, likely due to an increase in early diagnoses facilitated by cardiovascular magnetic resonance imaging.

The SDI serves as a comprehensive indicator used to measure the social development level of countries or regions. Our study revealed a positive correlation between SDI and both the ASIR and ASPR of myocarditis in 2021. This indicated that the burden of myocarditis was generally higher in countries with higher socio-economic development levels. Over the period from 1990 to 2021, the ASIR and ASPR in high SDI quintile consistently remained significantly higher than those in other quintiles. This phenomenon is complex, and can be partially attributed to superior healthcare infrastructure and medical resources, population aging, the introduction of highly sensitive troponin and cardiovascular magnetic resonance imaging examinations, as well as the extensive use of immune checkpoint inhibitors and vaccines (31). Hence, for high-SDI countries, pharmacovigilance systems should be strengthened to actively monitor myocarditis risks linked to immunotherapies and vaccines, particularly as novel therapies are introduced.

Although a population-based cohort study revealed that individuals with low education had 1.66-fold higher risk of cardiovascular mortality than those with high education (40), our study found no statistically significant relationship between SDI and either the ASDR or ASYR. Our observations indicated that despite the declining ASDR and ASYR in the high-middle and middle SDI quintiles, these regions still faced the heaviest disease burden in terms of ASDR and ASYR. Specifically, countries such as Romania, Kazakhstan, and Guyana reported the highest levels of ASDR and ASYR. The key factors contributing to this may include delayed diagnosis, incomplete healthcare systems and insufficient medical resources in the high-middle and middle SDI quintiles. Finally, the low SDI quintile having the lowest ASR may reflect a shortage of healthcare infrastructure and resources, resulting in many cases going unreported. These inter-regional disparities indicated that public health organizations should implement region-targeted measures to gradually alleviate the disease burden of myocarditis.

In line with previous GBD epidemiological study (22), our research revealed a sex-specific tendency in the burden of myocarditis, Generally, males experienced a significantly higher disease burden than females from 1990 to 2021. Additionally, although the ASIR, ASDR and ASYR decreased for both sexes from 1990 to 2021, the decline was more notable in females than that in males. Sex-specific interventions, such as male-focused awareness campaigns promoting early symptom recognition and timely healthcare engagement, are critical to address the disproportionate burden among males. Sex hormones play an important role in this phenomenon (41). Experimental research on coxsackievirus-induced myocarditis observed that testosterone enhanced the binding of the virus to cardiomyocytes, induced a Th1-type immune response, suppressed anti-inflammatory cells, and upregulated genes associated with cardiac fibrosis and remodeling. In contrast, estrogen exerted a protective effect in myocarditis by promoting a Th2-type immune response and inhibiting proinflammatory T cells (41–43). Apart from the influence of sex hormones, lifestyle factors such as smoking, drinking, exercise habits might also contribute to the sex differences in the burden of myocarditis. However, the precise mechanisms underlying these differences require further investigation.

The burden of myocarditis demonstrated distinct variations across different age groups in 2021. Generally, the disease burden rose steadily with advancing age. Specifically, the incidence, prevalence, death, and DALYs rates of myocarditis increased sharply after the age of 70, peaking among those aged 95 years and older. This trend highlighted the significant burden of myocarditis in the older adult population, potentially exacerbated by accelerated population aging, dysfunction of the aging immune system, and an increased risk of accumulating chronic myocarditis (44–46). integrating standardized cardiac screenings into routine geriatric care protocols for older adult patients presenting with nonspecific symptoms could mitigate age-related disparities. Notably, although the incidence rate was relatively low among children under 5 years old, the prevalence and DALYs rates were high in this age group. This phenomenon likely stems from the immature immune systems of younger children, making them more vulnerable to viral infections that cause myocarditis and result in poor prognosis. A study on pediatric acute myocarditis in the United States disclosed that approximately 80.4% of patients were admitted to the intensive care unit, 44.4% required mechanical ventilation, and 23.2% needed mechanical circulatory support. Moreover, myocardial recovery was observed in only 59.6% of the patients receiving extracorporeal membrane oxygenation or ventricular assist device, but 16.3% were successfully bridged to transplantation (47). Unfortunately, the prognosis was even worse for fulminant myocarditis. A survey in Japan indicated that the mortality rate could reach as high as 48.4% in children (48). These findings emphasized the necessity of targeted interventions to address the unique challenges posed by myocarditis across different age groups.

A previous GBD study focusing on the risk factors of cardiomyopathy and myocarditis among individuals aged 60 to 89 years identified high systolic blood pressure and alcohol consumption as the principal risk factors for mortality related to these disorders [20]. Nevertheless, specific risk factor analyses for myocarditis remains insufficiently explored. Our study evaluated the risk factors attributing to the burden of myocarditis for the first time. We demonstrated that both high and low temperatures significantly influenced the ASDR and ASYR of myocarditis. Research on the impact of temperature on cardiovascular disease also identified a J-shaped relationship between non-optimal temperature with mortality (49), a pattern potentially applicable to myocarditis as well. Our research further discovered that, compared to high temperature, low temperature had a more notable impact on myocarditis, especially in high-middle SDI quintile. Cold temperature can induce peripheral vasoconstriction to reduce blood flow and heat loss. Concurrently, vasoconstriction increases blood pressure and cardiac workload, potentially triggering cardiovascular events (50, 51). These findings highlighted the interactions between temperature and myocarditis. However, the specific role of low temperature in the burden of myocarditis remains unclear and requires further investigation. Although the impact of high temperature on the ASDR and ASYR of myocarditis were stable from 1990 to 2021, with the greatest burden observed in the middle SDI quintile, it also warrants attention due to the ongoing global warming trend (43). Additionally, heavy alcohol consumption alters both lymphocyte and granulocyte production and function, impairs the immune system, and leads to a poor prognosis in myocarditis patients (52). However, due to limitations inherent in GBD data, comprehensive future research is needed to explore broader risk factors contributing to myocarditis.

Several limitations merit consideration. Firstly, this study, like other studies relying on GBD data, collected data from multiple sources with inconsistence in both quality and quantity. In low-income countries, limited access to advanced diagnostics leads to systematic underreporting, likely underestimating the true incidence and mortality rates. Such data incompleteness challenges the generalizability of global estimates. Although robust statistical methods, such as DisMod-MR 2.1, were applied to address these issues, it was still impossible to precisely predict the burden of myocarditis for all regions and populations (18). Future efforts should focus on acquiring representative data from more countries through comprehensive health surveys. Secondly, with the introduction of high-sensitivity cardiac troponin tests and cardiac magnetic resonance imaging, the clinical diagnosis of myocarditis has shifted from endomyocardial biopsy to a comprehensive assessment integrating symptoms, signs, laboratory tests, and imaging results (31). This evolution in diagnostic strategy raises questions regarding the consistency of disease reporting standards across different periods. Finally, etiological aggregation obscures cause-specific burden patterns. Although myocarditis pathogenesis varies markedly by etiology, the GBD framework combines all subtypes due to sparse cause-specific data. Establishing global registries stratified by etiology should be prioritized to inform targeted interventions.

## Conclusion

Our study offered a comprehensive analysis of the global, regional, and national burden of myocarditis, highlighting significant disparities by sex, age, region, and SDI over the past three decades. The highest ASIR and ASPR were observed in the high SDI quintile, while the highest ASDR and ASYR emerged in the middle and high-middle SDI quintiles from 1990 to 2021. Additionally, a high burden of myocarditis was noted among males and the older adult populations. Furthermore, non-optimal temperature, particularly low temperature, was first identified as a significant risk factor for myocarditis-related ASDR and ASYR. This study provided an updated reference for developing and optimizing targeted public health strategies for myocarditis based on sex, age, region, year, and SDI. Future endeavors should focus on research and improvements in prevention, early diagnosis, and treatment measures for different populations and regions to alleviate the health burden associated with myocarditis.

## Data Availability

The datasets presented in this study can be found in online repositories. The names of the repository/repositories and accession number(s) can be found at: https://ghdx.healthdata.org/.
