# Supplementary material for: Global, regional, and national burden of myocarditis and its attributable risk factors in 204 countries and territories from 1990 to 2021: updated systematic analysis
Source: Front Public Health. 2025 Apr 28;13:1542921. doi: 10.3389/fpubh.2025.1542921 (PMC12066271; doi:10.3389/fpubh.2025.1542921)
Supplement: Supplementary file 1 [file Table_1.docx]

# Table S1. Geographic regions, countries or territories in GBD 2021

| **Geographic regions** | **Countries or territories** |
| --- | --- |
| **Central Asia** |  |
|  | Armenia |
|  | Azerbaijan |
|  | Georgia |
|  | Kazakhstan |
|  | Kyrgyzstan |
|  | Mongolia |
|  | Tajikistan |
|  | Turkmenistan |
|  | Uzbekistan |
| **Central Europe** |  |
|  | Albania |
|  | Bosnia and Herzegovina |
|  | Bulgaria |
|  | Croatia |
|  | Czechia |
|  | Hungary |
|  | Montenegro |
|  | North Macedonia |
|  | Poland |
|  | Romania |
|  | Serbia |
|  | Slovakia |
|  | Slovenia |
| **Eastern Europe** |  |
|  | Belarus |
|  | Estonia |
|  | Latvia |
|  | Lithuania |
|  | Republic of Moldova |
|  | Russian Federation |
|  | Ukraine |
| **Australasia** |  |
|  | Australia |
|  | New Zealand |
| **High-income Asia Pacific** |  |
|  | Brunei Darussalam |
|  | Japan |
|  | Republic of Korea |
|  | Singapore |
| **High-income North America** |  |
|  | Canada |
|  | Greenland |
|  | United States of America |
| **Southern Latin America** |  |
|  | Argentina |
|  | Chile |
|  | Uruguay |
| **Western Europe** |  |
|  | Andorra |
|  | Austria |
|  | Belgium |
|  | Cyprus |
|  | Denmark |
|  | Finland |
|  | France |
|  | Germany |
|  | Greece |
|  | Iceland |
|  | Ireland |
|  | Israel |
|  | Italy |
|  | Luxembourg |
|  | Malta |
|  | Monaco |
|  | Netherlands |
|  | Norway |
|  | Portugal |
|  | San Marino |
|  | Spain |
|  | Sweden |
|  | Switzerland |
|  | United Kingdom |
| **Andean Latin America** |  |
|  | Bolivia (Plurinational State of) |
|  | Ecuador |
|  | Peru |
| **Caribbean** |  |
|  | Antigua and Barbuda |
|  | Bahamas |
|  | Barbados |
|  | Belize |
|  | Bermuda |
|  | Cuba |
|  | Dominica |
|  | Dominican Republic |
|  | Grenada |
|  | Guyana |
|  | Haiti |
|  | Jamaica |
|  | Puerto Rico |
|  | Saint Kitts and Nevis |
|  | Saint Lucia |
|  | Saint Vincent and the Grenadines |
|  | Suriname |
|  | Trinidad and Tobago |
|  | United States Virgin Islands |
| **Central Latin America** |  |
|  | Colombia |
|  | Costa Rica |
|  | El Salvador |
|  | Guatemala |
|  | Honduras |
|  | Mexico |
|  | Nicaragua |
|  | Panama |
|  | Venezuela (Bolivarian Republic of) |
| **Tropical Latin America** |  |
|  | Brazil |
|  | Paraguay |
| **North Africa and Middle East** |  |
|  | Afghanistan |
|  | Algeria |
|  | Bahrain |
|  | Egypt |
|  | Iran (Islamic Republic of) |
|  | Iraq |
|  | Jordan |
|  | Kuwait |
|  | Lebanon |
|  | Libya |
|  | Morocco |
|  | Oman |
|  | Palestine |
|  | Qatar |
|  | Saudi Arabia |
|  | Sudan |
|  | Syrian Arab Republic |
|  | Tunisia |
|  | Türkiye |
|  | United Arab Emirates |
|  | Yemen |
| **South Asia** |  |
|  | Bangladesh |
|  | Bhutan |
|  | India |
|  | Nepal |
|  | Pakistan |
| **East Asia** |  |
|  | China |
|  | Democratic People's Republic of Korea |
|  | Taiwan (Province of China) |
| **Oceania** |  |
|  | American Samoa |
|  | Cook Islands |
|  | Fiji |
|  | Guam |
|  | Kiribati |
|  | Marshall Islands |
|  | Micronesia (Federated States of) |
|  | Nauru |
|  | Niue |
|  | Northern Mariana Islands |
|  | Palau |
|  | Papua New Guinea |
|  | Samoa |
|  | Solomon Islands |
|  | Tokelau |
|  | Tonga |
|  | Tuvalu |
|  | Vanuatu |
| **Southeast Asia** |  |
|  | Cambodia |
|  | Indonesia |
|  | Lao People's Democratic Republic |
|  | Malaysia |
|  | Maldives |
|  | Mauritius |
|  | Myanmar |
|  | Philippines |
|  | Seychelles |
|  | Sri Lanka |
|  | Thailand |
|  | Timor-Leste |
|  | Viet Nam |
| **Central Sub-Saharan Africa** |  |
|  | Angola |
|  | Central African Republic |
|  | Congo |
|  | Democratic Republic of the Congo |
|  | Equatorial Guinea |
|  | Gabon |
| **Eastern Sub-Saharan Africa** |  |
|  | Burundi |
|  | Comoros |
|  | Djibouti |
|  | Eritrea |
|  | Ethiopia |
|  | Kenya |
|  | Madagascar |
|  | Malawi |
|  | Mozambique |
|  | Rwanda |
|  | Somalia |
|  | South Sudan |
|  | Uganda |
|  | United Republic of Tanzania |
|  | Zambia |
| **Southern Sub-Saharan Africa** |  |
|  | Botswana |
|  | Eswatini |
|  | Lesotho |
|  | Namibia |
|  | South Africa |
|  | Zimbabwe |
| **Western Sub-Saharan Africa** |  |
|  | Benin |
|  | Burkina Faso |
|  | Cabo Verde |
|  | Cameroon |
|  | Chad |
|  | Côte d'Ivoire |
|  | Gambia |
|  | Ghana |
|  | Guinea |
|  | Guinea-Bissau |
|  | Liberia |
|  | Mali |
|  | Mauritania |
|  | Niger |
|  | Nigeria |
|  | Sao Tome and Principe |
|  | Senegal |
|  | Sierra Leone |
|  | Togo |

# Table S2. The incidence cases and age-standardized incidence rate of myocarditis in 1990 and 2021, and EAPC in age-standardized incidence rate from 1990 to 2021 in 204 countries and territories

| **Characteristics** | **Incidence cases (95% UI)** | | **ASIR (95% UI)** | | **EAPC in ASIR**  **(95% CI)** |
| --- | --- | --- | --- | --- | --- |
|  | **1990** | **2021** | **1990** | **2021** | **1990-2021** |
| Afghanistan | 963 (762 to 1208) | 2609 (2008 to 3337) | 11.83 (9.44 to 14.67) | 11.86 (9.48 to 14.71) | 0.01 (0.01 to 0.01) |
| Albania | 453 (362 to 558) | 520 (415 to 645) | 15.91 (12.86 to 19.56) | 15.98 (12.93 to 19.63) | 0.03 (0.01 to 0.04) |
| Algeria | 2302 (1814 to 2926) | 4821 (3861 to 6053) | 11.93 (9.54 to 14.78) | 11.94 (9.54 to 14.79) | 0.01 (0.00 to 0.01) |
| American Samoa | 6 (5 to 8) | 8 (6 to 10) | 16.42 (13.33 to 20.13) | 16.42 (13.34 to 20.16) | 0.00 (-0.01 to 0.00) |
| Andorra | 9 (7 to 12) | 19 (15 to 23) | 16.75 (13.68 to 20.68) | 16.63 (13.60 to 20.53) | -0.04 (-0.04 to -0.03) |
| Angola | 1077 (857 to 1345) | 3388 (2670 to 4250) | 14.87 (12.04 to 18.18) | 14.71 (11.91 to 18.00) | -0.03 (-0.04 to -0.03) |
| Antigua and Barbuda | 9 (7 to 11) | 15 (11 to 18) | 14.87 (11.87 to 18.23) | 14.96 (11.94 to 18.36) | 0.02 (0.02 to 0.03) |
| Argentina | 4448 (3578 to 5502) | 7015 (5584 to 8669) | 13.78 (11.09 to 17.03) | 13.80 (11.10 to 17.05) | 0.00 (0.00 to 0.00) |
| Armenia | 567 (462 to 699) | 632 (509 to 774) | 17.98 (14.51 to 21.96) | 18.22 (14.79 to 22.24) | 0.06 (0.05 to 0.07) |
| Australia | 2865 (2289 to 3556) | 5379 (4316 to 6664) | 15.83 (12.70 to 19.56) | 15.90 (12.76 to 19.67) | 0.02 (0.02 to 0.02) |
| Austria | 1867 (1546 to 2229) | 2478 (2032 to 2989) | 19.82 (16.74 to 23.26) | 19.52 (16.20 to 23.49) | -0.08 (-0.10 to -0.06) |
| Azerbaijan | 1050 (838 to 1300) | 1717 (1366 to 2156) | 16.28 (13.07 to 20.16) | 16.58 (13.33 to 20.54) | 0.07 (0.06 to 0.07) |
| Bahamas | 31 (25 to 39) | 59 (46 to 73) | 14.86 (11.87 to 18.22) | 14.90 (11.89 to 18.27) | 0.01 (0.01 to 0.01) |
| Bahrain | 47 (36 to 60) | 169 (131 to 220) | 12.12 (9.69 to 15.02) | 12.27 (9.81 to 15.18) | 0.05 (0.04 to 0.06) |
| Bangladesh | 12011 (9550 to 14887) | 22730 (18314 to 27880) | 15.05 (12.19 to 18.38) | 14.89 (12.07 to 18.18) | -0.04 (-0.04 to -0.03) |
| Barbados | 41 (32 to 51) | 59 (47 to 73) | 14.84 (11.85 to 18.19) | 14.93 (11.92 to 18.32) | 0.02 (0.02 to 0.02) |
| Belarus | 1746 (1412 to 2138) | 1814 (1457 to 2223) | 15.49 (12.47 to 19.10) | 15.56 (12.53 to 19.18) | 0.01 (0.01 to 0.01) |
| Belgium | 1735 (1390 to 2137) | 2351 (1888 to 2883) | 14.32 (11.69 to 17.40) | 14.43 (11.78 to 17.54) | 0.01 (-0.04 to 0.06) |
| Belize | 21 (17 to 27) | 56 (44 to 69) | 15.08 (12.02 to 18.49) | 15.07 (12.01 to 18.50) | 0.00 (-0.01 to 0.00) |
| Benin | 515 (411 to 639) | 1436 (1132 to 1789) | 14.79 (11.99 to 18.07) | 14.77 (11.96 to 18.06) | -0.01 (-0.01 to 0.00) |
| Bermuda | 9 (7 to 11) | 14 (11 to 18) | 14.87 (11.88 to 18.22) | 14.95 (11.93 to 18.32) | 0.02 (0.01 to 0.02) |
| Bhutan | 68 (53 to 85) | 105 (84 to 130) | 14.91 (12.06 to 18.24) | 14.95 (12.10 to 18.27) | 0.01 (0.00 to 0.01) |
| Bolivia (Plurinational State of) | 698 (564 to 867) | 1541 (1249 to 1888) | 14.79 (11.85 to 18.16) | 14.84 (11.88 to 18.22) | 0.01 (0.01 to 0.01) |
| Bosnia and Herzegovina | 684 (546 to 851) | 680 (540 to 846) | 15.84 (12.81 to 19.47) | 15.86 (12.82 to 19.49) | 0.02 (0.01 to 0.02) |
| Botswana | 141 (111 to 175) | 296 (236 to 366) | 14.68 (11.88 to 17.97) | 14.70 (11.89 to 18.02) | 0.00 (-0.01 to 0.01) |
| Brazil | 19872 (15940 to 24702) | 38739 (31146 to 48348) | 16.54 (13.26 to 20.37) | 16.51 (13.23 to 20.32) | -0.01 (-0.01 to -0.01) |
| Brunei Darussalam | 39 (31 to 48) | 76 (61 to 94) | 18.49 (15.07 to 22.52) | 18.39 (15.00 to 22.40) | -0.01 (-0.02 to 0.00) |
| Bulgaria | 1534 (1220 to 1908) | 1481 (1168 to 1845) | 15.86 (12.83 to 19.50) | 15.87 (12.83 to 19.49) | 0.00 (0.00 to 0.01) |
| Burkina Faso | 1025 (814 to 1269) | 2435 (1930 to 3024) | 14.77 (11.98 to 18.05) | 14.76 (11.96 to 18.06) | 0.00 (0.00 to 0.00) |
| Burundi | 592 (472 to 736) | 1400 (1104 to 1745) | 14.75 (11.94 to 18.04) | 14.91 (12.07 to 18.23) | 0.04 (0.04 to 0.04) |
| Cabo Verde | 43 (35 to 53) | 77 (62 to 95) | 14.64 (11.87 to 17.90) | 14.76 (11.93 to 18.09) | 0.03 (0.03 to 0.03) |
| Cambodia | 1308 (1043 to 1643) | 2517 (2044 to 3120) | 16.63 (13.49 to 20.38) | 16.75 (13.60 to 20.49) | 0.02 (0.02 to 0.02) |
| Cameroon | 1120 (895 to 1393) | 3432 (2701 to 4277) | 14.83 (12.02 to 18.12) | 14.82 (12.00 to 18.12) | 0.00 (0.00 to 0.00) |
| Canada | 5598 (4498 to 6913) | 9947 (7946 to 12292) | 18.56 (14.93 to 22.78) | 18.66 (15.01 to 22.91) | 0.02 (0.01 to 0.02) |
| Central African Republic | 290 (231 to 362) | 586 (464 to 731) | 14.73 (11.92 to 18.03) | 14.69 (11.89 to 18.00) | -0.01 (-0.01 to 0.00) |
| Chad | 659 (527 to 817) | 1798 (1409 to 2248) | 14.78 (11.98 to 18.06) | 14.93 (12.10 to 18.23) | 0.04 (0.03 to 0.04) |
| Chile | 1953 (1582 to 2407) | 3757 (3171 to 4473) | 16.52 (13.41 to 20.34) | 16.89 (14.33 to 20.04) | 0.08 (0.01 to 0.15) |
| China | 180489 (143108 to 225253) | 269321 (218448 to 330653) | 17.42 (13.93 to 21.66) | 16.33 (13.29 to 20.04) | -0.28 (-0.33 to -0.24) |
| Colombia | 3804 (3034 to 4750) | 7846 (6229 to 9690) | 15.00 (11.96 to 18.40) | 14.93 (11.92 to 18.32) | -0.02 (-0.02 to -0.01) |
| Comoros | 50 (39 to 62) | 93 (75 to 115) | 14.82 (12.00 to 18.11) | 14.81 (11.99 to 18.12) | 0.00 (0.00 to 0.00) |
| Congo | 259 (205 to 323) | 622 (496 to 778) | 14.72 (11.92 to 18.02) | 14.82 (12.00 to 18.13) | 0.02 (0.02 to 0.03) |
| Cook Islands | 3 (2 to 3) | 3 (3 to 4) | 16.49 (13.40 to 20.26) | 16.26 (13.21 to 19.95) | -0.05 (-0.06 to -0.05) |
| Costa Rica | 366 (291 to 456) | 767 (610 to 943) | 15.02 (11.98 to 18.44) | 14.93 (11.91 to 18.32) | -0.02 (-0.02 to -0.02) |
| Croatia | 755 (606 to 932) | 862 (693 to 1060) | 14.48 (11.75 to 17.81) | 14.58 (12.10 to 17.79) | -0.31 (-0.61 to -0.01) |
| Cuba | 1607 (1279 to 1989) | 2275 (1827 to 2780) | 15.05 (12.01 to 18.48) | 15.01 (11.97 to 18.41) | -0.01 (-0.01 to -0.01) |
| Cyprus | 133 (108 to 164) | 280 (225 to 348) | 17.03 (14.01 to 20.92) | 17.05 (14.06 to 20.95) | 0.03 (0.00 to 0.07) |
| Czechia | 1960 (1585 to 2420) | 2476 (1983 to 3026) | 17.14 (13.95 to 20.93) | 17.32 (14.11 to 21.19) | 0.04 (0.04 to 0.04) |
| Côte d'Ivoire | 1255 (994 to 1569) | 3044 (2411 to 3793) | 14.97 (12.12 to 18.29) | 14.94 (12.09 to 18.26) | 0.00 (-0.01 to 0.00) |
| Democratic People's Republic of Korea | 2650 (2147 to 3284) | 4131 (3296 to 5167) | 14.48 (11.73 to 17.99) | 14.72 (11.94 to 18.31) | 0.07 (0.06 to 0.08) |
| Democratic Republic of the Congo | 4023 (3198 to 5020) | 9728 (7657 to 12142) | 14.82 (12.00 to 18.11) | 14.76 (11.94 to 18.08) | -0.01 (-0.01 to 0.00) |
| Denmark | 976 (785 to 1216) | 1277 (1016 to 1575) | 15.44 (12.58 to 19.03) | 15.60 (12.70 to 19.24) | 0.04 (0.01 to 0.07) |
| Djibouti | 43 (34 to 55) | 149 (119 to 185) | 14.92 (12.07 to 18.25) | 15.02 (12.15 to 18.33) | 0.02 (0.02 to 0.02) |
| Dominica | 10 (8 to 12) | 11 (9 to 14) | 14.81 (11.83 to 18.12) | 15.01 (11.97 to 18.39) | 0.05 (0.04 to 0.05) |
| Dominican Republic | 822 (654 to 1028) | 1591 (1265 to 1941) | 15.02 (11.98 to 18.44) | 15.03 (11.99 to 18.43) | 0.01 (0.01 to 0.01) |
| Ecuador | 1120 (916 to 1366) | 2493 (2029 to 3032) | 14.70 (12.05 to 17.88) | 14.43 (11.72 to 17.49) | -0.04 (-0.05 to -0.02) |
| Egypt | 5062 (4007 to 6372) | 10366 (8197 to 13007) | 11.95 (9.55 to 14.80) | 12.02 (9.60 to 14.89) | 0.02 (0.02 to 0.02) |
| El Salvador | 622 (496 to 770) | 950 (762 to 1170) | 14.93 (11.91 to 18.32) | 14.78 (11.81 to 18.15) | -0.03 (-0.03 to -0.03) |
| Equatorial Guinea | 45 (36 to 56) | 163 (126 to 204) | 14.69 (11.90 to 17.96) | 14.82 (11.99 to 18.13) | 0.04 (0.03 to 0.05) |
| Eritrea | 345 (270 to 434) | 725 (573 to 905) | 14.65 (11.85 to 17.95) | 14.69 (11.88 to 18.01) | 0.01 (0.01 to 0.01) |
| Estonia | 268 (216 to 330) | 285 (227 to 351) | 15.50 (12.48 to 19.10) | 15.63 (12.59 to 19.26) | 0.03 (0.03 to 0.03) |
| Eswatini | 83 (65 to 103) | 131 (104 to 162) | 14.63 (11.83 to 17.91) | 14.61 (11.82 to 17.92) | 0.00 (0.00 to 0.00) |
| Ethiopia | 5904 (4649 to 7405) | 13309 (10551 to 16784) | 16.52 (13.28 to 20.21) | 16.55 (13.30 to 20.24) | 0.00 (-0.01 to 0.01) |
| Fiji | 99 (79 to 122) | 137 (110 to 169) | 16.38 (13.30 to 20.10) | 16.31 (13.24 to 20.01) | -0.01 (-0.01 to -0.01) |
| Finland | 950 (767 to 1180) | 1371 (1083 to 1698) | 16.30 (13.30 to 20.11) | 16.53 (13.47 to 20.38) | 0.07 (0.03 to 0.11) |
| France | 11027 (8844 to 13590) | 15614 (12453 to 18973) | 16.25 (13.33 to 20.07) | 16.36 (13.40 to 20.19) | 0.02 (0.02 to 0.02) |
| Gabon | 115 (93 to 142) | 216 (173 to 268) | 14.77 (11.95 to 18.09) | 14.74 (11.93 to 18.03) | -0.01 (-0.01 to 0.00) |
| Gambia | 102 (81 to 128) | 261 (206 to 325) | 14.90 (12.06 to 18.21) | 14.80 (11.98 to 18.10) | -0.02 (-0.03 to -0.02) |
| Georgia | 858 (691 to 1055) | 674 (543 to 826) | 14.95 (12.08 to 18.39) | 15.07 (12.17 to 18.50) | 0.02 (0.02 to 0.03) |
| Germany | 17180 (13821 to 21010) | 22803 (18250 to 27921) | 17.51 (14.36 to 21.25) | 17.85 (14.63 to 21.69) | 0.08 (0.07 to 0.09) |
| Ghana | 1610 (1278 to 2005) | 3898 (3112 to 4831) | 14.83 (12.01 to 18.13) | 14.70 (11.90 to 18.00) | -0.03 (-0.03 to -0.03) |
| Greece | 1873 (1500 to 2327) | 2414 (1931 to 2957) | 15.38 (12.48 to 18.91) | 15.33 (12.75 to 18.78) | -0.03 (-0.04 to -0.01) |
| Greenland | 9 (7 to 11) | 12 (9 to 14) | 18.87 (15.18 to 23.15) | 18.86 (15.17 to 23.14) | 0.01 (0.00 to 0.01) |
| Grenada | 12 (10 to 15) | 16 (13 to 20) | 14.86 (11.87 to 18.22) | 14.97 (11.94 to 18.33) | 0.01 (0.01 to 0.02) |
| Guam | 19 (15 to 23) | 29 (24 to 36) | 16.56 (13.45 to 20.35) | 16.41 (13.32 to 20.15) | -0.02 (-0.03 to -0.02) |
| Guatemala | 875 (692 to 1097) | 2024 (1619 to 2508) | 15.01 (11.97 to 18.43) | 14.92 (11.91 to 18.33) | -0.02 (-0.02 to -0.02) |
| Guinea | 689 (559 to 850) | 1454 (1152 to 1805) | 14.85 (12.05 to 18.14) | 14.83 (12.02 to 18.11) | 0.00 (-0.01 to 0.00) |
| Guinea-Bissau | 105 (83 to 131) | 215 (169 to 269) | 14.79 (11.99 to 18.07) | 14.73 (11.92 to 18.01) | -0.02 (-0.02 to -0.01) |
| Guyana | 88 (70 to 110) | 104 (83 to 129) | 15.02 (11.98 to 18.42) | 14.97 (11.94 to 18.35) | -0.01 (-0.01 to 0.00) |
| Haiti | 709 (563 to 881) | 1511 (1206 to 1883) | 14.99 (11.96 to 18.38) | 15.00 (11.97 to 18.42) | 0.01 (0.00 to 0.01) |
| Honduras | 503 (399 to 631) | 1240 (993 to 1545) | 15.00 (11.96 to 18.41) | 14.93 (11.91 to 18.33) | -0.01 (-0.02 to -0.01) |
| Hungary | 1852 (1472 to 2305) | 2062 (1630 to 2551) | 15.73 (12.71 to 19.33) | 15.78 (12.75 to 19.38) | 0.01 (0.01 to 0.01) |
| Iceland | 44 (36 to 54) | 73 (59 to 90) | 16.43 (13.46 to 20.28) | 16.55 (13.54 to 20.44) | 0.03 (0.02 to 0.03) |
| India | 111260 (89309 to 139337) | 217630 (175122 to 265658) | 16.60 (13.36 to 20.30) | 16.52 (13.30 to 20.19) | -0.02 (-0.02 to -0.02) |
| Indonesia | 28051 (22539 to 34920) | 48733 (39155 to 60883) | 18.75 (15.27 to 22.89) | 18.81 (15.33 to 22.99) | 0.01 (0.01 to 0.01) |
| Iran (Islamic Republic of) | 5433 (4205 to 6831) | 10636 (8410 to 13249) | 12.77 (10.19 to 15.84) | 12.75 (10.17 to 15.82) | -0.01 (-0.02 to 0.01) |
| Iraq | 1681 (1330 to 2132) | 4096 (3213 to 5120) | 12.03 (9.63 to 14.88) | 11.94 (9.54 to 14.78) | -0.02 (-0.03 to -0.02) |
| Ireland | 612 (495 to 761) | 1002 (807 to 1234) | 16.35 (13.41 to 20.20) | 16.43 (13.45 to 20.27) | 0.02 (0.01 to 0.02) |
| Israel | 795 (646 to 987) | 1747 (1423 to 2155) | 16.36 (13.39 to 20.18) | 16.41 (13.44 to 20.26) | 0.01 (0.01 to 0.02) |
| Italy | 13936 (11092 to 17079) | 16054 (13176 to 19189) | 20.66 (16.69 to 25.34) | 17.65 (14.98 to 21.03) | -0.70 (-0.82 to -0.58) |
| Jamaica | 314 (250 to 391) | 451 (358 to 558) | 14.95 (11.93 to 18.34) | 15.01 (11.97 to 18.41) | 0.01 (0.01 to 0.01) |
| Japan | 28531 (22776 to 35277) | 40247 (31595 to 49604) | 20.61 (16.74 to 25.33) | 19.84 (16.38 to 24.21) | -0.24 (-0.30 to -0.17) |
| Jordan | 322 (249 to 413) | 1263 (989 to 1592) | 11.97 (9.57 to 14.81) | 12.03 (9.62 to 14.89) | 0.02 (0.02 to 0.02) |
| Kazakhstan | 2429 (1949 to 3003) | 2981 (2381 to 3724) | 16.27 (13.07 to 20.11) | 16.35 (13.14 to 20.23) | 0.02 (0.02 to 0.02) |
| Kenya | 2681 (2100 to 3363) | 6305 (5025 to 7932) | 16.50 (13.26 to 20.17) | 16.40 (13.19 to 20.04) | -0.02 (-0.02 to -0.01) |
| Kiribati | 10 (8 to 12) | 16 (13 to 20) | 16.21 (13.16 to 19.87) | 16.13 (13.10 to 19.79) | -0.01 (-0.02 to -0.01) |
| Kuwait | 162 (124 to 205) | 512 (392 to 655) | 12.22 (9.76 to 15.12) | 12.06 (9.63 to 14.92) | -0.05 (-0.07 to -0.03) |
| Kyrgyzstan | 630 (507 to 777) | 994 (797 to 1235) | 16.32 (13.11 to 20.18) | 16.45 (13.21 to 20.36) | 0.02 (0.02 to 0.03) |
| Lao People's Democratic Republic | 549 (440 to 687) | 1059 (858 to 1315) | 16.84 (13.67 to 20.61) | 16.95 (13.76 to 20.74) | 0.02 (0.02 to 0.02) |
| Latvia | 455 (365 to 564) | 404 (324 to 497) | 15.27 (12.40 to 18.72) | 15.36 (12.48 to 18.83) | -0.03 (-0.10 to 0.05) |
| Lebanon | 307 (245 to 381) | 697 (552 to 864) | 11.83 (9.46 to 14.66) | 11.85 (9.47 to 14.67) | 0.01 (0.00 to 0.01) |
| Lesotho | 173 (139 to 214) | 222 (178 to 275) | 14.44 (11.69 to 17.68) | 14.57 (11.78 to 17.88) | 0.03 (0.03 to 0.03) |
| Liberia | 273 (219 to 338) | 596 (471 to 745) | 14.98 (12.15 to 18.29) | 14.93 (12.09 to 18.24) | 0.00 (0.00 to 0.00) |
| Libya | 387 (306 to 491) | 764 (597 to 970) | 12.09 (9.66 to 14.96) | 11.95 (9.55 to 14.80) | -0.03 (-0.03 to -0.02) |
| Lithuania | 630 (503 to 780) | 610 (480 to 751) | 15.81 (12.69 to 19.51) | 15.86 (12.74 to 19.56) | -0.01 (-0.07 to 0.06) |
| Luxembourg | 80 (65 to 99) | 148 (119 to 182) | 17.94 (14.72 to 22.08) | 18.25 (14.95 to 22.44) | 0.00 (-0.05 to 0.04) |
| Madagascar | 1284 (1020 to 1596) | 3071 (2413 to 3831) | 14.89 (12.07 to 18.19) | 14.81 (12.00 to 18.11) | -0.02 (-0.02 to -0.01) |
| Malawi | 1031 (816 to 1282) | 2072 (1618 to 2592) | 14.80 (11.98 to 18.10) | 14.70 (11.90 to 18.00) | -0.02 (-0.03 to -0.02) |
| Malaysia | 2441 (1969 to 3028) | 5168 (4171 to 6360) | 16.98 (13.77 to 20.78) | 17.11 (13.89 to 20.93) | 0.03 (0.03 to 0.03) |
| Maldives | 28 (23 to 36) | 81 (63 to 101) | 17.36 (14.08 to 21.28) | 17.57 (14.28 to 21.44) | 0.04 (0.02 to 0.07) |
| Mali | 938 (750 to 1156) | 2510 (1980 to 3132) | 14.85 (12.03 to 18.14) | 14.90 (12.07 to 18.19) | 0.01 (0.01 to 0.01) |
| Malta | 64 (51 to 79) | 107 (85 to 133) | 16.32 (13.38 to 20.15) | 16.50 (13.51 to 20.37) | 0.03 (0.01 to 0.05) |
| Marshall Islands | 6 (4 to 7) | 8 (6 to 10) | 16.37 (13.30 to 20.08) | 16.48 (13.38 to 20.24) | 0.03 (0.02 to 0.03) |
| Mauritania | 229 (183 to 283) | 499 (399 to 617) | 14.80 (11.99 to 18.10) | 14.86 (12.04 to 18.15) | 0.01 (0.01 to 0.02) |
| Mauritius | 160 (129 to 198) | 248 (199 to 306) | 16.84 (13.67 to 20.59) | 16.89 (13.71 to 20.66) | 0.01 (0.01 to 0.01) |
| Mexico | 10788 (8585 to 13424) | 21186 (16877 to 26243) | 16.60 (13.31 to 20.44) | 16.54 (13.26 to 20.36) | -0.01 (-0.01 to -0.01) |
| Micronesia (Federated States of) | 13 (11 to 17) | 15 (12 to 18) | 16.38 (13.31 to 20.11) | 16.33 (13.25 to 20.05) | -0.01 (-0.01 to -0.01) |
| Monaco | 7 (6 to 9) | 10 (8 to 12) | 16.28 (13.35 to 20.10) | 16.40 (13.43 to 20.22) | 0.02 (0.01 to 0.02) |
| Mongolia | 283 (224 to 354) | 480 (382 to 605) | 16.54 (13.28 to 20.48) | 16.45 (13.22 to 20.36) | -0.01 (-0.01 to -0.01) |
| Montenegro | 99 (80 to 122) | 116 (92 to 144) | 15.84 (12.82 to 19.47) | 15.86 (12.83 to 19.49) | 0.01 (0.01 to 0.01) |
| Morocco | 2436 (1940 to 3051) | 4263 (3376 to 5330) | 11.90 (9.51 to 14.74) | 11.89 (9.50 to 14.73) | 0.00 (0.00 to 0.00) |
| Mozambique | 1440 (1146 to 1781) | 3219 (2531 to 4028) | 14.75 (11.95 to 18.03) | 14.66 (11.87 to 17.96) | -0.02 (-0.02 to -0.02) |
| Myanmar | 5581 (4497 to 6942) | 8816 (7132 to 10860) | 16.86 (13.68 to 20.63) | 16.76 (13.60 to 20.51) | -0.02 (-0.02 to -0.02) |
| Namibia | 154 (122 to 191) | 289 (232 to 356) | 14.71 (11.91 to 18.02) | 14.65 (11.85 to 17.96) | -0.01 (-0.01 to -0.01) |
| Nauru | 1 (1 to 2) | 1 (1 to 2) | 16.67 (13.55 to 20.45) | 16.28 (13.22 to 19.97) | -0.08 (-0.08 to -0.07) |
| Nepal | 2170 (1727 to 2690) | 4058 (3265 to 4973) | 14.90 (12.07 to 18.20) | 14.76 (11.97 to 18.03) | -0.03 (-0.04 to -0.03) |
| Netherlands | 2773 (2234 to 3435) | 3971 (3185 to 4898) | 16.30 (13.36 to 20.11) | 16.46 (13.48 to 20.32) | 0.03 (0.03 to 0.04) |
| New Zealand | 608 (484 to 761) | 1055 (867 to 1274) | 16.74 (13.38 to 20.90) | 16.38 (13.66 to 19.68) | -0.05 (-0.18 to 0.08) |
| Nicaragua | 407 (320 to 511) | 874 (701 to 1077) | 14.92 (11.91 to 18.30) | 14.91 (11.91 to 18.30) | 0.00 (0.00 to 0.00) |
| Niger | 818 (644 to 1022) | 2526 (1973 to 3169) | 14.88 (12.05 to 18.18) | 14.84 (12.02 to 18.13) | -0.01 (-0.02 to -0.01) |
| Nigeria | 11335 (9053 to 14146) | 27647 (21835 to 34676) | 16.56 (13.34 to 20.23) | 16.39 (13.17 to 20.03) | -0.04 (-0.05 to -0.03) |
| Niue | 0 (0 to 0) | 0 (0 to 0) | 16.28 (13.22 to 20.00) | 16.32 (13.25 to 20.02) | 0.01 (0.00 to 0.01) |
| North Macedonia | 307 (247 to 378) | 404 (320 to 502) | 15.92 (12.87 to 19.57) | 15.96 (12.90 to 19.61) | 0.01 (0.01 to 0.01) |
| Northern Mariana Islands | 6 (5 to 7) | 8 (6 to 10) | 16.60 (13.46 to 20.35) | 16.50 (13.40 to 20.27) | -0.01 (-0.03 to 0.00) |
| Norway | 854 (673 to 1064) | 1188 (943 to 1470) | 16.34 (13.13 to 20.35) | 16.52 (13.27 to 20.57) | 0.01 (-0.01 to 0.03) |
| Oman | 178 (139 to 224) | 478 (361 to 613) | 12.21 (9.75 to 15.11) | 12.20 (9.75 to 15.10) | 0.00 (-0.01 to 0.01) |
| Pakistan | 14198 (11371 to 17734) | 30813 (24837 to 38384) | 16.72 (13.42 to 20.47) | 16.61 (13.35 to 20.31) | -0.02 (-0.02 to -0.02) |
| Palau | 2 (2 to 3) | 3 (2 to 4) | 16.44 (13.35 to 20.19) | 16.71 (13.56 to 20.56) | 0.05 (0.03 to 0.06) |
| Palestine | 177 (140 to 226) | 484 (381 to 609) | 11.80 (9.43 to 14.62) | 11.86 (9.48 to 14.69) | 0.01 (0.01 to 0.02) |
| Panama | 296 (237 to 369) | 659 (525 to 808) | 15.09 (12.02 to 18.51) | 15.04 (11.99 to 18.45) | -0.01 (-0.01 to -0.01) |
| Papua New Guinea | 525 (418 to 651) | 1373 (1106 to 1699) | 16.56 (13.46 to 20.31) | 16.59 (13.48 to 20.35) | 0.01 (0.01 to 0.01) |
| Paraguay | 472 (375 to 585) | 985 (785 to 1208) | 15.01 (11.97 to 18.40) | 15.01 (11.97 to 18.40) | 0.00 (0.00 to 0.00) |
| Peru | 2501 (2013 to 3073) | 5240 (4212 to 6426) | 14.87 (11.91 to 18.26) | 14.87 (11.90 to 18.26) | 0.00 (0.00 to 0.00) |
| Philippines | 9330 (7468 to 11650) | 18676 (15033 to 22778) | 18.83 (15.33 to 22.98) | 18.68 (15.14 to 22.79) | -0.03 (-0.04 to -0.03) |
| Poland | 7512 (6037 to 9269) | 9393 (7522 to 11651) | 18.92 (15.33 to 23.41) | 19.04 (15.43 to 23.55) | -0.03 (-0.06 to 0.00) |
| Portugal | 1828 (1471 to 2259) | 2611 (2097 to 3174) | 15.95 (13.07 to 19.68) | 16.08 (13.28 to 19.79) | 0.03 (0.02 to 0.03) |
| Puerto Rico | 539 (429 to 664) | 766 (605 to 937) | 14.92 (11.91 to 18.32) | 14.91 (11.91 to 18.30) | 0.00 (0.00 to 0.00) |
| Qatar | 43 (32 to 55) | 319 (232 to 420) | 12.49 (9.98 to 15.48) | 12.48 (9.97 to 15.47) | 0.03 (0.02 to 0.05) |
| Republic of Korea | 7092 (5659 to 8724) | 11793 (9350 to 14631) | 17.94 (14.63 to 21.87) | 18.22 (14.86 to 22.20) | 0.05 (0.05 to 0.06) |
| Republic of Moldova | 671 (538 to 818) | 697 (559 to 859) | 15.53 (12.50 to 19.13) | 15.62 (12.57 to 19.24) | 0.02 (0.02 to 0.02) |
| Romania | 3411 (2713 to 4221) | 3766 (2971 to 4590) | 14.49 (11.64 to 17.82) | 14.47 (11.55 to 17.82) | -0.20 (-0.31 to -0.10) |
| Russian Federation | 27303 (21934 to 33569) | 30473 (24666 to 37726) | 17.18 (13.92 to 21.03) | 17.24 (13.97 to 21.09) | 0.01 (0.00 to 0.01) |
| Rwanda | 751 (593 to 934) | 1497 (1193 to 1866) | 14.73 (11.92 to 18.02) | 14.67 (11.87 to 17.97) | -0.01 (-0.02 to -0.01) |
| Saint Kitts and Nevis | 6 (5 to 7) | 9 (7 to 12) | 14.87 (11.87 to 18.23) | 14.95 (11.93 to 18.31) | 0.02 (0.01 to 0.02) |
| Saint Lucia | 17 (13 to 21) | 31 (25 to 38) | 14.87 (11.87 to 18.22) | 15.02 (11.98 to 18.41) | 0.04 (0.03 to 0.04) |
| Saint Vincent and the Grenadines | 14 (11 to 17) | 19 (15 to 24) | 14.91 (11.90 to 18.27) | 15.10 (12.04 to 18.53) | 0.04 (0.04 to 0.04) |
| Samoa | 22 (18 to 28) | 30 (25 to 37) | 16.44 (13.35 to 20.20) | 16.42 (13.33 to 20.15) | -0.01 (-0.01 to -0.01) |
| San Marino | 5 (4 to 6) | 8 (7 to 10) | 16.36 (13.41 to 20.20) | 16.38 (13.41 to 20.19) | 0.01 (0.00 to 0.01) |
| Sao Tome and Principe | 14 (11 to 17) | 25 (20 to 31) | 14.74 (11.92 to 18.03) | 14.83 (12.00 to 18.14) | 0.02 (0.02 to 0.02) |
| Saudi Arabia | 1427 (1114 to 1809) | 3964 (3013 to 5110) | 12.20 (9.75 to 15.09) | 12.21 (9.75 to 15.11) | 0.00 (0.00 to 0.01) |
| Senegal | 818 (650 to 1015) | 1808 (1442 to 2235) | 14.85 (12.03 to 18.14) | 14.83 (12.01 to 18.12) | 0.00 (0.00 to 0.00) |
| Serbia | 1567 (1256 to 1931) | 1840 (1465 to 2271) | 15.79 (12.61 to 19.37) | 15.88 (12.68 to 19.48) | -0.05 (-0.11 to 0.01) |
| Seychelles | 11 (9 to 14) | 18 (15 to 23) | 16.86 (13.69 to 20.62) | 17.07 (13.87 to 20.85) | 0.05 (0.05 to 0.06) |
| Sierra Leone | 471 (380 to 578) | 981 (778 to 1216) | 14.91 (12.09 to 18.20) | 14.87 (12.05 to 18.18) | 0.00 (-0.01 to 0.00) |
| Singapore | 508 (406 to 628) | 1205 (963 to 1487) | 18.15 (14.81 to 22.12) | 18.23 (14.86 to 22.22) | 0.02 (0.01 to 0.03) |
| Slovakia | 963 (783 to 1182) | 1194 (962 to 1481) | 17.51 (14.28 to 21.44) | 17.57 (14.33 to 21.51) | -0.08 (-0.14 to -0.02) |
| Slovenia | 321 (260 to 399) | 440 (349 to 542) | 14.89 (12.14 to 18.35) | 15.08 (12.29 to 18.58) | -0.01 (-0.07 to 0.05) |
| Solomon Islands | 42 (33 to 53) | 90 (72 to 111) | 16.62 (13.51 to 20.40) | 16.43 (13.35 to 20.17) | -0.04 (-0.04 to -0.04) |
| Somalia | 798 (624 to 1003) | 2147 (1674 to 2688) | 14.86 (12.03 to 18.17) | 14.71 (11.90 to 18.02) | -0.03 (-0.03 to -0.03) |
| South Africa | 4875 (3935 to 6102) | 8563 (6921 to 10485) | 16.31 (13.12 to 19.93) | 16.32 (13.12 to 19.93) | 0.00 (0.00 to 0.01) |
| South Sudan | 648 (514 to 805) | 1037 (819 to 1287) | 15.08 (12.21 to 18.42) | 14.91 (12.09 to 18.20) | -0.04 (-0.04 to -0.03) |
| Spain | 6720 (5386 to 8335) | 9998 (7930 to 12262) | 15.07 (12.31 to 18.34) | 14.89 (12.28 to 18.21) | -0.06 (-0.08 to -0.04) |
| Sri Lanka | 2477 (2000 to 3070) | 3989 (3224 to 4914) | 17.41 (14.14 to 21.35) | 16.81 (13.64 to 20.57) | -0.16 (-0.19 to -0.13) |
| Sudan | 1796 (1422 to 2275) | 4000 (3142 to 5043) | 11.92 (9.53 to 14.77) | 11.95 (9.54 to 14.79) | 0.01 (0.01 to 0.01) |
| Suriname | 49 (39 to 61) | 90 (71 to 110) | 15.02 (11.98 to 18.44) | 14.94 (11.93 to 18.32) | -0.02 (-0.02 to -0.02) |
| Sweden | 2130 (1716 to 2625) | 2879 (2284 to 3531) | 19.36 (15.86 to 23.69) | 19.74 (16.15 to 24.26) | 0.07 (0.03 to 0.10) |
| Switzerland | 1141 (906 to 1407) | 1751 (1389 to 2147) | 13.60 (11.00 to 16.69) | 13.72 (11.09 to 16.83) | 0.04 (-0.02 to 0.10) |
| Syrian Arab Republic | 1111 (874 to 1412) | 1594 (1247 to 2011) | 11.97 (9.57 to 14.82) | 11.83 (9.43 to 14.65) | -0.03 (-0.05 to -0.02) |
| Taiwan (Province of China) | 2684 (2184 to 3328) | 4180 (3452 to 5033) | 14.62 (11.88 to 18.04) | 13.97 (12.03 to 16.12) | -0.17 (-0.21 to -0.14) |
| Tajikistan | 706 (562 to 882) | 1409 (1122 to 1745) | 16.50 (13.25 to 20.42) | 16.73 (13.43 to 20.77) | 0.05 (0.05 to 0.06) |
| Thailand | 8174 (6539 to 10111) | 13903 (11073 to 17084) | 16.87 (13.69 to 20.65) | 16.87 (13.69 to 20.65) | 0.00 (0.00 to 0.00) |
| Timor-Leste | 99 (79 to 123) | 200 (161 to 248) | 17.09 (13.87 to 20.93) | 17.03 (13.81 to 20.85) | -0.02 (-0.02 to -0.02) |
| Togo | 373 (292 to 467) | 932 (740 to 1158) | 14.78 (11.97 to 18.05) | 14.68 (11.88 to 17.97) | -0.03 (-0.03 to -0.02) |
| Tokelau | 0 (0 to 0) | 0 (0 to 0) | 16.33 (13.27 to 20.07) | 16.44 (13.36 to 20.17) | 0.02 (0.01 to 0.03) |
| Tonga | 13 (11 to 17) | 16 (13 to 19) | 16.34 (13.28 to 20.06) | 16.27 (13.22 to 19.97) | -0.02 (-0.02 to -0.01) |
| Trinidad and Tobago | 154 (124 to 192) | 245 (196 to 304) | 14.99 (11.96 to 18.37) | 15.01 (11.98 to 18.41) | 0.01 (0.00 to 0.01) |
| Tunisia | 814 (648 to 1023) | 1468 (1165 to 1831) | 11.90 (9.51 to 14.74) | 11.85 (9.47 to 14.68) | -0.01 (-0.02 to -0.01) |
| Turkmenistan | 488 (388 to 609) | 784 (627 to 975) | 16.38 (13.16 to 20.27) | 16.65 (13.39 to 20.61) | 0.06 (0.06 to 0.06) |
| Tuvalu | 1 (1 to 2) | 2 (2 to 2) | 16.12 (13.10 to 19.78) | 16.41 (13.32 to 20.14) | 0.06 (0.06 to 0.06) |
| Türkiye | 5868 (4636 to 7331) | 10789 (8473 to 13483) | 12.27 (9.78 to 15.18) | 12.28 (9.78 to 15.19) | 0.02 (0.01 to 0.03) |
| Uganda | 1796 (1420 to 2241) | 4482 (3514 to 5620) | 14.82 (12.00 to 18.12) | 14.69 (11.89 to 17.99) | -0.02 (-0.03 to -0.02) |
| Ukraine | 10031 (8096 to 12377) | 9524 (7628 to 11800) | 17.16 (13.90 to 20.99) | 17.26 (13.98 to 21.11) | 0.02 (0.02 to 0.02) |
| United Arab Emirates | 175 (131 to 226) | 1106 (794 to 1486) | 12.44 (9.94 to 15.41) | 12.64 (10.10 to 15.66) | 0.09 (0.06 to 0.12) |
| United Kingdom | 12540 (9981 to 15567) | 16585 (13271 to 20339) | 18.02 (14.58 to 22.33) | 18.02 (14.74 to 22.22) | -0.08 (-0.17 to 0.01) |
| United Republic of Tanzania | 2756 (2181 to 3425) | 6444 (5106 to 7991) | 14.80 (11.98 to 18.09) | 14.78 (11.97 to 18.07) | 0.00 (0.00 to 0.00) |
| United States Virgin Islands | 14 (11 to 17) | 19 (15 to 24) | 14.89 (11.89 to 18.26) | 14.95 (11.93 to 18.32) | 0.01 (0.01 to 0.01) |
| United States of America | 55733 (44254 to 69717) | 79241 (67152 to 94333) | 19.42 (15.46 to 24.23) | 18.31 (15.71 to 21.81) | -0.76 (-0.96 to -0.56) |
| Uruguay | 523 (422 to 645) | 663 (533 to 818) | 15.07 (12.24 to 18.59) | 15.06 (12.23 to 18.58) | 0.00 (-0.01 to 0.00) |
| Uzbekistan | 2830 (2266 to 3525) | 5129 (4112 to 6416) | 16.42 (13.19 to 20.31) | 16.56 (13.30 to 20.51) | 0.03 (0.03 to 0.03) |
| Vanuatu | 19 (15 to 24) | 42 (34 to 52) | 16.55 (13.45 to 20.30) | 16.38 (13.31 to 20.10) | -0.03 (-0.03 to -0.03) |
| Venezuela (Bolivarian Republic of) | 2166 (1721 to 2700) | 4168 (3309 to 5137) | 14.98 (11.95 to 18.37) | 14.91 (11.90 to 18.28) | 0.00 (-0.01 to 0.00) |
| Viet Nam | 9530 (7686 to 11838) | 16528 (13388 to 20449) | 16.69 (13.55 to 20.43) | 16.83 (13.67 to 20.59) | 0.02 (0.02 to 0.03) |
| Yemen | 1124 (884 to 1431) | 3009 (2358 to 3804) | 11.85 (9.48 to 14.68) | 11.88 (9.49 to 14.72) | 0.01 (0.00 to 0.01) |
| Zambia | 822 (647 to 1027) | 2064 (1615 to 2585) | 14.91 (12.08 to 18.20) | 14.81 (11.98 to 18.12) | -0.03 (-0.03 to -0.02) |
| Zimbabwe | 1092 (860 to 1362) | 1708 (1347 to 2125) | 14.77 (11.95 to 18.07) | 14.67 (11.88 to 17.96) | -0.02 (-0.02 to -0.02) |

# Table S3. The prevalence cases and age-standardized prevalence rate of myocarditis in 1990 and 2021, and EAPC in age-standardized prevalence rate from 1990 to 2021

| **Characteristics** | **Prevalence cases (95% UI)** | | **ASPR (95% UI)** | | **EAPC in ASPR (95% CI)** |
| --- | --- | --- | --- | --- | --- |
|  | **1990** | **2021** | **1990** | **2021** | **1990-2021** |
| **Global** | 320623 (268557 to 371912) | 505030 (432295 to 587819) | 6.35 (5.37 to 7.36) | 6.41 (5.48 to 7.44) | 0.02 (-0.12 to 0.15) |
| **Gender** |  |  |  |  |  |
| Male | 171476 (143274 to 201040) | 270326 (230547 to 316677) | 6.95 (5.90 to 8.13) | 6.99 (5.98 to 8.16) | 0.02 (-0.10 to 0.13) |
| Female | 149147 (124312 to 172590) | 234704 (201726 to 270946) | 5.81 (4.89 to 6.69) | 5.87 (4.97 to 6.78) | 0.01 (-0.15 to 0.16) |
| **SDI** |  |  |  |  |  |
| High-middle SDI | 66246 (55865 to 77432) | 97692 (83535 to 112985) | 6.66 (5.67 to 7.68) | 7.19 (6.13 to 8.34) | 0.25 (0.00 to 0.50) |
| High SDI | 80395 (68587 to 92261) | 120333 (104227 to 137304) | 9.62 (8.15 to 11.18) | 12.03 (10.04 to 14.25) | 0.73 (0.36 to 1.10) |
| Low-middle SDI | 56577 (46744 to 66388) | 89181 (74276 to 106068) | 5.32 (4.47 to 6.27) | 5.09 (4.28 to 6.04) | -0.14 (-0.15 to -0.13) |
| Low SDI | 24256 (19672 to 28897) | 44139 (36203 to 52607) | 5.18 (4.35 to 6.12) | 4.67 (3.90 to 5.58) | -0.38 (-0.40 to -0.37) |
| Middle SDI | 92846 (76997 to 109401) | 153276 (131448 to 179398) | 6.02 (5.10 to 7.05) | 6.42 (5.50 to 7.46) | 0.27 (0.22 to 0.31) |
| **Region** |  |  |  |  |  |
| East Asia | 75805 (63019 to 89443) | 127373 (108722 to 148737) | 6.99 (5.89 to 8.10) | 8.69 (7.35 to 10.19) | 0.90 (0.78 to 1.03) |
| Southeast Asia | 23753 (19685 to 28046) | 37533 (31280 to 45007) | 5.78 (4.88 to 6.77) | 5.75 (4.85 to 6.77) | -0.03 (-0.05 to -0.01) |
| Oceania | 239 (194 to 287) | 522 (428 to 631) | 4.46 (3.75 to 5.36) | 4.45 (3.71 to 5.39) | 0.00 (0.00 to 0.00) |
| Central Asia | 3670 (3075 to 4284) | 5387 (4566 to 6332) | 5.59 (4.76 to 6.53) | 5.94 (5.08 to 6.91) | 0.35 (0.24 to 0.47) |
| Central Europe | 9781 (8349 to 11228) | 11797 (10216 to 13496) | 8.12 (6.87 to 9.29) | 10.13 (8.58 to 11.77) | 0.48 (0.34 to 0.61) |
| Eastern Europe | 15643 (13279 to 18339) | 13736 (11550 to 16438) | 7.12 (6.03 to 8.26) | 6.13 (5.18 to 7.24) | -0.51 (-0.62 to -0.39) |
| High-income Asia Pacific | 13700 (11664 to 16026) | 23177 (19959 to 26523) | 8.42 (7.13 to 9.75) | 14.13 (11.61 to 17.00) | 1.28 (0.97 to 1.59) |
| Australasia | 2387 (2024 to 2746) | 3635 (3084 to 4229) | 12.69 (10.65 to 14.90) | 12.90 (10.78 to 15.61) | -0.55 (-1.37 to 0.28) |
| Western Europe | 28590 (24659 to 33052) | 40204 (34568 to 46404) | 7.04 (6.08 to 8.01) | 8.22 (7.02 to 9.42) | 0.71 (0.03 to 1.39) |
| Southern Latin America | 2446 (2069 to 2864) | 3443 (2936 to 4085) | 5.05 (4.29 to 5.90) | 4.73 (4.06 to 5.52) | -0.27 (-0.40 to -0.13) |
| High-income North America | 36561 (30926 to 42846) | 50292 (42892 to 58478) | 14.00 (11.61 to 16.67) | 15.94 (13.05 to 19.33) | 0.42 (0.10 to 0.75) |
| Caribbean | 1696 (1422 to 1981) | 2636 (2212 to 3088) | 5.13 (4.32 to 5.98) | 5.51 (4.59 to 6.42) | 0.27 (0.19 to 0.35) |
| Andean Latin America | 1402 (1176 to 1642) | 2579 (2153 to 3094) | 4.48 (3.78 to 5.30) | 4.10 (3.42 to 4.89) | -0.40 (-0.47 to -0.33) |
| Central Latin America | 6214 (5159 to 7375) | 12721 (10638 to 15217) | 4.53 (3.78 to 5.44) | 5.14 (4.32 to 6.11) | 0.48 (0.45 to 0.52) |
| Tropical Latin America | 7038 (5796 to 8328) | 13410 (11284 to 15816) | 5.26 (4.41 to 6.25) | 5.90 (4.98 to 6.85) | 0.30 (0.20 to 0.40) |
| North Africa and Middle East | 13595 (11269 to 15805) | 24229 (20287 to 28391) | 4.37 (3.70 to 5.08) | 4.27 (3.61 to 4.98) | -0.05 (-0.06 to -0.03) |
| South Asia | 54479 (44789 to 64459) | 88699 (73758 to 106274) | 5.46 (4.56 to 6.43) | 5.24 (4.40 to 6.22) | -0.11 (-0.12 to -0.09) |
| Central Sub-Saharan Africa | 2045 (1643 to 2485) | 4293 (3477 to 5207) | 4.39 (3.65 to 5.29) | 4.02 (3.32 to 4.88) | -0.35 (-0.37 to -0.33) |
| Eastern Sub-Saharan Africa | 9702 (7691 to 11878) | 14872 (12164 to 17885) | 5.36 (4.45 to 6.32) | 4.38 (3.66 to 5.31) | -0.77 (-0.82 to -0.71) |
| Southern Sub-Saharan Africa | 2313 (1901 to 2758) | 3401 (2834 to 4095) | 5.04 (4.23 to 5.97) | 4.68 (3.91 to 5.61) | -0.21 (-0.25 to -0.17) |
| Western Sub-Saharan Africa | 9566 (7635 to 11598) | 21091 (17118 to 25512) | 5.25 (4.38 to 6.25) | 4.89 (4.06 to 5.86) | -0.28 (-0.29 to -0.26) |

# Table S4. The prevalence cases and age-standardized prevalence rate of myocarditis in 1990 and 2021, and EAPC in age-standardized prevalence rate from 1990 to 2021 in 204 countries and territories

| **Characteristics** | **Prevalence cases (95% UI)** | | **ASPR (95% UI)** | | **EAPC in ASPR**  **(95% CI)** |
| --- | --- | --- | --- | --- | --- |
|  | **1990** | **2021** | **1990** | **2021** | **1990-2021** |
| Afghanistan | 287 (237 to 345) | 791 (645 to 963) | 3.41 (2.84 to 4.06) | 3.46 (2.88 to 4.11) | 0.05 (0.04 to 0.05) |
| Albania | 254 (211 to 296) | 239 (205 to 276) | 7.96 (6.74 to 9.08) | 9.04 (7.66 to 10.42) | 0.36 (0.21 to 0.52) |
| Algeria | 762 (637 to 914) | 1586 (1313 to 1895) | 3.73 (3.15 to 4.39) | 3.92 (3.27 to 4.55) | 0.19 (0.18 to 0.21) |
| American Samoa | 2 (1 to 2) | 2 (2 to 2) | 4.11 (3.39 to 5.02) | 4.17 (3.45 to 5.08) | 0.05 (0.05 to 0.06) |
| Andorra | 4 (4 to 5) | 7 (6 to 8) | 9.68 (8.20 to 11.26) | 8.93 (7.64 to 10.29) | -0.30 (-0.34 to -0.26) |
| Angola | 407 (325 to 503) | 996 (807 to 1209) | 4.56 (3.78 to 5.49) | 3.99 (3.30 to 4.84) | -0.50 (-0.53 to -0.48) |
| Antigua and Barbuda | 5 (4 to 6) | 7 (6 to 8) | 8.42 (7.06 to 9.77) | 8.11 (6.79 to 9.40) | -0.12 (-0.25 to 0.01) |
| Argentina | 1587 (1341 to 1862) | 2135 (1785 to 2539) | 4.85 (4.13 to 5.69) | 4.42 (3.71 to 5.21) | -0.41 (-0.54 to -0.28) |
| Armenia | 177 (148 to 211) | 165 (137 to 203) | 5.48 (4.64 to 6.46) | 4.88 (4.09 to 5.92) | -0.38 (-0.51 to -0.25) |
| Australia | 2024 (1705 to 2341) | 2903 (2469 to 3352) | 12.97 (10.78 to 15.27) | 12.17 (10.15 to 14.63) | -0.87 (-1.69 to -0.04) |
| Austria | 470 (394 to 567) | 786 (673 to 925) | 5.11 (4.37 to 5.98) | 7.52 (6.47 to 8.68) | 1.72 (0.89 to 2.56) |
| Azerbaijan | 423 (358 to 491) | 652 (555 to 765) | 6.10 (5.21 to 7.09) | 6.60 (5.65 to 7.63) | 0.13 (0.08 to 0.17) |
| Bahamas | 11 (9 to 13) | 18 (15 to 22) | 4.83 (4.06 to 5.73) | 4.91 (4.11 to 5.81) | 0.09 (0.00 to 0.18) |
| Bahrain | 14 (11 to 17) | 48 (38 to 60) | 3.46 (2.87 to 4.14) | 3.56 (2.94 to 4.23) | 0.10 (0.08 to 0.13) |
| Bangladesh | 4705 (3876 to 5548) | 7462 (6248 to 8850) | 4.85 (4.12 to 5.71) | 4.87 (4.12 to 5.75) | 0.05 (0.02 to 0.07) |
| Barbados | 14 (12 to 17) | 21 (18 to 25) | 5.46 (4.64 to 6.34) | 6.63 (5.61 to 7.66) | 0.66 (0.58 to 0.74) |
| Belarus | 611 (523 to 709) | 523 (435 to 627) | 5.85 (5.00 to 6.85) | 5.02 (4.24 to 5.90) | -0.56 (-0.69 to -0.44) |
| Belgium | 466 (380 to 563) | 835 (706 to 969) | 4.03 (3.36 to 4.78) | 6.52 (5.47 to 7.61) | 1.99 (1.02 to 2.97) |
| Belize | 9 (8 to 11) | 19 (16 to 22) | 5.36 (4.55 to 6.21) | 4.90 (4.12 to 5.80) | -0.26 (-0.41 to -0.10) |
| Benin | 200 (158 to 247) | 501 (405 to 610) | 4.59 (3.80 to 5.52) | 4.39 (3.60 to 5.27) | -0.16 (-0.17 to -0.14) |
| Bermuda | 3 (3 to 4) | 5 (4 to 6) | 5.54 (4.67 to 6.41) | 6.75 (5.70 to 7.75) | 0.59 (0.42 to 0.76) |
| Bhutan | 28 (23 to 33) | 35 (29 to 41) | 5.02 (4.25 to 5.86) | 5.00 (4.27 to 5.90) | -0.01 (-0.03 to 0.01) |
| Bolivia (Plurinational State of) | 211 (175 to 251) | 423 (349 to 510) | 4.17 (3.49 to 5.00) | 4.05 (3.37 to 4.88) | -0.10 (-0.11 to -0.09) |
| Bosnia and Herzegovina | 206 (172 to 246) | 190 (157 to 232) | 4.84 (4.09 to 5.72) | 4.93 (4.16 to 5.83) | 0.07 (0.05 to 0.09) |
| Botswana | 49 (40 to 60) | 92 (76 to 111) | 4.52 (3.75 to 5.39) | 4.38 (3.64 to 5.25) | -0.05 (-0.09 to -0.01) |
| Brazil | 6876 (5659 to 8139) | 13115 (11042 to 15468) | 5.28 (4.43 to 6.28) | 5.95 (5.02 to 6.90) | 0.31 (0.21 to 0.42) |
| Brunei Darussalam | 29 (24 to 36) | 42 (35 to 51) | 10.87 (8.92 to 12.97) | 11.12 (9.12 to 13.39) | 0.04 (-0.03 to 0.11) |
| Bulgaria | 542 (453 to 632) | 580 (486 to 678) | 6.14 (5.17 to 7.10) | 8.52 (7.14 to 10.01) | 0.54 (0.37 to 0.72) |
| Burkina Faso | 401 (316 to 498) | 879 (701 to 1082) | 4.65 (3.83 to 5.62) | 4.47 (3.68 to 5.40) | -0.13 (-0.15 to -0.12) |
| Burundi | 252 (196 to 315) | 426 (344 to 514) | 4.85 (3.98 to 5.83) | 4.15 (3.41 to 5.04) | -0.60 (-0.63 to -0.56) |
| Cabo Verde | 22 (17 to 28) | 26 (21 to 32) | 5.91 (4.80 to 7.16) | 5.04 (4.15 to 6.03) | -0.62 (-0.67 to -0.58) |
| Cambodia | 391 (317 to 469) | 733 (600 to 882) | 4.66 (3.91 to 5.59) | 4.85 (4.08 to 5.77) | 0.16 (0.15 to 0.17) |
| Cameroon | 422 (331 to 516) | 1137 (912 to 1388) | 4.56 (3.70 to 5.47) | 4.32 (3.57 to 5.19) | -0.20 (-0.21 to -0.19) |
| Canada | 2735 (2281 to 3183) | 5585 (4696 to 6588) | 10.74 (8.88 to 12.73) | 17.22 (14.01 to 20.77) | 1.85 (1.26 to 2.44) |
| Central African Republic | 98 (79 to 119) | 170 (138 to 205) | 4.30 (3.57 to 5.20) | 3.99 (3.30 to 4.83) | -0.28 (-0.29 to -0.26) |
| Chad | 248 (198 to 309) | 671 (528 to 825) | 4.54 (3.78 to 5.45) | 4.47 (3.69 to 5.36) | -0.06 (-0.07 to -0.05) |
| Chile | 664 (558 to 777) | 1090 (946 to 1284) | 5.37 (4.53 to 6.32) | 5.32 (4.66 to 6.15) | 0.09 (-0.05 to 0.23) |
| China | 73867 (61357 to 87236) | 123748 (105421 to 144606) | 7.05 (5.94 to 8.18) | 8.74 (7.38 to 10.25) | 0.88 (0.76 to 1.00) |
| Colombia | 1243 (1031 to 1471) | 2921 (2436 to 3421) | 4.47 (3.73 to 5.33) | 6.01 (5.02 to 7.01) | 0.98 (0.88 to 1.07) |
| Comoros | 19 (15 to 23) | 27 (22 to 32) | 4.68 (3.88 to 5.61) | 4.12 (3.41 to 4.96) | -0.45 (-0.46 to -0.43) |
| Congo | 84 (68 to 102) | 173 (140 to 211) | 4.25 (3.52 to 5.12) | 3.98 (3.28 to 4.80) | -0.28 (-0.31 to -0.26) |
| Cook Islands | 1 (1 to 1) | 1 (1 to 1) | 4.05 (3.32 to 4.97) | 3.99 (3.27 to 4.90) | -0.06 (-0.06 to -0.05) |
| Costa Rica | 179 (151 to 209) | 333 (285 to 384) | 6.23 (5.28 to 7.13) | 7.10 (6.04 to 8.14) | 0.35 (0.21 to 0.48) |
| Croatia | 388 (331 to 445) | 561 (476 to 648) | 8.62 (7.23 to 10.00) | 14.04 (11.45 to 16.93) | 0.85 (0.58 to 1.12) |
| Cuba | 530 (444 to 629) | 704 (586 to 835) | 5.10 (4.29 to 6.00) | 5.69 (4.75 to 6.64) | 0.43 (0.25 to 0.61) |
| Cyprus | 48 (40 to 56) | 83 (69 to 99) | 6.31 (5.31 to 7.32) | 5.78 (4.92 to 6.79) | -0.34 (-0.40 to -0.27) |
| Czechia | 623 (521 to 748) | 952 (810 to 1102) | 5.91 (4.97 to 6.98) | 8.55 (7.12 to 10.01) | 1.03 (0.90 to 1.15) |
| Côte d'Ivoire | 467 (368 to 575) | 1020 (820 to 1255) | 4.52 (3.70 to 5.41) | 4.39 (3.62 to 5.27) | -0.09 (-0.10 to -0.07) |
| Democratic People's Republic of Korea | 1059 (887 to 1234) | 1526 (1311 to 1783) | 5.62 (4.76 to 6.49) | 6.02 (5.12 to 7.01) | 0.28 (0.24 to 0.32) |
| Democratic Republic of the Congo | 1403 (1132 to 1703) | 2848 (2301 to 3454) | 4.37 (3.63 to 5.25) | 4.03 (3.33 to 4.90) | -0.32 (-0.34 to -0.29) |
| Denmark | 277 (229 to 335) | 463 (392 to 540) | 4.79 (4.06 to 5.68) | 7.33 (6.16 to 8.51) | 1.76 (0.83 to 2.70) |
| Djibouti | 17 (13 to 20) | 43 (35 to 52) | 4.74 (3.92 to 5.66) | 4.15 (3.43 to 5.00) | -0.51 (-0.55 to -0.48) |
| Dominica | 5 (4 to 5) | 4 (3 to 5) | 6.37 (5.40 to 7.34) | 6.14 (5.18 to 7.11) | -0.14 (-0.17 to -0.11) |
| Dominican Republic | 254 (208 to 303) | 463 (384 to 557) | 4.24 (3.52 to 5.11) | 4.38 (3.64 to 5.26) | 0.10 (0.06 to 0.14) |
| Ecuador | 395 (334 to 456) | 692 (581 to 833) | 4.70 (4.02 to 5.49) | 4.00 (3.38 to 4.76) | -0.75 (-0.88 to -0.61) |
| Egypt | 4014 (3203 to 4924) | 5160 (4163 to 6096) | 6.61 (5.51 to 7.79) | 5.13 (4.26 to 5.90) | -0.87 (-0.90 to -0.83) |
| El Salvador | 180 (149 to 215) | 258 (214 to 314) | 4.05 (3.37 to 4.90) | 4.01 (3.31 to 4.86) | -0.05 (-0.06 to -0.04) |
| Equatorial Guinea | 16 (13 to 19) | 45 (36 to 56) | 4.36 (3.61 to 5.27) | 3.95 (3.26 to 4.78) | -0.39 (-0.43 to -0.36) |
| Eritrea | 154 (121 to 193) | 217 (175 to 264) | 4.98 (4.13 to 6.02) | 4.13 (3.42 to 5.00) | -0.63 (-0.68 to -0.58) |
| Estonia | 76 (64 to 92) | 73 (59 to 90) | 4.61 (3.89 to 5.46) | 4.15 (3.44 to 4.99) | -0.40 (-0.47 to -0.32) |
| Eswatini | 32 (25 to 38) | 41 (34 to 50) | 4.66 (3.86 to 5.53) | 4.31 (3.58 to 5.15) | -0.21 (-0.25 to -0.17) |
| Ethiopia | 3699 (2911 to 4557) | 4552 (3755 to 5438) | 6.89 (5.73 to 8.11) | 5.02 (4.23 to 6.01) | -1.22 (-1.30 to -1.14) |
| Fiji | 28 (23 to 34) | 39 (32 to 47) | 4.43 (3.71 to 5.33) | 4.56 (3.80 to 5.48) | 0.13 (0.12 to 0.15) |
| Finland | 362 (307 to 417) | 642 (548 to 735) | 7.37 (6.25 to 8.45) | 12.27 (10.11 to 14.62) | 2.11 (1.09 to 3.15) |
| France | 2858 (2330 to 3510) | 5494 (4688 to 6440) | 4.31 (3.55 to 5.19) | 7.11 (6.07 to 8.24) | 2.00 (1.28 to 2.71) |
| Gabon | 37 (30 to 44) | 61 (50 to 74) | 4.28 (3.56 to 5.16) | 3.99 (3.30 to 4.82) | -0.26 (-0.27 to -0.25) |
| Gambia | 41 (32 to 50) | 88 (71 to 106) | 4.68 (3.88 to 5.64) | 4.38 (3.61 to 5.28) | -0.25 (-0.26 to -0.24) |
| Georgia | 374 (319 to 430) | 230 (196 to 269) | 6.85 (5.83 to 7.90) | 5.66 (4.81 to 6.61) | -0.68 (-1.15 to -0.21) |
| Germany | 4950 (4154 to 5878) | 7907 (6713 to 9286) | 5.51 (4.71 to 6.46) | 8.35 (7.12 to 9.65) | 2.00 (1.10 to 2.91) |
| Ghana | 549 (445 to 657) | 1112 (894 to 1352) | 4.36 (3.63 to 5.24) | 3.98 (3.27 to 4.81) | -0.38 (-0.41 to -0.34) |
| Greece | 475 (381 to 587) | 673 (556 to 809) | 3.96 (3.27 to 4.84) | 5.15 (4.37 to 6.05) | 1.16 (0.54 to 1.78) |
| Greenland | 4 (3 to 5) | 5 (4 to 6) | 7.90 (6.65 to 9.15) | 10.25 (8.55 to 12.02) | 1.22 (1.05 to 1.38) |
| Grenada | 5 (4 to 6) | 6 (5 to 7) | 5.82 (4.92 to 6.75) | 6.21 (5.27 to 7.20) | 0.04 (-0.07 to 0.16) |
| Guam | 7 (6 to 8) | 9 (8 to 11) | 5.56 (4.68 to 6.58) | 5.44 (4.56 to 6.44) | -0.10 (-0.20 to 0.00) |
| Guatemala | 390 (325 to 458) | 834 (687 to 977) | 5.05 (4.24 to 5.88) | 5.70 (4.75 to 6.65) | 0.15 (0.06 to 0.24) |
| Guinea | 254 (204 to 310) | 494 (398 to 600) | 4.59 (3.74 to 5.51) | 4.36 (3.59 to 5.28) | -0.18 (-0.19 to -0.17) |
| Guinea-Bissau | 40 (32 to 49) | 71 (57 to 88) | 4.57 (3.78 to 5.49) | 4.27 (3.53 to 5.15) | -0.25 (-0.26 to -0.24) |
| Guyana | 72 (58 to 87) | 83 (69 to 99) | 8.79 (7.27 to 10.29) | 11.44 (9.57 to 13.45) | 1.00 (0.88 to 1.12) |
| Haiti | 344 (281 to 414) | 667 (545 to 793) | 5.56 (4.71 to 6.49) | 5.71 (4.77 to 6.70) | 0.14 (0.11 to 0.17) |
| Honduras | 169 (141 to 201) | 371 (308 to 444) | 4.36 (3.66 to 5.24) | 4.29 (3.56 to 5.18) | -0.05 (-0.06 to -0.03) |
| Hungary | 546 (457 to 657) | 670 (556 to 789) | 4.99 (4.23 to 5.91) | 6.19 (5.25 to 7.27) | 0.52 (0.42 to 0.62) |
| Iceland | 12 (10 to 15) | 26 (22 to 31) | 4.61 (3.84 to 5.52) | 7.02 (5.93 to 8.17) | 1.56 (0.82 to 2.31) |
| India | 42559 (34859 to 50533) | 68387 (56919 to 81965) | 5.50 (4.60 to 6.49) | 5.24 (4.39 to 6.22) | -0.14 (-0.15 to -0.12) |
| Indonesia | 9341 (7669 to 11175) | 14656 (12181 to 17695) | 5.80 (4.85 to 6.91) | 5.82 (4.88 to 6.95) | 0.04 (0.02 to 0.06) |
| Iran (Islamic Republic of) | 1723 (1424 to 2061) | 3164 (2602 to 3829) | 3.81 (3.19 to 4.53) | 3.85 (3.22 to 4.58) | 0.07 (0.03 to 0.11) |
| Iraq | 1219 (1006 to 1457) | 2473 (2075 to 2852) | 6.45 (5.55 to 7.39) | 6.56 (5.56 to 7.47) | 0.09 (0.05 to 0.12) |
| Ireland | 179 (150 to 214) | 557 (477 to 641) | 4.87 (4.11 to 5.73) | 11.78 (9.78 to 14.01) | 3.37 (2.30 to 4.46) |
| Israel | 227 (188 to 270) | 629 (532 to 732) | 4.62 (3.85 to 5.51) | 6.16 (5.21 to 7.21) | 1.18 (0.42 to 1.94) |
| Italy | 6490 (5643 to 7441) | 6141 (5258 to 7077) | 10.83 (9.38 to 12.39) | 7.05 (6.08 to 8.07) | -2.08 (-3.10 to -1.04) |
| Jamaica | 110 (92 to 128) | 155 (131 to 181) | 4.86 (4.10 to 5.71) | 5.61 (4.73 to 6.50) | 0.55 (0.50 to 0.60) |
| Japan | 9307 (7860 to 11069) | 16906 (14491 to 19452) | 7.53 (6.37 to 8.77) | 14.48 (11.93 to 17.55) | 1.47 (1.02 to 1.93) |
| Jordan | 105 (85 to 127) | 385 (314 to 461) | 3.48 (2.90 to 4.13) | 3.59 (2.99 to 4.25) | 0.08 (0.05 to 0.10) |
| Kazakhstan | 717 (598 to 859) | 1451 (1257 to 1673) | 4.69 (3.94 to 5.62) | 7.94 (6.93 to 9.14) | 2.21 (2.08 to 2.35) |
| Kenya | 887 (717 to 1072) | 1752 (1419 to 2137) | 4.78 (3.99 to 5.74) | 4.41 (3.65 to 5.35) | -0.29 (-0.32 to -0.27) |
| Kiribati | 3 (3 to 4) | 6 (5 to 7) | 5.05 (4.27 to 6.01) | 5.26 (4.41 to 6.23) | 0.10 (0.09 to 0.11) |
| Kuwait | 75 (62 to 90) | 271 (225 to 319) | 4.95 (4.19 to 5.73) | 7.48 (6.20 to 8.76) | 1.11 (0.79 to 1.44) |
| Kyrgyzstan | 283 (231 to 333) | 490 (411 to 581) | 6.36 (5.27 to 7.45) | 7.39 (6.30 to 8.65) | 0.93 (0.53 to 1.32) |
| Lao People's Democratic Republic | 169 (139 to 203) | 312 (254 to 375) | 4.79 (4.01 to 5.73) | 4.89 (4.14 to 5.81) | 0.08 (0.08 to 0.09) |
| Latvia | 122 (101 to 148) | 104 (83 to 128) | 4.22 (3.50 to 5.07) | 4.04 (3.34 to 4.88) | -0.25 (-0.30 to -0.21) |
| Lebanon | 145 (123 to 165) | 299 (257 to 344) | 5.12 (4.41 to 5.83) | 5.45 (4.64 to 6.26) | 0.21 (0.16 to 0.26) |
| Lesotho | 59 (49 to 70) | 68 (56 to 83) | 4.45 (3.71 to 5.30) | 4.29 (3.57 to 5.15) | -0.09 (-0.11 to -0.07) |
| Liberia | 102 (81 to 127) | 197 (158 to 241) | 4.62 (3.82 to 5.53) | 4.39 (3.62 to 5.29) | -0.20 (-0.22 to -0.19) |
| Libya | 150 (125 to 175) | 251 (206 to 301) | 4.11 (3.50 to 4.76) | 4.12 (3.46 to 4.83) | 0.04 (0.02 to 0.06) |
| Lithuania | 169 (141 to 206) | 155 (124 to 191) | 4.35 (3.63 to 5.26) | 4.14 (3.43 to 5.04) | -0.21 (-0.25 to -0.18) |
| Luxembourg | 22 (18 to 26) | 59 (50 to 69) | 5.20 (4.41 to 6.24) | 9.68 (7.99 to 11.48) | 2.23 (1.72 to 2.74) |
| Madagascar | 536 (422 to 665) | 946 (763 to 1144) | 4.93 (4.08 to 5.87) | 4.20 (3.49 to 5.07) | -0.52 (-0.55 to -0.50) |
| Malawi | 457 (352 to 578) | 617 (496 to 753) | 4.97 (4.09 to 5.92) | 4.08 (3.40 to 4.95) | -0.73 (-0.79 to -0.68) |
| Malaysia | 1019 (849 to 1195) | 1775 (1474 to 2093) | 6.14 (5.20 to 7.11) | 6.11 (5.13 to 7.13) | 0.02 (-0.03 to 0.07) |
| Maldives | 12 (10 to 14) | 25 (21 to 30) | 5.78 (4.89 to 6.77) | 5.78 (4.91 to 6.81) | 0.08 (0.05 to 0.12) |
| Mali | 359 (284 to 441) | 903 (725 to 1107) | 4.62 (3.78 to 5.58) | 4.44 (3.64 to 5.37) | -0.15 (-0.16 to -0.14) |
| Malta | 23 (20 to 27) | 64 (55 to 75) | 6.37 (5.48 to 7.35) | 15.73 (12.96 to 19.12) | 3.16 (1.89 to 4.45) |
| Marshall Islands | 2 (1 to 2) | 2 (2 to 3) | 4.38 (3.66 to 5.27) | 4.41 (3.68 to 5.31) | 0.03 (0.02 to 0.04) |
| Mauritania | 84 (67 to 102) | 164 (134 to 198) | 4.56 (3.73 to 5.48) | 4.36 (3.57 to 5.26) | -0.17 (-0.17 to -0.16) |
| Mauritius | 40 (32 to 49) | 67 (55 to 81) | 4.23 (3.50 to 5.17) | 4.84 (4.10 to 5.77) | 0.52 (0.40 to 0.64) |
| Mexico | 3073 (2525 to 3719) | 6269 (5241 to 7540) | 4.47 (3.70 to 5.44) | 4.98 (4.20 to 5.97) | 0.55 (0.50 to 0.61) |
| Micronesia (Federated States of) | 4 (3 to 5) | 4 (3 to 5) | 4.39 (3.67 to 5.28) | 4.39 (3.66 to 5.28) | 0.01 (0.01 to 0.01) |
| Monaco | 2 (2 to 3) | 3 (2 to 3) | 5.90 (5.06 to 6.76) | 5.34 (4.51 to 6.22) | -0.34 (-0.38 to -0.31) |
| Mongolia | 153 (126 to 181) | 193 (163 to 227) | 7.33 (6.23 to 8.42) | 6.30 (5.39 to 7.30) | -0.55 (-0.71 to -0.40) |
| Montenegro | 51 (44 to 59) | 44 (37 to 51) | 8.54 (7.32 to 9.82) | 7.03 (6.00 to 7.99) | -0.73 (-0.84 to -0.62) |
| Morocco | 891 (744 to 1042) | 1434 (1196 to 1691) | 3.98 (3.37 to 4.63) | 4.05 (3.41 to 4.71) | 0.09 (0.07 to 0.10) |
| Mozambique | 539 (430 to 659) | 919 (741 to 1122) | 4.56 (3.76 to 5.46) | 3.94 (3.25 to 4.76) | -0.52 (-0.57 to -0.48) |
| Myanmar | 1688 (1388 to 2033) | 2638 (2198 to 3150) | 4.88 (4.09 to 5.82) | 5.04 (4.27 to 5.99) | 0.14 (0.13 to 0.15) |
| Namibia | 55 (45 to 67) | 91 (75 to 110) | 4.62 (3.83 to 5.51) | 4.37 (3.63 to 5.23) | -0.13 (-0.17 to -0.10) |
| Nauru | 0 (0 to 0) | 0 (0 to 0) | 4.47 (3.75 to 5.37) | 4.39 (3.66 to 5.29) | -0.06 (-0.07 to -0.06) |
| Nepal | 1017 (831 to 1204) | 1444 (1195 to 1698) | 5.36 (4.51 to 6.24) | 5.07 (4.27 to 5.93) | -0.16 (-0.17 to -0.14) |
| Netherlands | 736 (602 to 901) | 1365 (1161 to 1603) | 4.49 (3.72 to 5.38) | 7.16 (6.03 to 8.27) | 1.93 (0.98 to 2.89) |
| New Zealand | 363 (304 to 422) | 732 (611 to 874) | 11.30 (9.32 to 13.40) | 16.42 (13.16 to 20.28) | 0.94 (0.09 to 1.79) |
| Nicaragua | 141 (117 to 168) | 259 (214 to 309) | 4.37 (3.66 to 5.24) | 4.31 (3.59 to 5.18) | -0.04 (-0.07 to -0.01) |
| Niger | 335 (260 to 421) | 939 (739 to 1158) | 4.70 (3.85 to 5.65) | 4.45 (3.65 to 5.37) | -0.20 (-0.20 to -0.19) |
| Nigeria | 5460 (4314 to 6735) | 11733 (9521 to 14307) | 6.08 (5.04 to 7.21) | 5.53 (4.62 to 6.56) | -0.39 (-0.41 to -0.37) |
| Niue | 0 (0 to 0) | 0 (0 to 0) | 4.44 (3.72 to 5.34) | 4.48 (3.73 to 5.37) | 0.02 (0.01 to 0.04) |
| North Macedonia | 105 (89 to 123) | 123 (102 to 148) | 5.49 (4.69 to 6.45) | 5.54 (4.72 to 6.49) | -0.02 (-0.08 to 0.03) |
| Northern Mariana Islands | 2 (1 to 2) | 2 (2 to 3) | 4.93 (4.14 to 5.85) | 5.21 (4.36 to 6.20) | 0.23 (0.20 to 0.25) |
| Norway | 330 (279 to 381) | 471 (399 to 545) | 7.80 (6.57 to 9.04) | 8.52 (7.14 to 9.96) | 0.73 (0.24 to 1.23) |
| Oman | 98 (81 to 117) | 200 (163 to 239) | 5.21 (4.48 to 5.97) | 5.03 (4.27 to 5.80) | -0.09 (-0.22 to 0.05) |
| Pakistan | 6171 (5070 to 7359) | 11371 (9316 to 13583) | 5.73 (4.78 to 6.73) | 5.44 (4.55 to 6.46) | -0.14 (-0.16 to -0.12) |
| Palau | 1 (1 to 1) | 1 (1 to 1) | 5.06 (4.24 to 6.04) | 5.11 (4.28 to 6.06) | 0.04 (0.01 to 0.07) |
| Palestine | 66 (55 to 79) | 167 (138 to 198) | 3.66 (3.07 to 4.32) | 3.78 (3.17 to 4.42) | 0.12 (0.09 to 0.15) |
| Panama | 87 (72 to 104) | 211 (178 to 250) | 4.27 (3.57 to 5.14) | 4.86 (4.10 to 5.75) | 0.35 (0.27 to 0.42) |
| Papua New Guinea | 148 (120 to 178) | 383 (312 to 462) | 4.43 (3.71 to 5.32) | 4.44 (3.70 to 5.38) | 0.01 (0.01 to 0.02) |
| Paraguay | 162 (136 to 192) | 294 (243 to 352) | 4.54 (3.79 to 5.42) | 4.44 (3.71 to 5.32) | -0.09 (-0.11 to -0.08) |
| Peru | 796 (667 to 940) | 1464 (1211 to 1756) | 4.46 (3.75 to 5.29) | 4.16 (3.45 to 4.99) | -0.32 (-0.37 to -0.26) |
| Philippines | 2870 (2357 to 3442) | 5277 (4339 to 6366) | 5.35 (4.47 to 6.44) | 5.20 (4.35 to 6.26) | -0.06 (-0.08 to -0.04) |
| Poland | 3526 (2975 to 4094) | 3936 (3357 to 4554) | 9.61 (8.01 to 11.26) | 10.37 (8.59 to 12.33) | -0.09 (-0.27 to 0.09) |
| Portugal | 469 (380 to 574) | 765 (633 to 910) | 4.18 (3.47 to 5.07) | 5.79 (4.86 to 6.81) | 1.34 (0.65 to 2.02) |
| Puerto Rico | 148 (123 to 180) | 224 (183 to 270) | 4.12 (3.45 to 4.98) | 5.28 (4.40 to 6.22) | 0.75 (0.42 to 1.08) |
| Qatar | 16 (13 to 19) | 112 (90 to 138) | 4.30 (3.64 to 4.98) | 4.78 (4.07 to 5.52) | 0.43 (0.36 to 0.50) |
| Republic of Korea | 3862 (3181 to 4608) | 5003 (4288 to 5796) | 9.52 (7.89 to 11.28) | 11.46 (9.44 to 13.57) | 0.86 (0.74 to 0.99) |
| Republic of Moldova | 220 (185 to 257) | 191 (156 to 230) | 5.09 (4.31 to 5.93) | 4.66 (3.92 to 5.53) | -0.33 (-0.43 to -0.24) |
| Romania | 2477 (2091 to 2903) | 3302 (2823 to 3874) | 11.23 (9.39 to 13.34) | 17.75 (14.88 to 21.28) | 1.40 (1.23 to 1.57) |
| Russian Federation | 11205 (9477 to 13065) | 9762 (8235 to 11661) | 7.80 (6.56 to 9.04) | 6.27 (5.30 to 7.38) | -0.76 (-0.89 to -0.64) |
| Rwanda | 316 (248 to 392) | 440 (358 to 537) | 4.82 (3.99 to 5.77) | 4.09 (3.39 to 4.93) | -0.76 (-0.84 to -0.68) |
| Saint Kitts and Nevis | 2 (1 to 2) | 3 (2 to 3) | 4.09 (3.41 to 4.94) | 4.24 (3.53 to 5.13) | 0.20 (0.02 to 0.37) |
| Saint Lucia | 11 (9 to 13) | 14 (12 to 16) | 7.68 (6.50 to 8.86) | 8.33 (7.05 to 9.59) | 0.14 (-0.02 to 0.31) |
| Saint Vincent and the Grenadines | 5 (4 to 6) | 7 (6 to 9) | 4.80 (4.02 to 5.68) | 6.51 (5.46 to 7.64) | 0.65 (0.32 to 0.98) |
| Samoa | 6 (5 to 8) | 9 (7 to 10) | 4.48 (3.76 to 5.38) | 4.47 (3.71 to 5.37) | -0.02 (-0.03 to -0.01) |
| San Marino | 2 (2 to 2) | 3 (3 to 4) | 8.14 (7.01 to 9.32) | 9.84 (8.26 to 11.58) | 0.89 (0.65 to 1.14) |
| Sao Tome and Principe | 5 (4 to 6) | 8 (7 to 10) | 4.58 (3.79 to 5.53) | 4.40 (3.63 to 5.28) | -0.20 (-0.23 to -0.18) |
| Saudi Arabia | 618 (508 to 727) | 1236 (987 to 1517) | 4.37 (3.68 to 5.06) | 3.93 (3.29 to 4.60) | -0.44 (-0.48 to -0.40) |
| Senegal | 262 (213 to 319) | 517 (419 to 630) | 4.17 (3.44 to 5.07) | 4.00 (3.30 to 4.84) | -0.16 (-0.17 to -0.15) |
| Serbia | 509 (428 to 606) | 527 (433 to 637) | 5.43 (4.57 to 6.34) | 5.03 (4.23 to 5.92) | -0.38 (-0.45 to -0.31) |
| Seychelles | 5 (4 to 5) | 6 (5 to 7) | 6.35 (5.39 to 7.29) | 6.18 (5.25 to 7.16) | 0.01 (-0.09 to 0.12) |
| Sierra Leone | 170 (136 to 208) | 324 (263 to 393) | 4.55 (3.77 to 5.51) | 4.36 (3.59 to 5.26) | -0.17 (-0.19 to -0.15) |
| Singapore | 500 (415 to 600) | 1226 (1021 to 1457) | 18.54 (15.25 to 22.57) | 27.84 (22.49 to 34.72) | 0.51 (0.24 to 0.78) |
| Slovakia | 310 (262 to 366) | 371 (312 to 442) | 5.84 (4.96 to 6.83) | 6.31 (5.38 to 7.40) | 0.22 (0.19 to 0.24) |
| Slovenia | 87 (73 to 106) | 130 (107 to 155) | 4.14 (3.46 to 4.95) | 4.88 (4.16 to 5.73) | 0.39 (0.32 to 0.47) |
| Solomon Islands | 12 (9 to 14) | 24 (20 to 29) | 4.35 (3.64 to 5.25) | 4.32 (3.60 to 5.23) | -0.03 (-0.04 to -0.03) |
| Somalia | 357 (276 to 451) | 721 (575 to 872) | 4.97 (4.09 to 5.93) | 4.26 (3.54 to 5.16) | -0.57 (-0.60 to -0.54) |
| South Africa | 1602 (1318 to 1919) | 2354 (1935 to 2876) | 4.94 (4.12 to 5.89) | 4.45 (3.69 to 5.38) | -0.36 (-0.41 to -0.30) |
| South Sudan | 279 (217 to 350) | 355 (286 to 430) | 5.09 (4.17 to 6.13) | 4.45 (3.67 to 5.35) | -0.47 (-0.55 to -0.39) |
| Spain | 1739 (1417 to 2130) | 3425 (2908 to 4009) | 3.97 (3.30 to 4.77) | 6.32 (5.34 to 7.32) | 1.52 (0.87 to 2.16) |
| Sri Lanka | 1986 (1614 to 2401) | 1870 (1579 to 2185) | 12.57 (10.53 to 14.67) | 8.75 (7.41 to 10.22) | -1.82 (-2.03 to -1.60) |
| Sudan | 610 (506 to 723) | 1264 (1032 to 1510) | 3.68 (3.09 to 4.33) | 3.62 (3.02 to 4.29) | -0.05 (-0.06 to -0.04) |
| Suriname | 17 (14 to 20) | 28 (23 to 33) | 4.85 (4.07 to 5.71) | 4.84 (4.05 to 5.71) | 0.07 (0.04 to 0.10) |
| Sweden | 1728 (1505 to 1962) | 2056 (1787 to 2384) | 21.81 (18.66 to 25.43) | 20.11 (16.76 to 23.71) | -0.56 (-0.92 to -0.20) |
| Switzerland | 307 (250 to 371) | 525 (439 to 620) | 3.85 (3.21 to 4.58) | 4.88 (4.12 to 5.62) | 1.04 (0.44 to 1.66) |
| Syrian Arab Republic | 390 (326 to 466) | 521 (435 to 625) | 3.76 (3.18 to 4.42) | 3.90 (3.32 to 4.57) | 0.07 (0.06 to 0.09) |
| Taiwan (Province of China) | 879 (740 to 1040) | 2098 (1829 to 2405) | 4.79 (4.07 to 5.62) | 9.22 (7.76 to 10.95) | 3.04 (2.66 to 3.42) |
| Tajikistan | 177 (142 to 218) | 350 (283 to 429) | 4.10 (3.35 to 5.02) | 4.13 (3.37 to 5.09) | 0.03 (0.03 to 0.03) |
| Thailand | 2648 (2178 to 3158) | 4392 (3712 to 5208) | 5.36 (4.56 to 6.33) | 5.97 (5.07 to 6.99) | 0.39 (0.35 to 0.43) |
| Timor-Leste | 33 (27 to 39) | 63 (52 to 75) | 5.05 (4.24 to 6.02) | 5.09 (4.31 to 6.04) | 0.05 (0.04 to 0.06) |
| Togo | 144 (114 to 180) | 306 (248 to 374) | 4.57 (3.77 to 5.47) | 4.34 (3.58 to 5.23) | -0.18 (-0.19 to -0.17) |
| Tokelau | 0 (0 to 0) | 0 (0 to 0) | 4.47 (3.77 to 5.38) | 4.49 (3.74 to 5.38) | 0.03 (0.02 to 0.03) |
| Tonga | 4 (3 to 5) | 5 (4 to 5) | 4.61 (3.86 to 5.51) | 4.58 (3.81 to 5.47) | -0.02 (-0.03 to -0.02) |
| Trinidad and Tobago | 86 (72 to 102) | 111 (94 to 129) | 7.27 (6.13 to 8.41) | 8.17 (6.83 to 9.46) | 0.46 (0.39 to 0.53) |
| Tunisia | 276 (232 to 326) | 476 (397 to 565) | 3.88 (3.28 to 4.53) | 3.94 (3.31 to 4.59) | 0.03 (0.01 to 0.04) |
| Turkmenistan | 207 (174 to 243) | 327 (280 to 381) | 5.99 (5.11 to 6.99) | 6.73 (5.76 to 7.76) | 0.53 (0.20 to 0.86) |
| Tuvalu | 0 (0 to 0) | 1 (0 to 1) | 4.33 (3.64 to 5.22) | 4.40 (3.66 to 5.31) | 0.06 (0.06 to 0.06) |
| Türkiye | 1655 (1353 to 2000) | 3001 (2489 to 3647) | 3.35 (2.78 to 4.07) | 3.48 (2.91 to 4.18) | 0.14 (0.11 to 0.16) |
| Uganda | 676 (535 to 831) | 1293 (1038 to 1575) | 4.49 (3.73 to 5.39) | 3.96 (3.26 to 4.78) | -0.45 (-0.49 to -0.42) |
| Ukraine | 3239 (2726 to 3835) | 2928 (2454 to 3507) | 6.04 (5.09 to 7.10) | 6.29 (5.27 to 7.49) | 0.22 (0.13 to 0.32) |
| United Arab Emirates | 86 (71 to 103) | 402 (321 to 495) | 5.36 (4.59 to 6.08) | 5.23 (4.45 to 6.01) | -0.04 (-0.07 to -0.02) |
| United Kingdom | 6390 (5445 to 7286) | 7189 (6164 to 8265) | 11.65 (9.72 to 13.61) | 10.66 (8.92 to 12.47) | 0.38 (0.03 to 0.74) |
| United Republic of Tanzania | 1148 (901 to 1420) | 1939 (1580 to 2330) | 4.86 (4.05 to 5.78) | 4.13 (3.42 to 4.99) | -0.58 (-0.63 to -0.54) |
| United States Virgin Islands | 8 (7 to 10) | 8 (7 to 10) | 8.22 (6.96 to 9.50) | 9.14 (7.74 to 10.60) | 0.37 (0.17 to 0.57) |
| United States of America | 33821 (28559 to 39605) | 44701 (38110 to 52164) | 14.34 (11.88 to 17.06) | 15.80 (12.90 to 19.18) | 0.29 (-0.01 to 0.59) |
| Uruguay | 195 (166 to 226) | 218 (184 to 255) | 6.00 (5.11 to 6.91) | 5.64 (4.78 to 6.57) | -0.39 (-0.57 to -0.20) |
| Uzbekistan | 1157 (975 to 1358) | 1528 (1267 to 1830) | 5.76 (4.91 to 6.74) | 4.85 (4.09 to 5.77) | -0.48 (-0.63 to -0.33) |
| Vanuatu | 5 (4 to 6) | 11 (9 to 14) | 4.38 (3.66 to 5.26) | 4.34 (3.60 to 5.24) | -0.02 (-0.03 to -0.02) |
| Venezuela (Bolivarian Republic of) | 751 (623 to 884) | 1266 (1056 to 1509) | 4.60 (3.86 to 5.45) | 4.70 (3.93 to 5.56) | 0.01 (-0.02 to 0.05) |
| Viet Nam | 3518 (2909 to 4115) | 5667 (4775 to 6730) | 5.56 (4.70 to 6.51) | 6.01 (5.07 to 7.00) | 0.28 (0.27 to 0.29) |
| Yemen | 385 (317 to 459) | 968 (794 to 1164) | 3.63 (3.07 to 4.28) | 3.65 (3.05 to 4.30) | 0.04 (0.03 to 0.05) |
| Zambia | 360 (276 to 449) | 612 (493 to 746) | 4.96 (4.06 to 5.92) | 4.10 (3.39 to 4.93) | -0.74 (-0.79 to -0.69) |
| Zimbabwe | 516 (419 to 612) | 755 (604 to 910) | 5.54 (4.66 to 6.48) | 5.46 (4.56 to 6.42) | 0.07 (0.02 to 0.11) |

# Table S5. The death cases and age-standardized death rate of myocarditis in 1990 and 2021, and EAPC in age-standardized death rate from 1990 to 2021

| **Characteristics** | **Death cases (95% UI)** | | **ASDR (95% UI)** | | **EAPC in ASDR**  **(95% CI)** |
| --- | --- | --- | --- | --- | --- |
|  | **1990** | **2021** | **1990** | **2021** | **1990-2021** |
| **Global** | 21765 (17945 to 25869) | 31765 (25490 to 37053) | 0.56 (0.46 to 0.65) | 0.40 (0.32 to 0.47) | -1.37 (-1.81 to -0.92) |
| **Gender** |  |  |  |  |  |
| Male | 11133 (8824 to 14495) | 16988 (12785 to 21077) | 0.60 (0.48 to 0.78) | 0.48 (0.36 to 0.59) | -1.07 (-1.45 to -0.68) |
| Female | 10633 (8275 to 12763) | 14776 (11384 to 17758) | 0.51 (0.40 to 0.62) | 0.33 (0.26 to 0.40) | -1.64 (-2.15 to -1.12) |
| **SDI** |  |  |  |  |  |
| High-middle SDI | 7314 (6202 to 8328) | 10062 (8393 to 11529) | 1.05 (0.88 to 1.20) | 0.58 (0.48 to 0.66) | -2.59 (-3.36 to -1.81) |
| High SDI | 2309 (2133 to 2465) | 3222 (2889 to 3487) | 0.25 (0.23 to 0.27) | 0.20 (0.18 to 0.21) | -0.99 (-1.53 to -0.45) |
| Low-middle SDI | 2809 (1602 to 4553) | 4037 (2795 to 6321) | 0.37 (0.20 to 0.58) | 0.29 (0.20 to 0.46) | -0.63 (-0.71 to -0.56) |
| Low SDI | 1405 (753 to 2264) | 1810 (1129 to 3208) | 0.37 (0.17 to 0.65) | 0.26 (0.14 to 0.49) | -1.04 (-1.18 to -0.91) |
| Middle SDI | 7901 (6107 to 9644) | 12585 (8494 to 15852) | 0.69 (0.53 to 0.85) | 0.58 (0.39 to 0.74) | -0.65 (-0.99 to -0.32) |
| **Region** |  |  |  |  |  |
| East Asia | 8501 (6484 to 11032) | 14612 (9959 to 18901) | 1.09 (0.83 to 1.40) | 0.91 (0.63 to 1.18) | -0.66 (-1.12 to -0.19) |
| Southeast Asia | 1080 (767 to 1698) | 1439 (1113 to 2154) | 0.45 (0.30 to 0.68) | 0.28 (0.22 to 0.40) | -1.97 (-2.21 to -1.73) |
| Oceania | 15 (8 to 25) | 31 (17 to 53) | 0.22 (0.12 to 0.36) | 0.21 (0.11 to 0.37) | 0.03 (-0.08 to 0.13) |
| Central Asia | 222 (186 to 270) | 720 (573 to 910) | 0.44 (0.35 to 0.54) | 0.84 (0.68 to 1.05) | 2.86 (2.10 to 3.64) |
| Central Europe | 1202 (964 to 1517) | 2557 (2078 to 3126) | 0.98 (0.80 to 1.20) | 1.24 (0.99 to 1.52) | -0.25 (-0.82 to 0.32) |
| Eastern Europe | 930 (829 to 1026) | 1392 (1216 to 1582) | 0.47 (0.42 to 0.52) | 0.45 (0.39 to 0.51) | -0.39 (-0.95 to 0.17) |
| High-income Asia Pacific | 388 (343 to 442) | 556 (470 to 623) | 0.24 (0.21 to 0.27) | 0.17 (0.15 to 0.18) | -1.66 (-2.07 to -1.24) |
| Australasia | 103 (92 to 114) | 74 (66 to 82) | 0.51 (0.45 to 0.56) | 0.18 (0.16 to 0.20) | -3.71 (-4.34 to -3.07) |
| Western Europe | 3166 (2559 to 3635) | 2065 (1654 to 2336) | 0.61 (0.50 to 0.70) | 0.20 (0.17 to 0.23) | -4.30 (-5.63 to -2.95) |
| Southern Latin America | 109 (94 to 126) | 85 (76 to 95) | 0.24 (0.21 to 0.28) | 0.10 (0.09 to 0.12) | -2.77 (-3.34 to -2.19) |
| High-income North America | 776 (721 to 824) | 821 (770 to 866) | 0.27 (0.26 to 0.29) | 0.20 (0.19 to 0.21) | -0.99 (-1.42 to -0.56) |
| Caribbean | 81 (55 to 137) | 136 (98 to 188) | 0.25 (0.17 to 0.39) | 0.28 (0.20 to 0.40) | 0.63 (0.56 to 0.70) |
| Andean Latin America | 54 (33 to 79) | 36 (26 to 47) | 0.23 (0.13 to 0.32) | 0.06 (0.04 to 0.08) | -4.74 (-5.02 to -4.46) |
| Central Latin America | 88 (82 to 96) | 291 (256 to 329) | 0.07 (0.06 to 0.08) | 0.12 (0.11 to 0.14) | 2.23 (1.94 to 2.51) |
| Tropical Latin America | 212 (198 to 230) | 408 (367 to 438) | 0.20 (0.18 to 0.21) | 0.17 (0.16 to 0.19) | -0.84 (-1.68 to 0.01) |
| North Africa and Middle East | 886 (406 to 1889) | 1185 (656 to 2659) | 0.45 (0.20 to 1.05) | 0.29 (0.16 to 0.66) | -1.43 (-1.52 to -1.33) |
| South Asia | 2707 (1447 to 4089) | 4114 (2703 to 6129) | 0.36 (0.18 to 0.53) | 0.29 (0.19 to 0.43) | -0.44 (-0.57 to -0.30) |
| Central Sub-Saharan Africa | 165 (84 to 265) | 219 (113 to 481) | 0.44 (0.21 to 0.82) | 0.29 (0.13 to 0.75) | -1.37 (-1.45 to -1.29) |
| Eastern Sub-Saharan Africa | 405 (199 to 710) | 354 (205 to 666) | 0.21 (0.10 to 0.35) | 0.11 (0.06 to 0.23) | -2.32 (-2.44 to -2.19) |
| Southern Sub-Saharan Africa | 97 (61 to 125) | 102 (75 to 167) | 0.26 (0.16 to 0.37) | 0.16 (0.12 to 0.25) | -2.12 (-2.31 to -1.93) |
| Western Sub-Saharan Africa | 577 (355 to 1001) | 566 (368 to 994) | 0.38 (0.18 to 0.69) | 0.14 (0.08 to 0.28) | -3.62 (-3.90 to -3.34) |

# Table S6. The death cases and age-standardized death rate of myocarditis in 1990 and 2021, and EAPC in age-standardized death rate from 1990 to 2021 in 204 countries and territories

| **Characteristics** | **Death cases (95% UI)** | | **ASDR (95% UI)** | | **EAPC in ASDR (95% CI)** |
| --- | --- | --- | --- | --- | --- |
|  | **1990** | **2021** | **1990** | **2021** | **1990-2021** |
| Afghanistan | 56 (7 to 193) | 79 (11 to 266) | 0.78 (0.09 to 2.84) | 0.69 (0.09 to 2.44) | -0.48 (-0.66 to -0.30) |
| Albania | 22 (14 to 34) | 28 (16 to 46) | 1.08 (0.68 to 1.89) | 0.77 (0.47 to 1.25) | -1.33 (-1.58 to -1.07) |
| Algeria | 68 (13 to 205) | 111 (21 to 388) | 0.56 (0.09 to 1.80) | 0.37 (0.07 to 1.29) | -0.85 (-1.03 to -0.67) |
| American Samoa | 0 (0 to 0) | 0 (0 to 0) | 0.03 (0.02 to 0.05) | 0.03 (0.02 to 0.04) | -0.46 (-0.67 to -0.25) |
| Andorra | 0 (0 to 0) | 0 (0 to 1) | 0.61 (0.35 to 1.05) | 0.26 (0.16 to 0.40) | -2.88 (-3.05 to -2.71) |
| Angola | 32 (15 to 61) | 47 (26 to 93) | 0.44 (0.21 to 0.80) | 0.25 (0.12 to 0.60) | -1.91 (-2.02 to -1.80) |
| Antigua and Barbuda | 0 (0 to 0) | 0 (0 to 1) | 0.60 (0.52 to 0.68) | 0.55 (0.50 to 0.61) | 0.01 (-0.23 to 0.26) |
| Argentina | 87 (73 to 102) | 64 (56 to 71) | 0.29 (0.24 to 0.34) | 0.12 (0.11 to 0.13) | -2.85 (-3.39 to -2.30) |
| Armenia | 4 (3 to 6) | 3 (2 to 5) | 0.16 (0.11 to 0.24) | 0.08 (0.05 to 0.12) | -2.89 (-4.07 to -1.69) |
| Australia | 90 (79 to 100) | 55 (48 to 63) | 0.53 (0.47 to 0.59) | 0.16 (0.14 to 0.18) | -4.38 (-5.01 to -3.74) |
| Austria | 5 (4 to 5) | 31 (25 to 36) | 0.05 (0.04 to 0.05) | 0.17 (0.14 to 0.19) | 4.54 (2.14 to 6.99) |
| Azerbaijan | 51 (25 to 89) | 91 (43 to 179) | 0.94 (0.46 to 1.67) | 0.82 (0.41 to 1.53) | -1.48 (-1.93 to -1.03) |
| Bahamas | 0 (0 to 0) | 1 (0 to 1) | 0.16 (0.14 to 0.18) | 0.16 (0.13 to 0.21) | 0.31 (0.00 to 0.62) |
| Bahrain | 0 (0 to 0) | 1 (0 to 1) | 0.23 (0.12 to 0.35) | 0.10 (0.07 to 0.14) | -3.00 (-3.19 to -2.82) |
| Bangladesh | 322 (139 to 532) | 454 (252 to 817) | 0.54 (0.22 to 0.98) | 0.35 (0.20 to 0.62) | -1.20 (-1.36 to -1.03) |
| Barbados | 1 (0 to 1) | 1 (1 to 1) | 0.20 (0.17 to 0.24) | 0.26 (0.21 to 0.32) | 1.10 (0.94 to 1.27) |
| Belarus | 42 (24 to 62) | 18 (14 to 22) | 0.37 (0.23 to 0.54) | 0.12 (0.10 to 0.16) | -4.22 (-4.89 to -3.55) |
| Belgium | 7 (6 to 9) | 41 (31 to 50) | 0.06 (0.05 to 0.07) | 0.14 (0.11 to 0.17) | 3.52 (1.40 to 5.69) |
| Belize | 0 (0 to 0) | 0 (0 to 0) | 0.21 (0.16 to 0.27) | 0.11 (0.10 to 0.13) | -1.64 (-2.33 to -0.94) |
| Benin | 12 (7 to 22) | 14 (8 to 27) | 0.30 (0.14 to 0.54) | 0.13 (0.07 to 0.27) | -2.93 (-3.14 to -2.71) |
| Bermuda | 0 (0 to 0) | 0 (0 to 0) | 0.16 (0.14 to 0.20) | 0.15 (0.13 to 0.19) | -0.38 (-0.91 to 0.14) |
| Bhutan | 1 (1 to 2) | 2 (1 to 4) | 0.44 (0.19 to 0.88) | 0.34 (0.18 to 0.64) | -1.00 (-1.12 to -0.88) |
| Bolivia (Plurinational State of) | 9 (5 to 18) | 10 (6 to 16) | 0.25 (0.13 to 0.45) | 0.12 (0.07 to 0.21) | -2.35 (-2.47 to -2.22) |
| Bosnia and Herzegovina | 34 (17 to 63) | 38 (18 to 73) | 1.02 (0.46 to 2.05) | 0.69 (0.33 to 1.31) | -1.75 (-2.01 to -1.49) |
| Botswana | 3 (2 to 4) | 3 (1 to 6) | 0.31 (0.19 to 0.50) | 0.16 (0.08 to 0.31) | -2.32 (-2.43 to -2.22) |
| Brazil | 209 (195 to 226) | 403 (362 to 433) | 0.20 (0.19 to 0.21) | 0.18 (0.16 to 0.19) | -0.84 (-1.69 to 0.02) |
| Brunei Darussalam | 2 (1 to 2) | 2 (1 to 3) | 0.78 (0.53 to 1.12) | 0.48 (0.35 to 0.66) | -1.36 (-1.52 to -1.21) |
| Bulgaria | 62 (49 to 77) | 153 (116 to 197) | 0.78 (0.63 to 0.94) | 1.30 (0.99 to 1.67) | 0.04 (-0.92 to 1.00) |
| Burkina Faso | 30 (16 to 57) | 35 (20 to 66) | 0.43 (0.19 to 0.88) | 0.20 (0.09 to 0.43) | -2.54 (-2.75 to -2.32) |
| Burundi | 16 (7 to 31) | 13 (6 to 25) | 0.28 (0.13 to 0.50) | 0.13 (0.06 to 0.31) | -2.50 (-2.62 to -2.39) |
| Cabo Verde | 0 (0 to 1) | 0 (0 to 1) | 0.12 (0.05 to 0.25) | 0.06 (0.02 to 0.12) | -2.84 (-2.99 to -2.70) |
| Cambodia | 18 (8 to 39) | 29 (16 to 49) | 0.38 (0.17 to 0.70) | 0.32 (0.19 to 0.52) | -0.78 (-0.91 to -0.65) |
| Cameroon | 26 (15 to 43) | 35 (21 to 65) | 0.33 (0.16 to 0.54) | 0.14 (0.07 to 0.27) | -3.15 (-3.37 to -2.93) |
| Canada | 29 (27 to 32) | 65 (59 to 72) | 0.11 (0.10 to 0.12) | 0.15 (0.13 to 0.16) | 1.23 (0.30 to 2.16) |
| Central African Republic | 10 (5 to 17) | 14 (6 to 28) | 0.54 (0.24 to 0.94) | 0.40 (0.16 to 1.01) | -1.07 (-1.22 to -0.93) |
| Chad | 18 (9 to 35) | 26 (12 to 58) | 0.37 (0.16 to 0.77) | 0.18 (0.08 to 0.40) | -2.66 (-2.87 to -2.44) |
| Chile | 14 (13 to 15) | 13 (12 to 15) | 0.12 (0.11 to 0.13) | 0.06 (0.05 to 0.06) | -1.95 (-2.79 to -1.10) |
| China | 8340 (6332 to 10869) | 14195 (9558 to 18543) | 1.10 (0.84 to 1.44) | 0.92 (0.63 to 1.21) | -0.67 (-1.15 to -0.19) |
| Colombia | 22 (20 to 25) | 75 (62 to 90) | 0.08 (0.07 to 0.09) | 0.14 (0.12 to 0.17) | 1.78 (1.27 to 2.29) |
| Comoros | 1 (0 to 2) | 1 (0 to 1) | 0.22 (0.10 to 0.35) | 0.10 (0.04 to 0.21) | -3.00 (-3.28 to -2.71) |
| Congo | 6 (3 to 9) | 7 (4 to 15) | 0.38 (0.19 to 0.62) | 0.20 (0.11 to 0.47) | -2.23 (-2.34 to -2.12) |
| Cook Islands | 0 (0 to 0) | 0 (0 to 0) | 0.00 (0.00 to 0.01) | 0.00 (0.00 to 0.00) | -3.48 (-3.72 to -3.24) |
| Costa Rica | 6 (5 to 7) | 16 (13 to 18) | 0.29 (0.24 to 0.34) | 0.28 (0.24 to 0.32) | -0.25 (-0.67 to 0.17) |
| Croatia | 62 (52 to 74) | 143 (111 to 181) | 1.32 (1.10 to 1.56) | 1.62 (1.23 to 2.07) | -2.24 (-3.91 to -0.54) |
| Cuba | 12 (11 to 13) | 14 (12 to 16) | 0.12 (0.11 to 0.14) | 0.09 (0.08 to 0.11) | -1.11 (-1.43 to -0.80) |
| Cyprus | 2 (1 to 3) | 1 (1 to 2) | 0.37 (0.16 to 0.62) | 0.09 (0.06 to 0.14) | -5.24 (-5.74 to -4.73) |
| Czechia | 22 (19 to 26) | 51 (38 to 68) | 0.19 (0.16 to 0.22) | 0.26 (0.19 to 0.35) | 0.58 (0.11 to 1.05) |
| Côte d'Ivoire | 30 (18 to 53) | 33 (18 to 59) | 0.33 (0.16 to 0.59) | 0.15 (0.08 to 0.28) | -3.02 (-3.30 to -2.75) |
| Democratic People's Republic of Korea | 134 (89 to 200) | 245 (149 to 460) | 1.10 (0.66 to 1.74) | 1.05 (0.60 to 2.01) | 0.09 (-0.16 to 0.34) |
| Democratic Republic of the Congo | 114 (58 to 186) | 148 (71 to 347) | 0.43 (0.21 to 0.83) | 0.30 (0.12 to 0.84) | -1.12 (-1.21 to -1.03) |
| Denmark | 6 (5 to 7) | 17 (14 to 20) | 0.09 (0.08 to 0.10) | 0.14 (0.12 to 0.17) | 2.16 (0.25 to 4.10) |
| Djibouti | 1 (0 to 1) | 1 (0 to 2) | 0.19 (0.09 to 0.33) | 0.09 (0.04 to 0.21) | -2.67 (-2.87 to -2.48) |
| Dominica | 0 (0 to 1) | 0 (0 to 1) | 0.58 (0.41 to 0.88) | 0.52 (0.35 to 0.73) | -0.29 (-0.41 to -0.16) |
| Dominican Republic | 5 (4 to 9) | 8 (5 to 11) | 0.07 (0.05 to 0.13) | 0.08 (0.04 to 0.11) | 0.47 (0.31 to 0.64) |
| Ecuador | 14 (11 to 17) | 11 (8 to 16) | 0.23 (0.18 to 0.28) | 0.07 (0.05 to 0.10) | -4.99 (-5.61 to -4.36) |
| Egypt | 10 (1 to 39) | 5 (1 to 17) | 0.02 (0.00 to 0.07) | 0.01 (0.00 to 0.03) | -3.30 (-3.46 to -3.15) |
| El Salvador | 3 (2 to 5) | 3 (2 to 4) | 0.06 (0.04 to 0.10) | 0.04 (0.02 to 0.06) | -1.72 (-2.04 to -1.41) |
| Equatorial Guinea | 1 (1 to 2) | 1 (1 to 2) | 0.47 (0.21 to 0.86) | 0.14 (0.08 to 0.28) | -4.56 (-4.94 to -4.19) |
| Eritrea | 8 (4 to 16) | 7 (4 to 13) | 0.26 (0.12 to 0.49) | 0.14 (0.06 to 0.29) | -2.18 (-2.37 to -2.00) |
| Estonia | 3 (2 to 3) | 2 (2 to 3) | 0.15 (0.12 to 0.19) | 0.08 (0.06 to 0.10) | -2.56 (-3.21 to -1.90) |
| Eswatini | 2 (1 to 2) | 1 (1 to 2) | 0.31 (0.19 to 0.49) | 0.16 (0.09 to 0.26) | -2.19 (-2.29 to -2.10) |
| Ethiopia | 107 (46 to 229) | 71 (38 to 142) | 0.22 (0.09 to 0.43) | 0.09 (0.04 to 0.20) | -3.31 (-3.45 to -3.17) |
| Fiji | 1 (1 to 2) | 2 (1 to 2) | 0.18 (0.14 to 0.26) | 0.19 (0.12 to 0.29) | 0.78 (0.49 to 1.08) |
| Finland | 19 (16 to 21) | 39 (31 to 44) | 0.31 (0.28 to 0.36) | 0.32 (0.27 to 0.36) | 0.56 (-1.05 to 2.19) |
| France | 13 (12 to 15) | 107 (87 to 130) | 0.02 (0.02 to 0.02) | 0.06 (0.05 to 0.07) | 4.62 (2.48 to 6.80) |
| Gabon | 2 (1 to 4) | 2 (1 to 4) | 0.36 (0.18 to 0.66) | 0.16 (0.09 to 0.30) | -2.69 (-2.77 to -2.61) |
| Gambia | 2 (1 to 4) | 3 (1 to 6) | 0.31 (0.15 to 0.56) | 0.15 (0.08 to 0.33) | -2.86 (-3.15 to -2.56) |
| Georgia | 48 (36 to 63) | 36 (26 to 49) | 0.85 (0.62 to 1.11) | 0.65 (0.46 to 0.88) | -1.35 (-2.09 to -0.61) |
| Germany | 159 (110 to 203) | 297 (243 to 348) | 0.15 (0.10 to 0.18) | 0.18 (0.15 to 0.20) | 0.67 (-0.47 to 1.82) |
| Ghana | 49 (32 to 72) | 50 (28 to 90) | 0.45 (0.23 to 0.74) | 0.20 (0.10 to 0.36) | -3.04 (-3.29 to -2.80) |
| Greece | 5 (4 to 6) | 19 (15 to 23) | 0.04 (0.03 to 0.05) | 0.07 (0.06 to 0.09) | 2.95 (0.61 to 5.34) |
| Greenland | 0 (0 to 0) | 0 (0 to 0) | 0.21 (0.13 to 0.31) | 0.16 (0.06 to 0.24) | -0.66 (-0.97 to -0.35) |
| Grenada | 0 (0 to 0) | 0 (0 to 1) | 0.37 (0.31 to 0.48) | 0.49 (0.42 to 0.55) | 0.51 (0.12 to 0.90) |
| Guam | 0 (0 to 0) | 0 (0 to 0) | 0.32 (0.21 to 0.45) | 0.12 (0.09 to 0.19) | -2.01 (-2.38 to -1.63) |
| Guatemala | 11 (8 to 13) | 22 (18 to 27) | 0.18 (0.14 to 0.25) | 0.18 (0.15 to 0.21) | 0.22 (-0.18 to 0.63) |
| Guinea | 21 (11 to 37) | 19 (10 to 35) | 0.37 (0.17 to 0.69) | 0.17 (0.09 to 0.36) | -2.65 (-2.81 to -2.49) |
| Guinea-Bissau | 3 (2 to 6) | 3 (1 to 6) | 0.42 (0.19 to 0.76) | 0.18 (0.08 to 0.38) | -2.98 (-3.14 to -2.82) |
| Guyana | 6 (5 to 7) | 14 (11 to 18) | 0.89 (0.76 to 1.02) | 2.17 (1.63 to 2.74) | 3.66 (3.37 to 3.95) |
| Haiti | 42 (19 to 92) | 71 (36 to 119) | 0.75 (0.33 to 1.39) | 0.66 (0.32 to 1.10) | -0.30 (-0.37 to -0.23) |
| Honduras | 5 (3 to 8) | 8 (4 to 14) | 0.17 (0.08 to 0.31) | 0.15 (0.08 to 0.24) | -0.35 (-0.45 to -0.24) |
| Hungary | 39 (29 to 50) | 46 (33 to 61) | 0.33 (0.25 to 0.41) | 0.25 (0.18 to 0.34) | -0.94 (-1.46 to -0.41) |
| Iceland | 0 (0 to 0) | 1 (1 to 1) | 0.05 (0.05 to 0.06) | 0.16 (0.13 to 0.18) | 3.87 (1.60 to 6.19) |
| India | 1987 (1051 to 2995) | 2957 (1907 to 4251) | 0.32 (0.16 to 0.48) | 0.26 (0.17 to 0.37) | -0.37 (-0.53 to -0.20) |
| Indonesia | 303 (175 to 585) | 524 (347 to 873) | 0.32 (0.19 to 0.55) | 0.37 (0.27 to 0.54) | 0.40 (0.21 to 0.59) |
| Iran (Islamic Republic of) | 109 (62 to 148) | 165 (86 to 214) | 0.45 (0.26 to 0.61) | 0.24 (0.13 to 0.31) | -1.23 (-1.89 to -0.57) |
| Iraq | 186 (128 to 253) | 222 (160 to 314) | 1.50 (0.97 to 2.42) | 0.96 (0.68 to 1.52) | -1.70 (-1.83 to -1.57) |
| Ireland | 3 (3 to 4) | 30 (24 to 36) | 0.10 (0.09 to 0.11) | 0.39 (0.31 to 0.45) | 5.20 (3.12 to 7.33) |
| Israel | 2 (2 to 3) | 10 (8 to 11) | 0.05 (0.04 to 0.06) | 0.07 (0.06 to 0.08) | 1.74 (-0.55 to 4.07) |
| Italy | 2473 (1934 to 2889) | 738 (549 to 862) | 3.44 (2.66 to 4.04) | 0.34 (0.26 to 0.39) | -8.36 (-10.10 to -6.59) |
| Jamaica | 2 (2 to 3) | 5 (4 to 6) | 0.11 (0.09 to 0.14) | 0.15 (0.12 to 0.20) | 1.47 (1.10 to 1.85) |
| Japan | 212 (199 to 223) | 427 (354 to 485) | 0.16 (0.15 to 0.17) | 0.17 (0.15 to 0.18) | -0.83 (-1.55 to -0.11) |
| Jordan | 3 (2 to 4) | 5 (4 to 7) | 0.15 (0.10 to 0.24) | 0.07 (0.05 to 0.10) | -2.78 (-3.02 to -2.53) |
| Kazakhstan | 14 (9 to 20) | 445 (334 to 600) | 0.10 (0.07 to 0.16) | 2.48 (1.83 to 3.31) | 13.64 (11.37 to 15.95) |
| Kenya | 19 (12 to 28) | 24 (11 to 54) | 0.10 (0.06 to 0.18) | 0.07 (0.03 to 0.16) | -1.27 (-1.32 to -1.22) |
| Kiribati | 0 (0 to 1) | 1 (0 to 1) | 0.67 (0.35 to 1.01) | 0.65 (0.36 to 1.02) | -0.16 (-0.22 to -0.11) |
| Kuwait | 3 (2 to 3) | 6 (5 to 8) | 0.30 (0.26 to 0.34) | 0.25 (0.19 to 0.31) | -1.02 (-1.85 to -0.18) |
| Kyrgyzstan | 12 (9 to 15) | 25 (20 to 32) | 0.33 (0.26 to 0.44) | 0.54 (0.41 to 0.71) | 2.61 (1.51 to 3.73) |
| Lao People's Democratic Republic | 11 (4 to 27) | 16 (9 to 27) | 0.56 (0.22 to 1.05) | 0.40 (0.23 to 0.64) | -1.15 (-1.35 to -0.95) |
| Latvia | 3 (2 to 3) | 3 (3 to 4) | 0.09 (0.07 to 0.11) | 0.09 (0.07 to 0.12) | -0.36 (-0.83 to 0.12) |
| Lebanon | 12 (7 to 22) | 21 (14 to 34) | 0.60 (0.32 to 1.11) | 0.33 (0.22 to 0.52) | -2.25 (-2.42 to -2.07) |
| Lesotho | 3 (2 to 5) | 2 (1 to 4) | 0.30 (0.17 to 0.53) | 0.18 (0.10 to 0.30) | -1.71 (-1.82 to -1.60) |
| Liberia | 9 (5 to 19) | 6 (3 to 13) | 0.40 (0.18 to 0.79) | 0.15 (0.08 to 0.36) | -3.50 (-3.77 to -3.24) |
| Libya | 13 (3 to 41) | 12 (2 to 42) | 0.40 (0.08 to 1.27) | 0.27 (0.05 to 0.91) | -1.17 (-1.32 to -1.02) |
| Lithuania | 3 (2 to 4) | 4 (3 to 4) | 0.08 (0.06 to 0.10) | 0.06 (0.05 to 0.08) | -0.88 (-1.53 to -0.23) |
| Luxembourg | 0 (0 to 0) | 2 (2 to 2) | 0.07 (0.06 to 0.07) | 0.16 (0.13 to 0.18) | 3.18 (1.97 to 4.41) |
| Madagascar | 41 (20 to 70) | 45 (25 to 91) | 0.34 (0.17 to 0.53) | 0.19 (0.10 to 0.46) | -1.83 (-1.87 to -1.78) |
| Malawi | 20 (8 to 37) | 14 (8 to 28) | 0.18 (0.08 to 0.28) | 0.10 (0.05 to 0.22) | -2.05 (-2.18 to -1.92) |
| Malaysia | 37 (23 to 52) | 48 (33 to 64) | 0.33 (0.19 to 0.49) | 0.22 (0.15 to 0.32) | -1.59 (-1.81 to -1.38) |
| Maldives | 1 (0 to 1) | 1 (1 to 1) | 0.49 (0.27 to 0.76) | 0.24 (0.16 to 0.35) | -2.63 (-2.84 to -2.42) |
| Mali | 22 (12 to 41) | 25 (13 to 48) | 0.31 (0.13 to 0.57) | 0.13 (0.06 to 0.26) | -3.10 (-3.34 to -2.86) |
| Malta | 1 (1 to 1) | 6 (5 to 7) | 0.28 (0.25 to 0.31) | 0.62 (0.52 to 0.71) | 2.11 (-0.14 to 4.41) |
| Marshall Islands | 0 (0 to 0) | 0 (0 to 0) | 0.23 (0.09 to 0.47) | 0.17 (0.08 to 0.30) | -1.11 (-1.17 to -1.06) |
| Mauritania | 5 (3 to 8) | 4 (2 to 8) | 0.30 (0.15 to 0.57) | 0.12 (0.06 to 0.27) | -3.45 (-3.63 to -3.27) |
| Mauritius | 0 (0 to 0) | 2 (2 to 2) | 0.02 (0.02 to 0.02) | 0.12 (0.11 to 0.14) | 4.57 (2.66 to 6.51) |
| Mexico | 22 (21 to 24) | 136 (120 to 155) | 0.04 (0.04 to 0.04) | 0.12 (0.11 to 0.14) | 5.34 (4.75 to 5.94) |
| Micronesia (Federated States of) | 0 (0 to 0) | 0 (0 to 0) | 0.28 (0.10 to 0.67) | 0.17 (0.07 to 0.32) | -1.80 (-1.93 to -1.67) |
| Monaco | 0 (0 to 0) | 0 (0 to 0) | 0.25 (0.13 to 0.40) | 0.11 (0.06 to 0.17) | -2.88 (-3.19 to -2.57) |
| Mongolia | 26 (13 to 47) | 21 (13 to 32) | 2.25 (1.03 to 4.31) | 0.86 (0.53 to 1.29) | -3.79 (-4.21 to -3.37) |
| Montenegro | 5 (3 to 8) | 5 (3 to 7) | 0.85 (0.52 to 1.33) | 0.59 (0.35 to 0.92) | -1.51 (-1.79 to -1.23) |
| Morocco | 97 (16 to 308) | 137 (25 to 474) | 0.61 (0.09 to 2.08) | 0.46 (0.08 to 1.60) | -0.91 (-0.98 to -0.84) |
| Mozambique | 20 (9 to 38) | 20 (10 to 36) | 0.15 (0.07 to 0.25) | 0.09 (0.04 to 0.21) | -1.40 (-1.48 to -1.32) |
| Myanmar | 112 (53 to 228) | 158 (101 to 260) | 0.55 (0.26 to 0.94) | 0.41 (0.26 to 0.64) | -1.20 (-1.41 to -0.98) |
| Namibia | 2 (2 to 4) | 3 (2 to 5) | 0.28 (0.18 to 0.44) | 0.16 (0.10 to 0.27) | -1.97 (-2.07 to -1.86) |
| Nauru | 0 (0 to 0) | 0 (0 to 0) | 0.17 (0.07 to 0.30) | 0.31 (0.10 to 0.91) | 1.35 (1.03 to 1.68) |
| Nepal | 56 (29 to 90) | 75 (44 to 128) | 0.44 (0.20 to 0.84) | 0.34 (0.19 to 0.58) | -0.83 (-0.89 to -0.76) |
| Netherlands | 10 (9 to 11) | 45 (37 to 52) | 0.06 (0.05 to 0.06) | 0.13 (0.11 to 0.15) | 2.60 (0.46 to 4.78) |
| New Zealand | 13 (12 to 15) | 18 (17 to 20) | 0.39 (0.36 to 0.43) | 0.30 (0.28 to 0.33) | -0.71 (-1.56 to 0.15) |
| Nicaragua | 3 (2 to 4) | 3 (2 to 4) | 0.08 (0.06 to 0.11) | 0.05 (0.04 to 0.07) | -0.86 (-1.02 to -0.70) |
| Niger | 24 (12 to 47) | 30 (16 to 55) | 0.40 (0.16 to 0.79) | 0.17 (0.07 to 0.39) | -3.05 (-3.30 to -2.80) |
| Nigeria | 279 (157 to 518) | 241 (157 to 414) | 0.39 (0.17 to 0.73) | 0.12 (0.07 to 0.23) | -4.41 (-4.78 to -4.05) |
| Niue | 0 (0 to 0) | 0 (0 to 0) | 0.18 (0.07 to 0.33) | 0.23 (0.11 to 0.35) | -0.94 (-1.57 to -0.31) |
| North Macedonia | 8 (5 to 13) | 10 (5 to 18) | 0.47 (0.29 to 0.79) | 0.42 (0.22 to 0.71) | -1.38 (-1.80 to -0.95) |
| Northern Mariana Islands | 0 (0 to 0) | 0 (0 to 0) | 0.11 (0.07 to 0.16) | 0.15 (0.07 to 0.24) | 0.78 (0.37 to 1.19) |
| Norway | 7 (7 to 8) | 10 (9 to 12) | 0.14 (0.13 to 0.15) | 0.11 (0.10 to 0.13) | 0.27 (-0.93 to 1.48) |
| Oman | 7 (4 to 12) | 11 (6 to 17) | 0.85 (0.50 to 1.41) | 0.50 (0.30 to 0.77) | -1.53 (-1.81 to -1.25) |
| Pakistan | 340 (197 to 545) | 626 (383 to 994) | 0.39 (0.20 to 0.62) | 0.40 (0.23 to 0.68) | 0.14 (-0.09 to 0.37) |
| Palau | 0 (0 to 0) | 0 (0 to 0) | 0.13 (0.05 to 0.21) | 0.08 (0.04 to 0.12) | -1.40 (-1.50 to -1.31) |
| Palestine | 3 (2 to 5) | 5 (2 to 7) | 0.30 (0.18 to 0.49) | 0.22 (0.10 to 0.34) | -1.34 (-1.55 to -1.12) |
| Panama | 1 (1 to 2) | 4 (3 to 5) | 0.07 (0.06 to 0.09) | 0.09 (0.08 to 0.11) | 0.68 (0.24 to 1.12) |
| Papua New Guinea | 10 (4 to 19) | 26 (13 to 46) | 0.22 (0.09 to 0.40) | 0.22 (0.10 to 0.41) | 0.12 (0.01 to 0.22) |
| Paraguay | 4 (2 to 5) | 4 (3 to 6) | 0.11 (0.07 to 0.16) | 0.07 (0.05 to 0.10) | -1.56 (-1.65 to -1.46) |
| Peru | 31 (16 to 48) | 15 (10 to 25) | 0.22 (0.10 to 0.34) | 0.04 (0.03 to 0.07) | -5.54 (-5.76 to -5.33) |
| Philippines | 65 (41 to 82) | 102 (57 to 130) | 0.21 (0.10 to 0.30) | 0.15 (0.07 to 0.19) | -0.70 (-1.06 to -0.34) |
| Poland | 162 (150 to 175) | 202 (168 to 242) | 0.44 (0.41 to 0.48) | 0.31 (0.25 to 0.37) | -1.63 (-2.33 to -0.94) |
| Portugal | 3 (3 to 3) | 20 (16 to 24) | 0.03 (0.03 to 0.03) | 0.08 (0.07 to 0.09) | 3.14 (0.84 to 5.49) |
| Puerto Rico | 1 (1 to 1) | 4 (3 to 4) | 0.04 (0.03 to 0.04) | 0.06 (0.05 to 0.08) | 1.17 (-0.25 to 2.61) |
| Qatar | 1 (0 to 1) | 2 (1 to 4) | 0.51 (0.30 to 0.78) | 0.24 (0.12 to 0.42) | -3.10 (-3.60 to -2.60) |
| Republic of Korea | 123 (81 to 174) | 78 (54 to 111) | 0.41 (0.26 to 0.64) | 0.12 (0.09 to 0.17) | -4.01 (-4.44 to -3.59) |
| Republic of Moldova | 6 (5 to 7) | 6 (5 to 8) | 0.17 (0.14 to 0.20) | 0.12 (0.09 to 0.14) | -1.55 (-2.09 to -1.01) |
| Romania | 703 (504 to 975) | 1782 (1382 to 2285) | 3.44 (2.55 to 4.65) | 5.12 (3.90 to 6.59) | 0.48 (-0.08 to 1.03) |
| Russian Federation | 681 (624 to 724) | 964 (869 to 1052) | 0.57 (0.51 to 0.60) | 0.46 (0.41 to 0.51) | -0.87 (-1.60 to -0.13) |
| Rwanda | 20 (9 to 41) | 10 (6 to 20) | 0.28 (0.13 to 0.51) | 0.10 (0.05 to 0.21) | -4.07 (-4.37 to -3.76) |
| Saint Kitts and Nevis | 0 (0 to 0) | 0 (0 to 0) | 0.07 (0.06 to 0.10) | 0.14 (0.11 to 0.17) | 2.37 (1.93 to 2.81) |
| Saint Lucia | 1 (1 to 1) | 1 (1 to 2) | 0.81 (0.69 to 0.95) | 0.64 (0.52 to 0.77) | -1.15 (-1.34 to -0.96) |
| Saint Vincent and the Grenadines | 0 (0 to 0) | 0 (0 to 1) | 0.16 (0.14 to 0.18) | 0.36 (0.31 to 0.43) | 1.78 (0.79 to 2.77) |
| Samoa | 0 (0 to 0) | 0 (0 to 0) | 0.19 (0.08 to 0.37) | 0.12 (0.06 to 0.18) | -1.45 (-1.54 to -1.36) |
| San Marino | 0 (0 to 0) | 0 (0 to 0) | 0.27 (0.16 to 0.49) | 0.21 (0.11 to 0.33) | 0.91 (0.38 to 1.43) |
| Sao Tome and Principe | 0 (0 to 0) | 0 (0 to 1) | 0.33 (0.15 to 0.66) | 0.12 (0.05 to 0.30) | -3.45 (-3.74 to -3.16) |
| Saudi Arabia | 74 (48 to 107) | 66 (44 to 101) | 1.00 (0.62 to 1.58) | 0.37 (0.24 to 0.59) | -3.69 (-3.94 to -3.44) |
| Senegal | 22 (14 to 37) | 20 (9 to 42) | 0.34 (0.18 to 0.60) | 0.16 (0.08 to 0.35) | -2.72 (-2.87 to -2.57) |
| Serbia | 47 (26 to 72) | 36 (20 to 54) | 0.61 (0.34 to 0.96) | 0.23 (0.14 to 0.35) | -3.86 (-4.23 to -3.49) |
| Seychelles | 0 (0 to 1) | 0 (0 to 1) | 0.77 (0.52 to 1.14) | 0.52 (0.37 to 0.68) | -0.99 (-1.13 to -0.84) |
| Sierra Leone | 16 (8 to 33) | 14 (7 to 28) | 0.39 (0.19 to 0.68) | 0.17 (0.08 to 0.36) | -3.11 (-3.33 to -2.90) |
| Singapore | 51 (48 to 54) | 49 (42 to 56) | 1.75 (1.64 to 1.85) | 0.67 (0.58 to 0.75) | -3.54 (-3.85 to -3.22) |
| Slovakia | 13 (9 to 20) | 19 (12 to 26) | 0.25 (0.18 to 0.38) | 0.22 (0.14 to 0.31) | -0.41 (-0.55 to -0.27) |
| Slovenia | 3 (3 to 4) | 8 (5 to 11) | 0.15 (0.12 to 0.19) | 0.15 (0.10 to 0.20) | -1.13 (-1.67 to -0.58) |
| Solomon Islands | 0 (0 to 1) | 1 (0 to 1) | 0.15 (0.05 to 0.32) | 0.13 (0.05 to 0.27) | -0.31 (-0.36 to -0.26) |
| Somalia | 25 (10 to 49) | 31 (13 to 63) | 0.30 (0.13 to 0.57) | 0.18 (0.07 to 0.44) | -1.61 (-1.76 to -1.47) |
| South Africa | 70 (39 to 95) | 59 (44 to 98) | 0.25 (0.14 to 0.35) | 0.12 (0.09 to 0.20) | -3.00 (-3.29 to -2.71) |
| South Sudan | 16 (5 to 30) | 15 (6 to 28) | 0.24 (0.10 to 0.41) | 0.15 (0.06 to 0.26) | -1.74 (-2.25 to -1.24) |
| Spain | 20 (17 to 23) | 237 (180 to 292) | 0.05 (0.04 to 0.05) | 0.18 (0.14 to 0.21) | 4.78 (3.06 to 6.54) |
| Sri Lanka | 295 (175 to 389) | 142 (88 to 241) | 3.10 (1.96 to 4.18) | 0.67 (0.42 to 1.14) | -6.73 (-7.63 to -5.82) |
| Sudan | 82 (12 to 270) | 89 (14 to 301) | 0.65 (0.09 to 2.23) | 0.41 (0.06 to 1.47) | -1.53 (-1.58 to -1.48) |
| Suriname | 1 (0 to 1) | 1 (0 to 1) | 0.16 (0.11 to 0.22) | 0.14 (0.09 to 0.19) | 0.04 (-0.12 to 0.20) |
| Sweden | 85 (76 to 94) | 67 (56 to 79) | 0.72 (0.66 to 0.78) | 0.39 (0.32 to 0.46) | -2.04 (-2.67 to -1.41) |
| Switzerland | 4 (3 to 4) | 13 (10 to 16) | 0.04 (0.04 to 0.05) | 0.06 (0.05 to 0.07) | 1.53 (-0.33 to 3.43) |
| Syrian Arab Republic | 41 (8 to 122) | 42 (7 to 139) | 0.56 (0.09 to 1.83) | 0.38 (0.06 to 1.28) | -1.56 (-1.72 to -1.40) |
| Taiwan (Province of China) | 27 (24 to 29) | 172 (146 to 194) | 0.22 (0.20 to 0.24) | 0.44 (0.38 to 0.49) | 4.99 (3.92 to 6.08) |
| Tajikistan | 0 (0 to 0) | 0 (0 to 1) | 0.01 (0.00 to 0.02) | 0.01 (0.00 to 0.01) | -1.56 (-2.06 to -1.05) |
| Thailand | 79 (51 to 116) | 175 (118 to 245) | 0.24 (0.14 to 0.36) | 0.19 (0.13 to 0.26) | -1.17 (-1.53 to -0.79) |
| Timor-Leste | 2 (1 to 4) | 3 (2 to 7) | 0.47 (0.21 to 0.92) | 0.46 (0.25 to 0.88) | -0.14 (-0.24 to -0.03) |
| Togo | 9 (5 to 14) | 9 (5 to 17) | 0.33 (0.17 to 0.58) | 0.15 (0.08 to 0.32) | -2.93 (-3.12 to -2.73) |
| Tokelau | 0 (0 to 0) | 0 (0 to 0) | 0.20 (0.08 to 0.38) | 0.33 (0.14 to 0.58) | -0.75 (-1.64 to 0.15) |
| Tonga | 0 (0 to 0) | 0 (0 to 0) | 0.11 (0.05 to 0.21) | 0.08 (0.03 to 0.14) | -1.10 (-1.17 to -1.03) |
| Trinidad and Tobago | 5 (5 to 6) | 9 (7 to 11) | 0.59 (0.53 to 0.66) | 0.53 (0.41 to 0.68) | -0.34 (-0.45 to -0.23) |
| Tunisia | 24 (5 to 75) | 42 (7 to 165) | 0.47 (0.09 to 1.58) | 0.37 (0.06 to 1.48) | -0.84 (-0.94 to -0.74) |
| Turkmenistan | 20 (16 to 26) | 48 (37 to 62) | 0.85 (0.68 to 1.10) | 1.07 (0.83 to 1.38) | 0.87 (-0.48 to 2.24) |
| Tuvalu | 0 (0 to 0) | 0 (0 to 0) | 0.24 (0.11 to 0.47) | 0.15 (0.07 to 0.25) | -1.68 (-1.75 to -1.60) |
| Türkiye | 44 (18 to 82) | 69 (42 to 98) | 0.11 (0.04 to 0.23) | 0.08 (0.05 to 0.12) | -0.82 (-1.28 to -0.37) |
| Uganda | 42 (18 to 81) | 33 (19 to 53) | 0.21 (0.10 to 0.34) | 0.09 (0.05 to 0.17) | -2.96 (-3.13 to -2.80) |
| Ukraine | 194 (147 to 242) | 394 (282 to 534) | 0.38 (0.29 to 0.47) | 0.58 (0.41 to 0.77) | 1.39 (1.20 to 1.58) |
| United Arab Emirates | 5 (3 to 8) | 12 (8 to 18) | 0.82 (0.49 to 1.42) | 0.48 (0.32 to 0.67) | 1.04 (-0.12 to 2.22) |
| United Kingdom | 339 (316 to 359) | 331 (287 to 366) | 0.48 (0.45 to 0.50) | 0.31 (0.27 to 0.34) | -1.91 (-2.34 to -1.48) |
| United Republic of Tanzania | 53 (27 to 91) | 46 (26 to 93) | 0.21 (0.11 to 0.33) | 0.10 (0.05 to 0.22) | -2.67 (-2.78 to -2.55) |
| United States Virgin Islands | 1 (0 to 1) | 1 (0 to 1) | 0.74 (0.52 to 1.14) | 0.67 (0.40 to 1.01) | 0.32 (-0.02 to 0.66) |
| United States of America | 746 (694 to 795) | 755 (710 to 799) | 0.29 (0.27 to 0.31) | 0.21 (0.20 to 0.22) | -1.11 (-1.52 to -0.71) |
| Uruguay | 9 (8 to 11) | 9 (7 to 10) | 0.28 (0.23 to 0.32) | 0.16 (0.14 to 0.18) | -2.24 (-2.74 to -1.75) |
| Uzbekistan | 47 (36 to 65) | 50 (38 to 64) | 0.33 (0.23 to 0.48) | 0.18 (0.14 to 0.24) | -1.78 (-2.47 to -1.10) |
| Vanuatu | 0 (0 to 0) | 0 (0 to 1) | 0.17 (0.08 to 0.31) | 0.16 (0.08 to 0.30) | -0.30 (-0.43 to -0.16) |
| Venezuela (Bolivarian Republic of) | 15 (13 to 18) | 24 (18 to 31) | 0.10 (0.08 to 0.11) | 0.09 (0.07 to 0.12) | -0.71 (-1.07 to -0.34) |
| Viet Nam | 155 (84 to 286) | 237 (138 to 375) | 0.42 (0.21 to 0.82) | 0.32 (0.19 to 0.50) | -1.07 (-1.28 to -0.86) |
| Yemen | 47 (7 to 163) | 82 (14 to 280) | 0.71 (0.10 to 2.52) | 0.56 (0.08 to 1.98) | -0.89 (-0.98 to -0.81) |
| Zambia | 15 (6 to 34) | 25 (8 to 74) | 0.19 (0.09 to 0.37) | 0.20 (0.05 to 0.61) | 0.58 (0.05 to 1.11) |
| Zimbabwe | 17 (11 to 31) | 34 (20 to 60) | 0.30 (0.18 to 0.62) | 0.32 (0.18 to 0.61) | 0.58 (0.44 to 0.71) |

# Table S7. The DALY and age-standardized DALY rate of myocarditis in 1990 and 2021, and EAPC in age-standardized DALY rate from 1990 to 2021

| **Characteristics** | **DALY (95% UI)** | | **ASYR (95% UI)** | | **EAPC in ASYR (95% CI)** |
| --- | --- | --- | --- | --- | --- |
|  | **1990** | **2021** | **1990** | **2021** | **1990-2021** |
| **Global** | 1032803 (829855 to 1303917) | 963139 (795811 to 1148366) | 19.93 (16.23 to 24.41) | 12.41 (10.37 to 14.76) | -1.71 (-1.95 to -1.46) |
| **Gender** |  |  |  |  |  |
| Male | 575396 (424111 to 781672) | 568274 (442834 to 724676) | 22.23 (17.01 to 29.44) | 15.01 (11.70 to 18.97) | -1.52 (-1.74 to -1.31) |
| Female | 457407 (332414 to 604603) | 394865 (319314 to 477649) | 17.56 (13.07 to 22.60) | 9.89 (8.06 to 12.05) | -1.92 (-2.21 to -1.63) |
| **SDI** |  |  |  |  |  |
| High-middle SDI | 238223 (199699 to 292428) | 236182 (194410 to 269141) | 26.93 (22.63 to 32.25) | 15.86 (13.08 to 18.04) | -2.22 (-2.66 to -1.79) |
| High SDI | 105119 (99690 to 110602) | 101009 (95171 to 107271) | 12.81 (12.14 to 13.50) | 9.14 (8.61 to 9.72) | -1.20 (-1.69 to -0.71) |
| Low-middle SDI | 158859 (96985 to 268548) | 176100 (127094 to 258630) | 13.70 (8.03 to 21.83) | 10.14 (7.28 to 15.03) | -0.85 (-0.93 to -0.76) |
| Low SDI | 92510 (51522 to 156341) | 103706 (69430 to 171338) | 15.54 (8.37 to 25.35) | 9.71 (6.16 to 17.06) | -1.50 (-1.60 to -1.40) |
| Middle SDI | 437112 (332991 to 553393) | 344782 (247276 to 418600) | 26.71 (20.75 to 33.10) | 15.14 (10.90 to 18.35) | -1.98 (-2.21 to -1.76) |
| **Region** |  |  |  |  |  |
| East Asia | 473750 (358425 to 616499) | 353533 (246626 to 445035) | 44.00 (33.35 to 56.75) | 25.02 (17.86 to 30.91) | -2.15 (-2.48 to -1.82) |
| Southeast Asia | 50464 (35570 to 85960) | 50418 (38975 to 77223) | 12.68 (9.08 to 20.35) | 8.27 (6.54 to 12.34) | -1.75 (-1.95 to -1.55) |
| Oceania | 1087 (576 to 1848) | 2288 (1220 to 3933) | 13.13 (6.98 to 21.88) | 13.63 (7.38 to 22.92) | 0.18 (0.10 to 0.27) |
| Central Asia | 9232 (7906 to 10939) | 25903 (20562 to 32938) | 15.08 (12.82 to 17.98) | 27.41 (21.93 to 34.56) | 2.61 (1.78 to 3.44) |
| Central Europe | 35566 (30539 to 41847) | 58674 (45631 to 72978) | 29.42 (25.77 to 33.78) | 34.11 (26.56 to 42.43) | -0.30 (-0.74 to 0.15) |
| Eastern Europe | 22332 (19933 to 24489) | 34274 (29582 to 38861) | 10.62 (9.56 to 11.56) | 13.25 (11.44 to 15.01) | 0.28 (-0.24 to 0.80) |
| High-income Asia Pacific | 18386 (16213 to 20865) | 15808 (14267 to 17262) | 12.09 (10.63 to 13.86) | 8.56 (7.71 to 9.33) | -1.61 (-2.03 to -1.18) |
| Australasia | 4700 (4261 to 5119) | 2835 (2570 to 3092) | 24.12 (22.12 to 26.08) | 9.18 (8.32 to 10.03) | -3.42 (-4.08 to -2.77) |
| Western Europe | 56141 (49230 to 61650) | 37228 (32299 to 41396) | 12.43 (11.17 to 13.49) | 6.01 (5.33 to 6.65) | -2.79 (-3.85 to -1.71) |
| Southern Latin America | 5428 (4866 to 6166) | 2502 (2288 to 2742) | 11.08 (9.90 to 12.59) | 3.67 (3.32 to 4.07) | -3.60 (-4.03 to -3.18) |
| High-income North America | 44299 (42220 to 46645) | 39523 (37361 to 41810) | 16.74 (16.06 to 17.53) | 12.17 (11.42 to 12.99) | -1.03 (-1.47 to -0.59) |
| Caribbean | 5115 (3321 to 9837) | 7032 (4745 to 10398) | 13.66 (8.99 to 24.93) | 15.92 (10.55 to 23.97) | 0.72 (0.64 to 0.79) |
| Andean Latin America | 2545 (1585 to 3998) | 1326 (972 to 1697) | 7.21 (4.63 to 10.63) | 2.12 (1.56 to 2.71) | -4.45 (-4.73 to -4.17) |
| Central Latin America | 5964 (5516 to 6589) | 11467 (9827 to 13482) | 3.27 (3.03 to 3.57) | 4.92 (4.18 to 5.85) | 1.67 (1.42 to 1.91) |
| Tropical Latin America | 12777 (11412 to 14419) | 14260 (13177 to 15371) | 8.79 (7.99 to 9.77) | 6.51 (5.93 to 7.13) | -1.16 (-1.86 to -0.47) |
| North Africa and Middle East | 45314 (22102 to 98996) | 42285 (23831 to 89419) | 14.23 (6.75 to 29.97) | 8.20 (4.63 to 17.55) | -1.75 (-1.86 to -1.65) |
| South Asia | 153725 (89113 to 245048) | 182543 (125991 to 255512) | 14.36 (7.91 to 21.58) | 11.00 (7.65 to 15.20) | -0.59 (-0.70 to -0.49) |
| Central Sub-Saharan Africa | 10802 (5474 to 18852) | 12117 (6799 to 23106) | 17.27 (8.98 to 26.64) | 10.18 (5.28 to 22.40) | -1.72 (-1.81 to -1.63) |
| Eastern Sub-Saharan Africa | 30613 (12858 to 55819) | 23165 (14210 to 38839) | 11.64 (6.04 to 19.45) | 5.36 (3.17 to 10.02) | -2.64 (-2.76 to -2.52) |
| Southern Sub-Saharan Africa | 5350 (3472 to 6754) | 5250 (3865 to 8491) | 10.52 (6.84 to 13.57) | 6.86 (5.06 to 11.00) | -1.63 (-1.76 to -1.49) |
| Western Sub-Saharan Africa | 39213 (25765 to 76420) | 40708 (26678 to 70163) | 15.78 (9.67 to 26.55) | 6.89 (4.58 to 12.02) | -3.04 (-3.30 to -2.78) |

# Table S8. The DALY and age-standardized DALY rate of myocarditis in 1990 and 2021, and EAPC in age-standardized DALY rate from 1990 to 2021 in 204 countries and territories

| **Characteristics** | **DALY (95% UI)** | | **ASYR (95% UI)** | | **EAPC in ASYR**  **(95% CI)** |
| --- | --- | --- | --- | --- | --- |
|  | **1990** | **2021** | **1990** | **2021** | **1990-2021** |
| Afghanistan | 2334 (331 to 8028) | 3879 (628 to 12540) | 23.19 (3.01 to 79.27) | 18.56 (2.68 to 62.90) | -0.68 (-0.85 to -0.51) |
| Albania | 867 (617 to 1182) | 696 (412 to 1169) | 29.99 (20.68 to 43.43) | 23.45 (14.50 to 37.00) | -1.17 (-1.44 to -0.90) |
| Algeria | 3144 (627 to 9611) | 3166 (693 to 10396) | 15.08 (2.97 to 44.87) | 8.58 (1.84 to 28.90) | -1.59 (-1.66 to -1.52) |
| American Samoa | 1 (1 to 2) | 1 (1 to 1) | 2.14 (1.34 to 3.09) | 1.87 (1.22 to 2.70) | -0.30 (-0.46 to -0.14) |
| Andorra | 8 (5 to 12) | 8 (5 to 12) | 17.51 (11.98 to 25.80) | 7.22 (4.87 to 9.97) | -2.68 (-2.79 to -2.58) |
| Angola | 2082 (910 to 4750) | 2831 (1613 to 4965) | 17.86 (8.95 to 32.17) | 9.42 (5.05 to 19.06) | -2.19 (-2.31 to -2.07) |
| Antigua and Barbuda | 13 (11 to 14) | 16 (15 to 17) | 22.13 (19.46 to 24.96) | 17.59 (16.20 to 19.07) | -0.37 (-0.61 to -0.13) |
| Argentina | 4239 (3694 to 4921) | 1855 (1686 to 2046) | 12.99 (11.30 to 15.07) | 4.08 (3.67 to 4.56) | -3.82 (-4.21 to -3.42) |
| Armenia | 147 (108 to 197) | 93 (60 to 137) | 4.73 (3.57 to 6.46) | 2.53 (1.68 to 3.68) | -2.63 (-3.62 to -1.62) |
| Australia | 3936 (3535 to 4335) | 2041 (1818 to 2262) | 24.27 (22.03 to 26.51) | 7.86 (6.97 to 8.73) | -4.02 (-4.67 to -3.36) |
| Austria | 154 (140 to 172) | 636 (548 to 727) | 1.88 (1.70 to 2.09) | 5.38 (4.64 to 6.20) | 4.01 (1.83 to 6.23) |
| Azerbaijan | 2090 (1151 to 3441) | 3366 (1639 to 6712) | 32.45 (17.02 to 54.59) | 28.98 (14.75 to 56.09) | -1.38 (-1.81 to -0.95) |
| Bahamas | 19 (16 to 22) | 27 (21 to 35) | 7.52 (6.55 to 8.74) | 6.93 (5.49 to 8.94) | 0.00 (-0.30 to 0.30) |
| Bahrain | 18 (12 to 27) | 22 (16 to 35) | 5.54 (3.45 to 7.67) | 2.35 (1.68 to 3.40) | -3.11 (-3.29 to -2.93) |
| Bangladesh | 16416 (6914 to 28582) | 17838 (10493 to 30895) | 17.11 (7.49 to 28.44) | 11.81 (6.96 to 20.23) | -1.07 (-1.20 to -0.93) |
| Barbados | 21 (18 to 24) | 31 (24 to 39) | 8.20 (7.06 to 9.62) | 9.15 (7.23 to 11.69) | 0.65 (0.49 to 0.82) |
| Belarus | 1064 (601 to 1610) | 481 (377 to 609) | 9.90 (5.93 to 14.46) | 4.11 (3.28 to 5.14) | -3.47 (-4.07 to -2.86) |
| Belgium | 205 (183 to 230) | 654 (549 to 756) | 1.98 (1.79 to 2.19) | 3.76 (3.23 to 4.32) | 2.47 (0.39 to 4.59) |
| Belize | 24 (21 to 27) | 21 (17 to 25) | 11.23 (9.37 to 13.30) | 5.11 (4.31 to 6.09) | -2.11 (-2.73 to -1.49) |
| Benin | 868 (497 to 1634) | 1040 (566 to 1901) | 12.92 (7.60 to 21.14) | 6.20 (3.60 to 11.20) | -2.67 (-2.91 to -2.44) |
| Bermuda | 4 (3 to 4) | 4 (4 to 5) | 6.14 (5.24 to 7.22) | 5.64 (4.55 to 7.02) | -0.40 (-0.89 to 0.09) |
| Bhutan | 84 (44 to 142) | 82 (48 to 152) | 14.68 (7.48 to 24.13) | 11.93 (7.18 to 21.39) | -0.93 (-1.19 to -0.67) |
| Bolivia (Plurinational State of) | 493 (254 to 1063) | 380 (229 to 602) | 7.88 (4.29 to 14.98) | 3.68 (2.22 to 5.81) | -2.51 (-2.57 to -2.46) |
| Bosnia and Herzegovina | 1085 (583 to 1903) | 853 (406 to 1612) | 26.53 (14.07 to 46.41) | 18.99 (9.00 to 35.13) | -1.66 (-1.89 to -1.43) |
| Botswana | 146 (94 to 230) | 148 (83 to 312) | 12.13 (7.76 to 19.42) | 6.58 (3.76 to 13.35) | -2.10 (-2.19 to -2.02) |
| Brazil | 12548 (11205 to 14177) | 14074 (13009 to 15141) | 8.92 (8.10 to 9.93) | 6.63 (6.03 to 7.24) | -1.15 (-1.85 to -0.45) |
| Brunei Darussalam | 107 (74 to 150) | 105 (74 to 142) | 38.58 (27.32 to 53.32) | 23.75 (17.15 to 31.82) | -1.43 (-1.57 to -1.28) |
| Bulgaria | 1881 (1544 to 2293) | 3374 (2416 to 4533) | 21.74 (18.21 to 26.00) | 34.95 (25.14 to 46.51) | -0.02 (-0.82 to 0.79) |
| Burkina Faso | 2046 (1087 to 4151) | 2406 (1325 to 4547) | 16.74 (8.91 to 29.01) | 8.43 (4.80 to 15.71) | -2.35 (-2.60 to -2.11) |
| Burundi | 1248 (497 to 2503) | 835 (443 to 1491) | 15.68 (7.39 to 28.26) | 6.08 (3.15 to 12.28) | -3.01 (-3.13 to -2.89) |
| Cabo Verde | 26 (12 to 56) | 15 (6 to 31) | 6.08 (2.97 to 12.14) | 2.93 (1.16 to 6.00) | -2.56 (-2.68 to -2.44) |
| Cambodia | 1007 (428 to 2312) | 1174 (701 to 1970) | 10.51 (4.69 to 21.67) | 8.39 (4.99 to 13.56) | -0.86 (-0.95 to -0.77) |
| Cameroon | 1742 (1080 to 2873) | 2465 (1452 to 4467) | 13.68 (8.05 to 21.63) | 6.86 (4.13 to 12.54) | -2.44 (-2.64 to -2.25) |
| Canada | 1830 (1691 to 1973) | 2981 (2706 to 3279) | 7.60 (7.01 to 8.21) | 8.76 (7.91 to 9.74) | 0.79 (-0.05 to 1.63) |
| Central African Republic | 653 (305 to 1239) | 828 (386 to 1532) | 21.20 (10.25 to 34.23) | 15.58 (6.92 to 31.78) | -1.10 (-1.26 to -0.93) |
| Chad | 1189 (574 to 2570) | 1959 (843 to 4662) | 14.71 (8.05 to 27.68) | 8.25 (3.99 to 17.29) | -2.22 (-2.42 to -2.02) |
| Chile | 787 (726 to 857) | 432 (393 to 480) | 5.87 (5.44 to 6.35) | 2.31 (2.04 to 2.61) | -2.52 (-3.18 to -1.86) |
| China | 466663 (351850 to 609205) | 343546 (237870 to 435937) | 44.86 (33.92 to 58.12) | 25.26 (17.76 to 31.38) | -2.21 (-2.55 to -1.87) |
| Colombia | 1511 (1325 to 1725) | 2995 (2480 to 3610) | 4.21 (3.72 to 4.76) | 6.67 (5.45 to 8.19) | 1.56 (1.11 to 2.01) |
| Comoros | 79 (31 to 148) | 36 (20 to 66) | 12.51 (6.05 to 21.18) | 4.92 (2.66 to 9.20) | -3.39 (-3.65 to -3.12) |
| Congo | 369 (198 to 580) | 368 (216 to 743) | 15.08 (8.07 to 23.00) | 7.67 (4.43 to 16.03) | -2.44 (-2.58 to -2.30) |
| Cook Islands | 0 (0 to 0) | 0 (0 to 0) | 0.49 (0.31 to 0.73) | 0.31 (0.21 to 0.47) | -1.86 (-2.01 to -1.72) |
| Costa Rica | 278 (249 to 308) | 465 (407 to 520) | 9.75 (8.63 to 10.92) | 9.50 (8.37 to 10.65) | -0.37 (-0.77 to 0.04) |
| Croatia | 1380 (1214 to 1564) | 2647 (1870 to 3487) | 28.55 (25.17 to 32.29) | 36.43 (25.59 to 47.91) | -1.42 (-2.62 to -0.20) |
| Cuba | 615 (537 to 685) | 463 (404 to 536) | 6.12 (5.33 to 6.84) | 4.33 (3.74 to 5.01) | -1.27 (-1.60 to -0.94) |
| Cyprus | 69 (37 to 100) | 45 (30 to 77) | 10.10 (5.12 to 15.05) | 3.00 (2.04 to 4.91) | -4.60 (-5.02 to -4.18) |
| Czechia | 680 (590 to 771) | 1171 (811 to 1607) | 6.21 (5.43 to 7.03) | 7.62 (5.27 to 10.51) | 0.08 (-0.25 to 0.40) |
| Côte d'Ivoire | 2189 (1262 to 4147) | 2314 (1243 to 3910) | 14.12 (8.75 to 22.72) | 7.10 (4.08 to 12.73) | -2.51 (-2.80 to -2.22) |
| Democratic People's Republic of Korea | 5995 (4099 to 8852) | 6209 (4051 to 10227) | 31.62 (21.41 to 46.57) | 25.75 (16.88 to 42.46) | -0.51 (-0.71 to -0.30) |
| Democratic Republic of the Congo | 7494 (3700 to 12881) | 7919 (4159 to 16122) | 17.05 (8.95 to 26.18) | 10.43 (5.05 to 24.49) | -1.54 (-1.65 to -1.44) |
| Denmark | 187 (169 to 207) | 323 (276 to 372) | 3.61 (3.22 to 4.00) | 4.06 (3.58 to 4.58) | 0.75 (-0.91 to 2.45) |
| Djibouti | 52 (22 to 93) | 56 (30 to 110) | 10.47 (5.25 to 16.91) | 4.52 (2.36 to 9.03) | -3.05 (-3.30 to -2.80) |
| Dominica | 14 (11 to 21) | 13 (9 to 18) | 21.00 (15.73 to 30.80) | 19.93 (14.07 to 27.54) | -0.01 (-0.20 to 0.19) |
| Dominican Republic | 425 (264 to 736) | 479 (287 to 684) | 4.76 (3.15 to 8.00) | 4.44 (2.67 to 6.38) | 0.02 (-0.13 to 0.16) |
| Ecuador | 662 (523 to 800) | 392 (268 to 553) | 7.41 (5.83 to 8.91) | 2.33 (1.61 to 3.26) | -5.06 (-5.77 to -4.35) |
| Egypt | 1024 (340 to 3264) | 629 (296 to 1299) | 1.46 (0.50 to 4.38) | 0.60 (0.28 to 1.28) | -2.90 (-3.00 to -2.79) |
| El Salvador | 221 (151 to 316) | 123 (79 to 181) | 3.52 (2.38 to 4.88) | 1.92 (1.22 to 2.83) | -1.95 (-2.25 to -1.64) |
| Equatorial Guinea | 85 (40 to 148) | 76 (41 to 140) | 18.16 (9.09 to 29.43) | 5.73 (3.18 to 10.76) | -4.49 (-4.85 to -4.13) |
| Eritrea | 621 (259 to 1187) | 438 (244 to 762) | 14.40 (6.51 to 27.00) | 6.56 (3.49 to 12.02) | -2.64 (-2.82 to -2.47) |
| Estonia | 73 (56 to 93) | 42 (34 to 52) | 4.49 (3.55 to 5.66) | 1.86 (1.48 to 2.32) | -3.40 (-3.91 to -2.89) |
| Eswatini | 100 (63 to 152) | 83 (49 to 138) | 12.42 (7.71 to 19.41) | 7.33 (4.46 to 12.32) | -1.70 (-1.78 to -1.62) |
| Ethiopia | 8081 (3374 to 17666) | 4632 (2834 to 8013) | 11.77 (5.35 to 24.19) | 4.27 (2.47 to 8.43) | -3.65 (-3.82 to -3.48) |
| Fiji | 87 (66 to 119) | 106 (69 to 157) | 10.57 (8.07 to 14.68) | 11.64 (7.54 to 17.16) | 0.76 (0.49 to 1.04) |
| Finland | 666 (588 to 756) | 792 (683 to 894) | 12.78 (11.28 to 14.37) | 10.82 (9.59 to 12.10) | -0.15 (-1.57 to 1.29) |
| France | 500 (434 to 584) | 2021 (1768 to 2305) | 0.82 (0.72 to 0.95) | 2.21 (1.95 to 2.49) | 3.77 (1.81 to 5.77) |
| Gabon | 121 (64 to 194) | 96 (55 to 170) | 12.80 (6.98 to 20.65) | 5.95 (3.40 to 10.59) | -2.52 (-2.62 to -2.43) |
| Gambia | 148 (90 to 252) | 180 (80 to 356) | 12.76 (7.84 to 20.56) | 6.95 (3.43 to 14.04) | -2.53 (-2.85 to -2.21) |
| Georgia | 1493 (1105 to 1895) | 996 (679 to 1370) | 25.99 (19.16 to 32.99) | 20.48 (14.12 to 28.41) | -1.32 (-2.24 to -0.40) |
| Germany | 4569 (3418 to 5715) | 7157 (6103 to 8217) | 5.16 (4.04 to 6.33) | 6.68 (5.75 to 7.59) | 1.15 (-0.13 to 2.45) |
| Ghana | 3211 (2215 to 4766) | 3133 (1863 to 5798) | 18.83 (12.28 to 27.83) | 8.84 (5.13 to 15.86) | -2.74 (-2.99 to -2.49) |
| Greece | 120 (104 to 137) | 362 (311 to 420) | 1.12 (0.99 to 1.26) | 2.65 (2.26 to 3.11) | 3.43 (1.26 to 5.64) |
| Greenland | 9 (5 to 14) | 5 (2 to 8) | 15.55 (8.89 to 24.56) | 11.09 (4.84 to 16.67) | -0.99 (-1.27 to -0.71) |
| Grenada | 15 (12 to 19) | 17 (15 to 20) | 16.78 (14.10 to 21.86) | 16.82 (14.58 to 19.12) | -0.17 (-0.50 to 0.15) |
| Guam | 22 (16 to 28) | 12 (8 to 17) | 14.76 (11.15 to 19.42) | 8.23 (6.02 to 12.18) | -0.95 (-1.33 to -0.56) |
| Guatemala | 789 (587 to 986) | 1355 (1089 to 1747) | 7.40 (6.14 to 8.66) | 9.12 (7.37 to 11.76) | 0.72 (0.33 to 1.11) |
| Guinea | 1395 (714 to 2903) | 1345 (726 to 2468) | 16.35 (9.18 to 28.66) | 8.16 (4.51 to 15.05) | -2.40 (-2.54 to -2.25) |
| Guinea-Bissau | 235 (133 to 450) | 196 (100 to 400) | 18.60 (10.32 to 33.03) | 8.50 (4.50 to 17.40) | -2.72 (-2.88 to -2.57) |
| Guyana | 377 (321 to 438) | 585 (426 to 750) | 44.64 (38.12 to 51.67) | 79.85 (58.78 to 101.51) | 2.72 (2.43 to 3.01) |
| Haiti | 2837 (1208 to 7128) | 4421 (2303 to 7561) | 36.03 (16.41 to 77.57) | 32.86 (16.99 to 55.07) | -0.09 (-0.18 to 0.01) |
| Honduras | 280 (173 to 450) | 349 (187 to 596) | 5.67 (3.25 to 9.37) | 4.15 (2.30 to 6.75) | -0.82 (-0.90 to -0.74) |
| Hungary | 1105 (793 to 1449) | 970 (680 to 1351) | 10.03 (7.45 to 12.77) | 6.84 (4.81 to 9.44) | -1.54 (-1.84 to -1.24) |
| Iceland | 6 (5 to 7) | 21 (18 to 24) | 2.36 (2.14 to 2.62) | 4.89 (4.30 to 5.51) | 2.26 (0.46 to 4.10) |
| India | 112547 (64152 to 178695) | 125046 (84690 to 173732) | 13.59 (7.42 to 20.14) | 9.88 (6.75 to 13.67) | -0.79 (-0.91 to -0.68) |
| Indonesia | 15834 (9020 to 33699) | 20405 (13439 to 34678) | 9.80 (5.80 to 19.41) | 9.41 (6.65 to 15.10) | -0.18 (-0.31 to -0.04) |
| Iran (Islamic Republic of) | 4838 (2453 to 7836) | 4148 (2195 to 5386) | 11.61 (6.80 to 15.95) | 5.51 (2.96 to 7.14) | -1.60 (-2.20 to -1.01) |
| Iraq | 10455 (6663 to 15013) | 9528 (6551 to 12796) | 53.61 (36.98 to 73.78) | 29.24 (20.72 to 39.57) | -2.03 (-2.17 to -1.89) |
| Ireland | 127 (117 to 138) | 583 (502 to 672) | 3.70 (3.45 to 4.03) | 9.57 (8.29 to 10.98) | 3.68 (1.62 to 5.78) |
| Israel | 108 (94 to 125) | 259 (225 to 299) | 2.25 (1.95 to 2.60) | 2.47 (2.14 to 2.86) | 0.80 (-1.26 to 2.91) |
| Italy | 33347 (27589 to 37833) | 8078 (6309 to 9436) | 47.11 (39.15 to 53.31) | 5.02 (4.01 to 5.94) | -8.35 (-9.97 to -6.71) |
| Jamaica | 141 (117 to 168) | 174 (134 to 224) | 5.82 (4.76 to 7.00) | 6.32 (4.81 to 8.17) | 0.77 (0.41 to 1.13) |
| Japan | 8846 (8484 to 9269) | 11113 (10174 to 12214) | 8.00 (7.70 to 8.36) | 8.38 (7.73 to 9.15) | -0.74 (-1.53 to 0.05) |
| Jordan | 195 (124 to 284) | 236 (167 to 316) | 5.26 (3.47 to 7.32) | 2.44 (1.74 to 3.25) | -2.80 (-3.02 to -2.57) |
| Kazakhstan | 634 (446 to 909) | 15549 (11398 to 21479) | 4.13 (2.86 to 6.00) | 79.28 (58.87 to 109.29) | 12.37 (10.19 to 14.60) |
| Kenya | 1394 (805 to 2159) | 1372 (754 to 2864) | 5.17 (3.30 to 7.35) | 3.11 (1.59 to 6.70) | -1.56 (-1.62 to -1.51) |
| Kiribati | 24 (14 to 38) | 38 (21 to 59) | 31.09 (17.54 to 46.40) | 31.68 (17.62 to 48.75) | -0.03 (-0.08 to 0.02) |
| Kuwait | 163 (137 to 194) | 257 (215 to 304) | 11.40 (9.88 to 13.27) | 8.56 (7.24 to 10.12) | -1.25 (-1.95 to -0.54) |
| Kyrgyzstan | 606 (492 to 717) | 1008 (847 to 1219) | 13.48 (11.00 to 16.45) | 16.51 (13.41 to 20.07) | 1.29 (0.30 to 2.29) |
| Lao People's Democratic Republic | 638 (247 to 1990) | 755 (421 to 1348) | 15.47 (6.21 to 35.49) | 11.68 (6.98 to 20.22) | -0.96 (-1.09 to -0.84) |
| Latvia | 82 (67 to 101) | 77 (59 to 98) | 2.89 (2.42 to 3.53) | 2.76 (2.07 to 3.51) | -0.92 (-1.32 to -0.52) |
| Lebanon | 431 (239 to 729) | 444 (331 to 649) | 16.50 (9.21 to 28.68) | 7.69 (5.74 to 10.93) | -2.73 (-2.85 to -2.60) |
| Lesotho | 149 (95 to 224) | 129 (81 to 201) | 10.40 (6.34 to 16.62) | 7.49 (4.69 to 11.97) | -1.02 (-1.12 to -0.93) |
| Liberia | 669 (362 to 1507) | 432 (213 to 828) | 19.52 (10.85 to 37.36) | 7.26 (3.78 to 14.37) | -3.36 (-3.66 to -3.07) |
| Libya | 906 (206 to 2733) | 514 (106 to 1709) | 18.17 (4.16 to 54.94) | 10.69 (2.25 to 35.81) | -1.58 (-1.77 to -1.39) |
| Lithuania | 97 (72 to 125) | 80 (61 to 100) | 2.54 (1.93 to 3.26) | 1.89 (1.44 to 2.38) | -1.26 (-1.79 to -0.73) |
| Luxembourg | 10 (9 to 11) | 34 (29 to 38) | 2.60 (2.37 to 2.85) | 4.06 (3.53 to 4.65) | 1.46 (0.42 to 2.51) |
| Madagascar | 3029 (1340 to 5552) | 2891 (1701 to 5129) | 19.09 (9.74 to 31.60) | 9.92 (5.70 to 20.02) | -2.10 (-2.15 to -2.05) |
| Malawi | 1542 (551 to 3136) | 879 (527 to 1565) | 10.26 (4.95 to 17.92) | 4.78 (2.76 to 9.58) | -2.62 (-2.76 to -2.48) |
| Malaysia | 1779 (1214 to 2443) | 1667 (1210 to 2157) | 10.65 (7.05 to 14.55) | 6.11 (4.45 to 7.85) | -2.02 (-2.24 to -1.81) |
| Maldives | 31 (15 to 60) | 26 (18 to 40) | 14.17 (7.73 to 23.62) | 6.62 (4.74 to 9.28) | -2.62 (-2.74 to -2.51) |
| Mali | 1535 (829 to 3065) | 1824 (939 to 3483) | 13.47 (7.06 to 24.22) | 6.18 (3.34 to 11.75) | -2.77 (-2.97 to -2.57) |
| Malta | 35 (31 to 38) | 109 (96 to 123) | 10.01 (9.04 to 10.98) | 18.19 (16.22 to 20.72) | 1.83 (-0.03 to 3.73) |
| Marshall Islands | 6 (3 to 10) | 6 (3 to 10) | 11.41 (5.49 to 20.83) | 9.77 (4.99 to 17.24) | -0.56 (-0.66 to -0.46) |
| Mauritania | 287 (176 to 486) | 246 (109 to 496) | 12.02 (7.22 to 20.46) | 5.29 (2.49 to 11.11) | -3.12 (-3.31 to -2.93) |
| Mauritius | 8 (7 to 9) | 53 (48 to 59) | 0.83 (0.73 to 0.96) | 4.15 (3.69 to 4.60) | 5.09 (3.18 to 7.04) |
| Mexico | 1564 (1430 to 1728) | 4767 (4108 to 5586) | 1.68 (1.55 to 1.86) | 4.11 (3.53 to 4.84) | 4.24 (3.69 to 4.79) |
| Micronesia (Federated States of) | 15 (7 to 27) | 9 (4 to 16) | 13.24 (6.23 to 24.80) | 8.89 (4.37 to 15.28) | -1.37 (-1.44 to -1.31) |
| Monaco | 3 (2 to 4) | 2 (1 to 3) | 7.89 (4.90 to 11.26) | 3.51 (2.24 to 4.94) | -2.89 (-3.15 to -2.62) |
| Mongolia | 1064 (573 to 1815) | 775 (492 to 1179) | 67.92 (33.91 to 125.80) | 26.10 (16.81 to 39.13) | -3.67 (-4.08 to -3.25) |
| Montenegro | 160 (99 to 242) | 117 (72 to 186) | 26.53 (16.58 to 39.38) | 15.65 (9.99 to 25.19) | -2.05 (-2.29 to -1.81) |
| Morocco | 4396 (813 to 14030) | 3784 (760 to 12453) | 18.36 (3.35 to 55.88) | 11.41 (2.28 to 37.84) | -1.43 (-1.49 to -1.38) |
| Mozambique | 1506 (562 to 3122) | 1277 (721 to 2010) | 8.31 (3.85 to 14.58) | 4.38 (2.23 to 8.03) | -1.90 (-1.97 to -1.83) |
| Myanmar | 5889 (2721 to 14619) | 6254 (3928 to 11158) | 16.17 (7.81 to 33.93) | 12.59 (8.20 to 21.48) | -1.03 (-1.20 to -0.85) |
| Namibia | 137 (91 to 209) | 148 (83 to 258) | 10.52 (7.12 to 16.25) | 6.59 (3.84 to 11.15) | -1.69 (-1.78 to -1.60) |
| Nauru | 1 (1 to 2) | 1 (1 to 2) | 10.75 (4.57 to 17.99) | 11.25 (5.69 to 20.07) | -0.05 (-0.37 to 0.27) |
| Nepal | 3321 (1771 to 5838) | 3297 (1983 to 5340) | 15.98 (8.37 to 25.49) | 11.69 (6.96 to 19.05) | -0.88 (-0.99 to -0.77) |
| Netherlands | 315 (290 to 347) | 810 (698 to 922) | 2.12 (1.95 to 2.33) | 3.51 (3.07 to 3.97) | 1.74 (-0.34 to 3.86) |
| New Zealand | 764 (706 to 829) | 795 (731 to 872) | 23.22 (21.47 to 25.27) | 15.74 (14.50 to 17.20) | -1.14 (-1.92 to -0.35) |
| Nicaragua | 224 (125 to 338) | 151 (102 to 211) | 4.35 (2.86 to 6.01) | 2.43 (1.64 to 3.36) | -1.64 (-1.83 to -1.44) |
| Niger | 1722 (812 to 3858) | 2112 (1137 to 3800) | 15.76 (7.95 to 28.74) | 6.93 (3.64 to 13.79) | -3.04 (-3.31 to -2.76) |
| Nigeria | 18608 (11257 to 38903) | 18183 (11902 to 32460) | 16.06 (9.09 to 28.46) | 6.27 (4.20 to 10.92) | -3.52 (-3.85 to -3.18) |
| Niue | 0 (0 to 0) | 0 (0 to 0) | 9.88 (4.99 to 16.77) | 16.14 (8.58 to 24.97) | -0.43 (-1.16 to 0.32) |
| North Macedonia | 272 (185 to 420) | 272 (129 to 511) | 15.11 (10.29 to 22.79) | 10.76 (5.51 to 19.73) | -2.09 (-2.45 to -1.72) |
| Northern Mariana Islands | 3 (2 to 4) | 3 (2 to 6) | 5.36 (3.69 to 7.64) | 7.14 (4.00 to 11.25) | 0.70 (0.32 to 1.07) |
| Norway | 295 (280 to 311) | 289 (266 to 316) | 7.38 (7.03 to 7.78) | 4.57 (4.24 to 4.96) | -0.69 (-1.78 to 0.42) |
| Oman | 381 (213 to 644) | 420 (227 to 684) | 24.71 (14.77 to 39.21) | 14.27 (8.09 to 23.36) | -1.65 (-2.00 to -1.30) |
| Pakistan | 21358 (12005 to 34591) | 36279 (23306 to 53074) | 16.73 (9.84 to 26.57) | 15.83 (10.09 to 24.36) | 0.23 (0.09 to 0.37) |
| Palau | 1 (1 to 2) | 1 (0 to 1) | 7.55 (3.52 to 12.00) | 5.08 (2.83 to 7.41) | -1.27 (-1.34 to -1.20) |
| Palestine | 185 (115 to 300) | 225 (115 to 320) | 8.47 (5.53 to 12.39) | 5.82 (2.81 to 8.09) | -1.36 (-1.50 to -1.22) |
| Panama | 76 (63 to 90) | 161 (134 to 193) | 3.14 (2.61 to 3.68) | 3.88 (3.22 to 4.68) | 0.54 (0.15 to 0.93) |
| Papua New Guinea | 778 (348 to 1461) | 1906 (925 to 3408) | 14.19 (6.22 to 26.62) | 14.53 (7.31 to 25.76) | 0.10 (0.01 to 0.19) |
| Paraguay | 230 (153 to 307) | 187 (132 to 276) | 4.98 (3.29 to 6.58) | 2.84 (2.01 to 4.20) | -1.96 (-2.11 to -1.81) |
| Peru | 1390 (708 to 2408) | 553 (388 to 827) | 6.94 (3.78 to 10.91) | 1.58 (1.11 to 2.36) | -5.05 (-5.26 to -4.84) |
| Philippines | 3943 (2313 to 5579) | 4643 (3164 to 5730) | 6.38 (3.98 to 8.03) | 4.64 (3.01 to 5.74) | -0.69 (-0.87 to -0.51) |
| Poland | 6643 (6217 to 7123) | 4656 (3714 to 5718) | 19.31 (18.11 to 20.77) | 9.47 (7.66 to 11.62) | -2.57 (-2.97 to -2.18) |
| Portugal | 132 (121 to 147) | 385 (326 to 438) | 1.48 (1.35 to 1.62) | 2.55 (2.22 to 2.90) | 1.98 (0.07 to 3.93) |
| Puerto Rico | 60 (51 to 69) | 103 (87 to 123) | 1.71 (1.45 to 1.96) | 2.89 (2.44 to 3.45) | 1.26 (-0.12 to 2.66) |
| Qatar | 28 (18 to 46) | 86 (48 to 173) | 11.93 (7.56 to 18.09) | 5.82 (3.16 to 11.76) | -2.67 (-3.01 to -2.33) |
| Republic of Korea | 6697 (4612 to 8938) | 2869 (2113 to 3842) | 17.26 (11.70 to 23.58) | 6.59 (4.83 to 8.52) | -3.07 (-3.31 to -2.84) |
| Republic of Moldova | 242 (205 to 283) | 187 (143 to 238) | 5.88 (5.00 to 6.80) | 4.01 (3.06 to 5.14) | -1.78 (-2.26 to -1.29) |
| Romania | 19127 (14959 to 24816) | 41704 (30998 to 53754) | 86.61 (70.89 to 109.09) | 141.03 (104.45 to 182.00) | 0.91 (0.34 to 1.48) |
| Russian Federation | 15959 (14904 to 16936) | 26246 (22831 to 29531) | 11.93 (11.16 to 12.65) | 14.77 (12.79 to 16.66) | 0.15 (-0.48 to 0.79) |
| Rwanda | 1540 (606 to 3268) | 648 (382 to 1109) | 15.85 (7.37 to 30.29) | 4.97 (2.79 to 8.99) | -4.61 (-4.93 to -4.29) |
| Saint Kitts and Nevis | 1 (1 to 2) | 3 (3 to 4) | 3.45 (2.91 to 4.61) | 5.52 (4.33 to 7.11) | 1.70 (1.29 to 2.10) |
| Saint Lucia | 33 (29 to 38) | 40 (32 to 48) | 27.59 (24.17 to 31.25) | 21.23 (17.21 to 25.88) | -0.96 (-1.12 to -0.79) |
| Saint Vincent and the Grenadines | 9 (8 to 11) | 16 (13 to 19) | 8.38 (7.26 to 9.56) | 14.10 (11.77 to 16.69) | 0.93 (0.08 to 1.80) |
| Samoa | 20 (11 to 32) | 16 (9 to 25) | 10.19 (5.23 to 17.57) | 6.83 (3.79 to 10.36) | -1.19 (-1.27 to -1.12) |
| San Marino | 2 (1 to 4) | 3 (2 to 5) | 8.50 (5.76 to 13.54) | 6.04 (3.82 to 8.49) | 0.21 (-0.16 to 0.59) |
| Sao Tome and Principe | 19 (11 to 35) | 12 (4 to 33) | 13.09 (7.84 to 20.81) | 5.71 (2.10 to 15.01) | -2.78 (-3.04 to -2.52) |
| Saudi Arabia | 3829 (2256 to 5960) | 2639 (1734 to 3913) | 29.29 (18.69 to 42.47) | 9.73 (6.47 to 14.57) | -4.10 (-4.38 to -3.82) |
| Senegal | 1533 (951 to 2794) | 1285 (573 to 2677) | 15.35 (9.61 to 25.33) | 7.38 (3.58 to 15.57) | -2.42 (-2.58 to -2.26) |
| Serbia | 1292 (768 to 1840) | 765 (475 to 1093) | 14.94 (9.10 to 21.48) | 6.23 (4.14 to 9.23) | -3.33 (-3.59 to -3.06) |
| Seychelles | 15 (11 to 20) | 15 (11 to 20) | 21.90 (16.32 to 29.28) | 13.80 (10.31 to 18.09) | -1.00 (-1.17 to -0.83) |
| Sierra Leone | 1161 (566 to 2606) | 989 (456 to 2001) | 18.68 (9.64 to 36.33) | 9.02 (4.41 to 18.12) | -2.79 (-3.03 to -2.55) |
| Singapore | 2736 (2555 to 2910) | 1723 (1516 to 1908) | 88.12 (82.62 to 93.51) | 27.78 (24.65 to 30.66) | -4.00 (-4.34 to -3.66) |
| Slovakia | 425 (312 to 625) | 469 (293 to 687) | 8.19 (6.04 to 11.94) | 6.73 (4.30 to 9.73) | -0.65 (-0.76 to -0.54) |
| Slovenia | 81 (67 to 96) | 124 (84 to 169) | 3.68 (3.04 to 4.37) | 3.21 (2.12 to 4.47) | -1.69 (-2.28 to -1.09) |
| Solomon Islands | 31 (15 to 56) | 46 (25 to 77) | 7.35 (3.46 to 13.27) | 6.24 (3.27 to 10.73) | -0.49 (-0.57 to -0.41) |
| Somalia | 1877 (734 to 3844) | 2171 (987 to 4027) | 16.79 (7.29 to 31.77) | 8.96 (3.76 to 19.43) | -1.98 (-2.15 to -1.82) |
| South Africa | 3880 (2094 to 5273) | 2755 (2095 to 4460) | 10.65 (5.91 to 14.30) | 5.10 (3.91 to 8.11) | -2.82 (-3.05 to -2.59) |
| South Sudan | 1208 (300 to 2524) | 1108 (467 to 2304) | 14.35 (5.04 to 26.33) | 8.79 (3.97 to 15.55) | -1.66 (-2.24 to -1.07) |
| Spain | 585 (528 to 643) | 3218 (2567 to 3844) | 1.52 (1.38 to 1.65) | 4.09 (3.48 to 4.71) | 3.44 (1.51 to 5.40) |
| Sri Lanka | 12070 (7062 to 15329) | 4230 (2736 to 6637) | 86.40 (51.52 to 110.02) | 19.30 (12.72 to 30.03) | -6.50 (-7.33 to -5.66) |
| Sudan | 4447 (635 to 17163) | 4050 (740 to 13151) | 20.75 (3.10 to 68.11) | 11.94 (2.11 to 39.53) | -1.75 (-1.83 to -1.67) |
| Suriname | 33 (21 to 46) | 39 (25 to 52) | 8.36 (5.49 to 11.34) | 7.02 (4.48 to 9.72) | -0.25 (-0.41 to -0.10) |
| Sweden | 2814 (2618 to 3020) | 1934 (1610 to 2270) | 29.75 (27.84 to 31.56) | 15.38 (12.94 to 18.01) | -2.13 (-2.74 to -1.53) |
| Switzerland | 131 (114 to 151) | 231 (188 to 278) | 1.92 (1.66 to 2.24) | 1.80 (1.50 to 2.14) | -0.11 (-1.71 to 1.52) |
| Syrian Arab Republic | 2292 (488 to 6533) | 1333 (244 to 4674) | 17.84 (3.62 to 53.29) | 10.83 (1.96 to 38.23) | -1.90 (-2.26 to -1.54) |
| Taiwan (Province of China) | 1092 (1016 to 1164) | 3778 (3348 to 4183) | 6.42 (5.95 to 6.84) | 14.10 (12.62 to 15.57) | 5.28 (4.20 to 6.37) |
| Tajikistan | 23 (15 to 34) | 39 (26 to 56) | 0.49 (0.33 to 0.68) | 0.43 (0.29 to 0.63) | -0.57 (-0.76 to -0.39) |
| Thailand | 3536 (2405 to 5156) | 4665 (3033 to 6150) | 7.37 (4.93 to 10.60) | 6.68 (4.34 to 8.62) | -0.60 (-0.96 to -0.25) |
| Timor-Leste | 103 (45 to 297) | 159 (90 to 295) | 13.17 (5.98 to 27.73) | 12.49 (7.10 to 23.47) | -0.17 (-0.34 to 0.01) |
| Togo | 627 (371 to 1044) | 572 (303 to 1054) | 13.99 (8.84 to 21.56) | 6.60 (3.64 to 12.57) | -2.74 (-2.94 to -2.55) |
| Tokelau | 0 (0 to 0) | 0 (0 to 0) | 10.81 (4.99 to 19.13) | 21.59 (10.27 to 35.58) | -0.51 (-1.55 to 0.54) |
| Tonga | 7 (4 to 11) | 5 (3 to 9) | 6.04 (3.27 to 9.55) | 4.57 (2.33 to 7.44) | -0.86 (-0.93 to -0.79) |
| Trinidad and Tobago | 273 (246 to 305) | 317 (242 to 409) | 24.34 (22.09 to 27.24) | 22.36 (17.13 to 28.43) | -0.26 (-0.38 to -0.14) |
| Tunisia | 965 (221 to 2971) | 941 (186 to 3392) | 13.63 (3.14 to 40.53) | 7.93 (1.56 to 28.79) | -1.77 (-1.86 to -1.69) |
| Turkmenistan | 947 (783 to 1200) | 2013 (1544 to 2571) | 30.81 (25.13 to 39.62) | 40.17 (30.92 to 51.62) | 1.02 (-0.30 to 2.37) |
| Tuvalu | 1 (1 to 2) | 1 (1 to 2) | 12.76 (6.13 to 22.63) | 7.92 (4.28 to 12.23) | -1.54 (-1.62 to -1.46) |
| Türkiye | 2320 (1062 to 4369) | 1930 (1171 to 2624) | 4.21 (1.87 to 7.52) | 2.44 (1.47 to 3.35) | -1.67 (-1.97 to -1.37) |
| Uganda | 3326 (1174 to 6904) | 2384 (1378 to 3802) | 12.12 (5.69 to 21.59) | 4.88 (2.76 to 7.92) | -3.22 (-3.39 to -3.06) |
| Ukraine | 4816 (3629 to 5995) | 7160 (5225 to 9621) | 9.10 (7.10 to 11.12) | 12.72 (9.49 to 16.75) | 1.08 (0.87 to 1.30) |
| United Arab Emirates | 273 (152 to 466) | 473 (338 to 694) | 22.90 (13.74 to 37.29) | 11.49 (8.25 to 15.44) | -0.37 (-1.10 to 0.35) |
| United Kingdom | 11707 (11324 to 12081) | 9241 (8197 to 10145) | 20.46 (19.89 to 21.08) | 11.95 (10.61 to 13.14) | -2.00 (-2.38 to -1.62) |
| United Republic of Tanzania | 3933 (1657 to 7323) | 3019 (1788 to 5335) | 11.17 (6.12 to 18.01) | 4.86 (2.83 to 9.78) | -2.79 (-2.89 to -2.69) |
| United States Virgin Islands | 29 (21 to 45) | 23 (14 to 34) | 28.48 (20.65 to 43.27) | 27.90 (16.75 to 43.05) | 0.75 (0.47 to 1.03) |
| United States of America | 42458 (40502 to 44707) | 36537 (34501 to 38626) | 17.70 (16.95 to 18.53) | 12.54 (11.74 to 13.35) | -1.14 (-1.56 to -0.72) |
| Uruguay | 402 (347 to 457) | 215 (191 to 240) | 13.37 (11.59 to 15.21) | 5.65 (5.01 to 6.35) | -3.30 (-3.70 to -2.89) |
| Uzbekistan | 2228 (1697 to 2790) | 2064 (1633 to 2571) | 11.86 (9.00 to 15.34) | 6.36 (5.00 to 7.91) | -1.81 (-2.49 to -1.12) |
| Vanuatu | 20 (11 to 32) | 33 (18 to 57) | 10.18 (5.64 to 17.53) | 9.64 (5.10 to 16.79) | -0.38 (-0.50 to -0.27) |
| Venezuela (Bolivarian Republic of) | 1021 (867 to 1225) | 1102 (850 to 1424) | 4.87 (4.10 to 5.81) | 4.39 (3.39 to 5.71) | -0.83 (-1.21 to -0.44) |
| Viet Nam | 5537 (3418 to 10031) | 6302 (3777 to 10468) | 9.77 (5.66 to 18.05) | 7.22 (4.36 to 11.59) | -1.03 (-1.12 to -0.94) |
| Yemen | 2666 (412 to 10380) | 3541 (653 to 11551) | 20.29 (3.23 to 66.73) | 14.76 (2.63 to 49.86) | -1.13 (-1.22 to -1.05) |
| Zambia | 1154 (399 to 2737) | 1401 (535 to 3753) | 10.32 (4.63 to 22.25) | 8.63 (2.64 to 25.33) | -0.34 (-0.78 to 0.09) |
| Zimbabwe | 938 (633 to 1563) | 1988 (1148 to 3431) | 10.24 (6.76 to 19.54) | 13.35 (7.94 to 23.76) | 1.30 (1.06 to 1.54) |

# Figure S1: Correlation of EAPC in the age-standardized incidence (A), prevalence (B), death (C) and DALYs (D) rates of myocarditis from 1990 to 2021 with different SDI in 204 countries and territories.


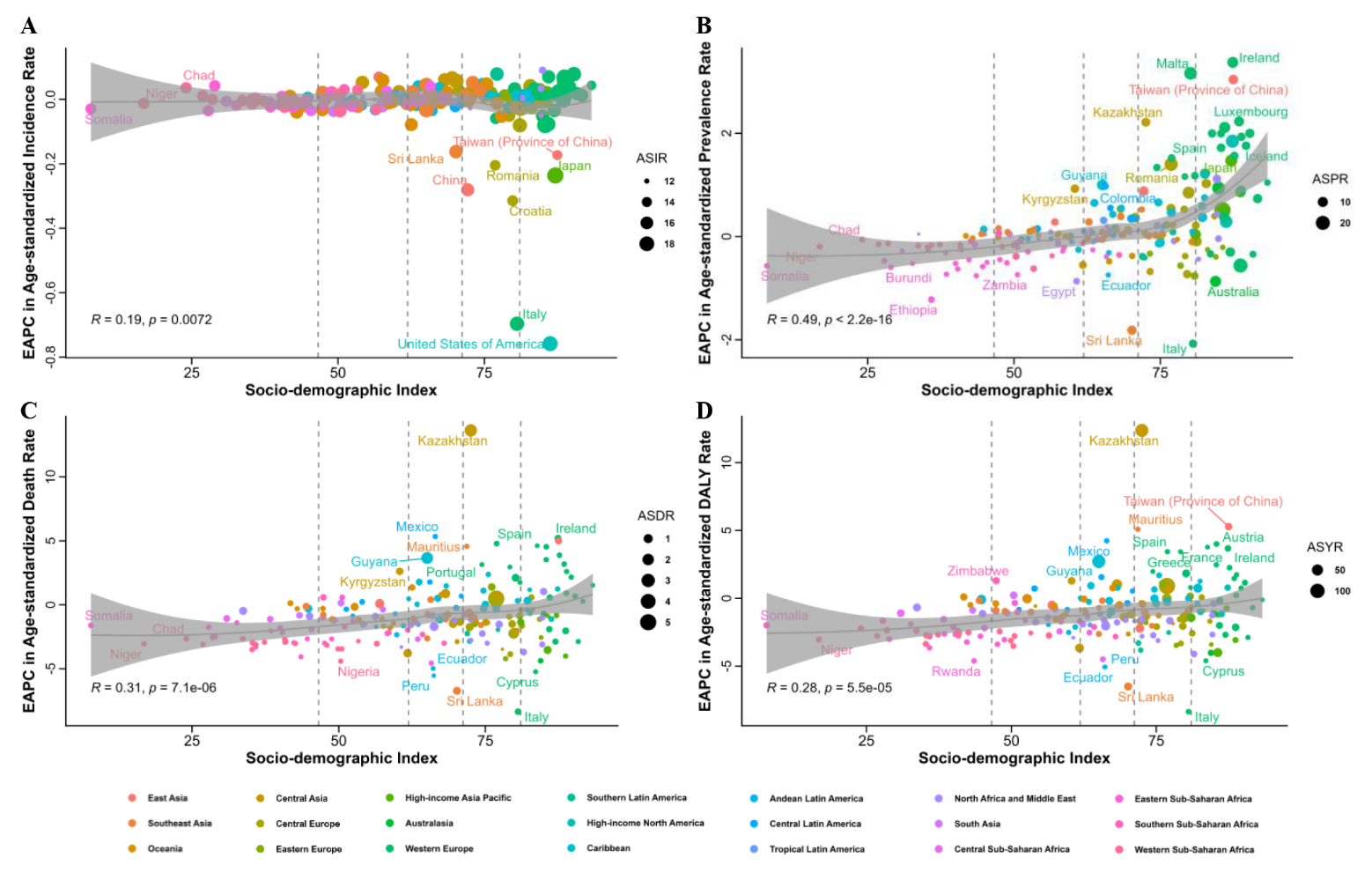


DALYs, disability-adjusted life years; SDI, socio-demographic index.

# Figure S2: Incidence (A), prevalence (B), death (C) and DALYs (D) rates of myocarditis by age (＜70 years or 70+years) and sex from 1990 to 2021 at the global level.

**
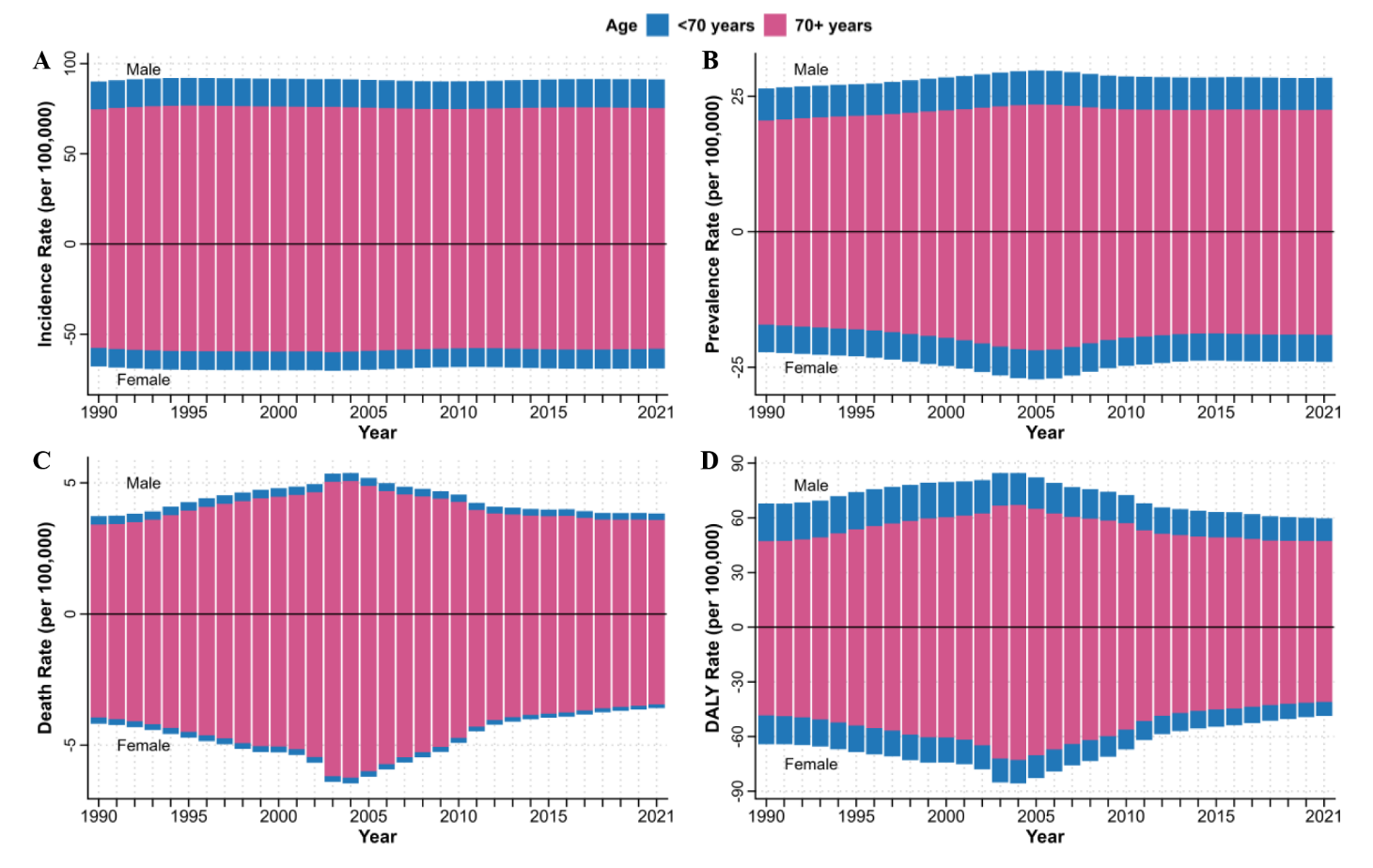
**

DALYs, disability-adjusted life years.

# Figure S3: Age-standardized death and DALY rates of myocarditis attributed to non-optimal temperature by sex from 1990 to 2021 at the global level.

**
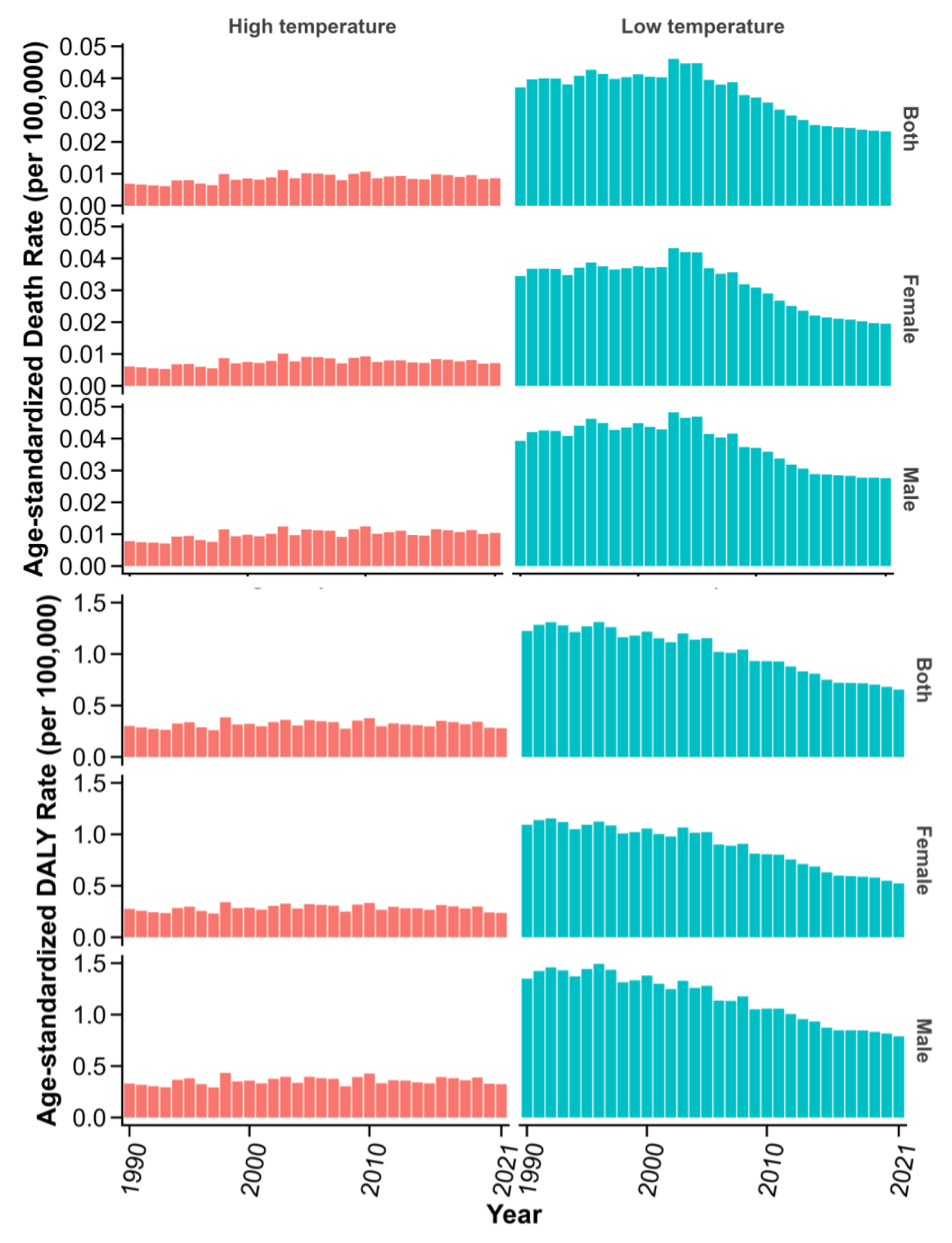
**

DALYs, disability-adjusted life years.

# Figure S4: Age-standardized death and DALY rates of myocarditis attributed to non-optimal temperature by age and sex in 2021 at the global level.

**
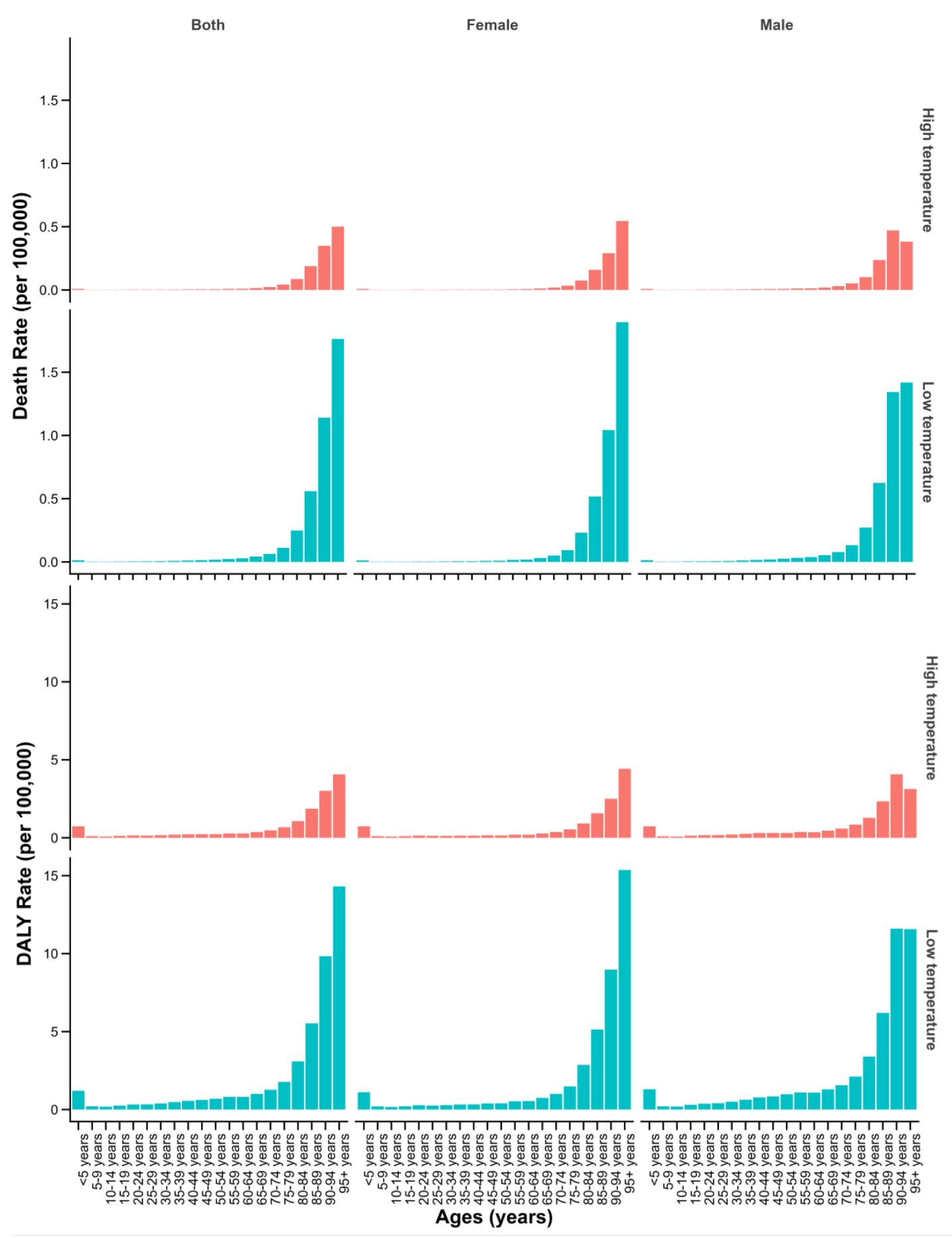
**

DALYs, disability-adjusted life years.

# **Figure S5: Effect of non-optimal temperature as a risk factor the age-standardized death and DALY rates of myocarditis with different SDI quintiles from 1990 to 2021.**
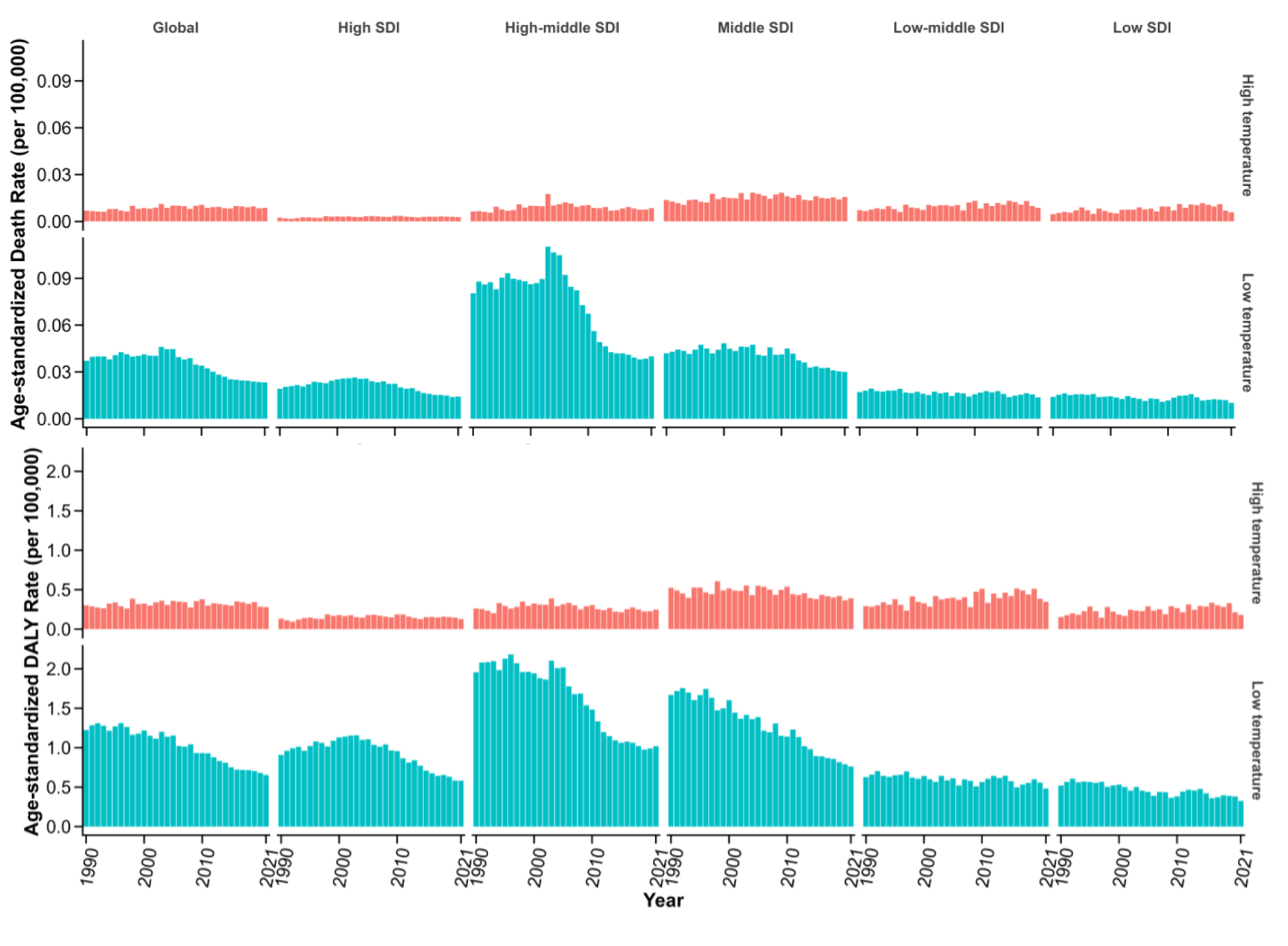


DALYs, disability-adjusted life years; SDI, socio-demographic index.

# Figure S6: Age-standardized death and DALY rates of myocarditis attributed to non-optimal temperature by sex in 21 GBD regions in 2021.

**
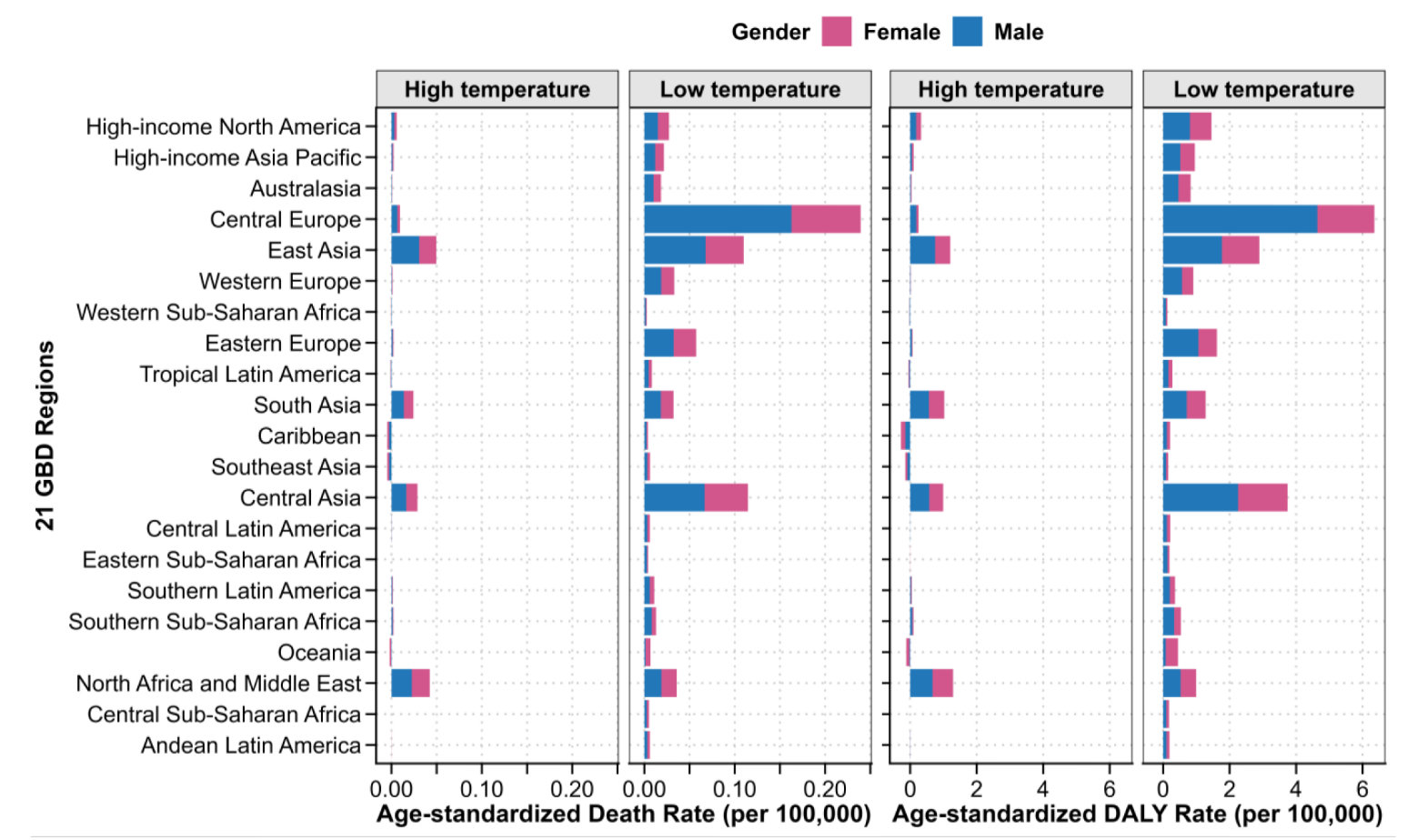
**

DALYs, disability-adjusted life years; GBD, Global Burden of Diseases, Injuries, and Risk Factors Study.
